# Supplementary material for: Metagenome-Based Analysis of the Microbial Community Structure and Drug-Resistance Characteristics of Livestock Feces in Anhui Province, China
Source: Vet Sci. 2024 Feb 12;11(2):87. doi: 10.3390/vetsci11020087 (PMC10892912; doi:10.3390/vetsci11020087)
Supplement: Supplementary file 1 [file vetsci-11-00087-s001.zip › vetsci-2749646-supplementary.pdf]

ARO_accession	ARO_name	ARO_description	AMR Gene Family	Drug Class	Antibiotic class	Resistance Mechanism	HB_3_2	HB_3_1	XZ_2_2	BG_1_3	XZ_2_1	HB_3_3	BG_1_1	BG_1_2	XZ_2_3	SC_2_3	SC_2_1	SC_2_2	Total
ARO:3000005	vanD	VanD is a D-Ala-D-Ala ligase homolog similar to VanA, and can synthesize D-Ala-D-Lac, an alternative substrate for peptidoglycan synthesis that reduces vancomycin binding affinity. It is associated with both vancomycin and teicoplanin resistance.	glycopeptide resistance gene cluster;van ligase	glycopeptide antibiotic	Glycopeptide	Antibiotic target alteration	186	182	378	142	100	182	168	136	178	294	254	270	2470
ARO:3000010	vanA	VanA is a D-Ala-D-Ala ligase homolog that synthesizes D-Ala-D-Lac, an alternative substrate for peptidoglycan synthesis that reduces vancomycin binding affinity. It has been isolated from VREs. It is associated with both vancomycin and teicoplanin resistance.	glycopeptide resistance gene cluster;van ligase	glycopeptide antibiotic	Glycopeptide	Antibiotic target alteration	580	340	660	328	906	454	346	570	994	852	1172	1020	8222
ARO:3000013	vanB	VanB is a D-Ala-D-Ala ligase homolog similar to VanA, and can synthesize D-Ala-D-Lac, an alternative substrate for peptidoglycan synthesis that reduces vancomycin binding affinity. It has been isolated from VREs. It is associated with vancomycin resistance, but not teicoplanin resistance.	glycopeptide resistance gene cluster;van ligase	glycopeptide antibiotic	Glycopeptide	Antibiotic target alteration	876	84	32	82	12	1016	60	52	70	158	314	122	2878
ARO:3000024	patA	PatA is an ABC transporter of Streptococcus pneumoniae that interacts with PatB to confer fluoroquinolone resistance.	ATP-binding cassette (ABC) antibiotic efflux pump	fluoroquinolone antibiotic	Fluoroquinolone	Antibiotic efflux	6780	5618	6626	5262	10274	6004	6540	6898	10718	5460	5064	5244	80488
ARO:3000025	patB	PatB is an ABC transporter of Streptococcus pneumoniae that interacts with PatA to confer fluoroquinolone resistance..	ATP-binding cassette (ABC) antibiotic efflux pump	fluoroquinolone antibiotic	Fluoroquinolone	Antibiotic efflux	5802	7748	5240	3008	2502	7904	2818	3382	3198	3928	3860	4336	53726
ARO:3000026	mepA	MepA is an efflux protein regulated by MepR and part of the MepRAB cluster. Its presence in Staphylococcus aureus led to multidrug resistance, while it has also	multidrug and toxic compound extrusion (MATE) transporter	glycylcycline;tetracycline antibiotic	Multidrug	Antibiotic efflux	908	624	1312	264	464	954	258	332	570	304	172	178	6340

		been shown to decrease tigecycline susceptibility.																	
ARO:3000027	emrA	EmrA is a membrane fusion protein, providing an efflux pathway with EmrB and TolC between the inner and outer membranes of E. coli, a Gram-negative bacterium.	major facilitator superfamily (MFS) antibiotic efflux pump	fluoroquinolone antibiotic	Fluoroquinolone	Antibiotic efflux	1060	652	1022	1034	728	488	1512	1116	588	288	302	186	8976
ARO:3000074	emrB	emrB is a translocase in the emrB -TolC efflux protein in E. coli. It recognizes substrates including carbonyl cyanide m-chlorophenylhydrazone (CCCP), nalidixic acid, and thioloactomycin.	major facilitator superfamily (MFS) antibiotic efflux pump	fluoroquinolone antibiotic	Fluoroquinolone	Antibiotic efflux	1018	280	470	1330	712	514	2030	1480	750	508	1030	434	10556
ARO:3000118	vgaB	Vga(B) is an ABC-F protein expressed in staphylococci that confers resistance to streptogramin A antibiotics and related compounds. It is associated with plasmid DNA.	ABC-F ATP-binding cassette ribosomal protection protein	lincosamide antibiotic;macrolide antibiotic;oxazolidinone antibiotic;phenicol antibiotic;pleuromutilin antibiotic;streptogramin antibiotic;tetracycline antibiotic	Multidrug	Antibiotic target protection	1642	2490	1988	910	516	1884	830	1048	638	994	802	868	14610
ARO:3000124	mecI	mecI acts as a repressor of transcription of the mecA/mecR1/mecI operon.	methicillin resistant PBP2	carbapenem;cephalosporin;cephamycin;monobactam;penam	Multidrug	Antibiotic target replacement	88	134	98	74	112	120	82	80	128	116	148	124	1304
ARO:3000149	FosA	An enzyme that confers resistance to fosfomycin in Serratia marcescens by breaking the epoxide ring of the molecule. It depends on the cofactors Manganese (II) and Potassium and uses Glutathione (GSH) as the nucleophilic molecule. In Pseudomonas aeruginosa, FosA catalyzes the conjugation of glutathione to carbon-1 of fosfomycin, rendering it ineffective as an antibacterial drug.	fosfomycin thiol transferase	fosfomycin	Fosfomycin	Antibiotic inactivation	0	2	20	14	14	0	8	10	4	18	18	12	120

ARO:3000165	tet(A)	TetA is a tetracycline efflux pump found in many species of Gram-negative bacteria.	major facilitator superfamily (MFS) antibiotic efflux pump	tetracycline antibiotic	Tetracycline	Antibiotic efflux	1402	524	336	2510	2680	390	3604	2834	2288	682	750	568	18568
ARO:3000166	tet(B)	Tet(B) is a tetracycline efflux protein expressed in many Gram-negative bacteria. It confers resistance to tetracycline, doxycycline, and minocycline, but not tigecycline.	major facilitator superfamily (MFS) antibiotic efflux pump	tetracycline antibiotic	Tetracycline	Antibiotic efflux	36	6	16	58	16	2	124	150	10	48	66	54	586
ARO:3000167	tet(C)	Tet(C) is a tetracycline efflux pump found in many species of Gram-negative bacteria. It is typically found in plasmid DNA.	major facilitator superfamily (MFS) antibiotic efflux pump	tetracycline antibiotic	Tetracycline	Antibiotic efflux	0	2	2	24	16	0	128	60	18	92	98	26	466
ARO:3000168	tet(D)	TetD is a tetracycline efflux pump found exclusively in Gram-negative bacteria.	major facilitator superfamily (MFS) antibiotic efflux pump	tetracycline antibiotic	Tetracycline	Antibiotic efflux	4	0	0	0	0	0	0	0	0	0	4	0	8
ARO:3000172	FosB	A thiol transferase that leads to the resistance of fosfomycin. Contrasting FosA, FosB is dependent on the cofactor Magnesium (II) and uses either bacillithiol or L-cysteine to open up the epoxide ring of fosfomycin.	fosfomycin thiol transferase	fosfomycin	Fosfomycin	Antibiotic inactivation	28	2	0	0	0	20	2	6	0	0	0	0	58
ARO:3000173	tet(E)	TetE is a tetracycline efflux pump found in many Gram-negative bacteria, especially those in water environments. The gene is found on large plasmids.	major facilitator superfamily (MFS) antibiotic efflux pump	tetracycline antibiotic	Tetracycline	Antibiotic efflux	0	0	0	32	0	0	10	40	2	0	0	0	84
ARO:3000174	tet(G)	TetG is a tetracycline efflux protein found in Gram-negative bacteria. The encoding gene is found in both chromosomal and plasmid DNA where it is frequently linked to the floR, sul1, and cmlA9 genes which encode proteins that can confer florfenicol/chloramphenicol,	major facilitator superfamily (MFS) antibiotic efflux pump	tetracycline antibiotic	Tetracycline	Antibiotic efflux	10	64	200	200	648	26	580	318	808	2810	2820	4214	12698

		sulfamethoxazole, and chloramphenicol resistance, respectively.																	
ARO:3000175	tet(H)	TetH is a tetracycline efflux protein expressed in Gram-negative bacteria (Actinobacillus, Acinetobacter, Gallibacterium, Histophilus, Mannheimia, Moraxella, Pasteurella, and Psychrobacter). Its gene is linked to the resistance genes sul2, and strAB, which confer resistance to sulfamethoxazole and streptomycin, respectively.	major facilitator superfamily (MFS) antibiotic efflux pump	tetracycline antibiotic	Tetracycline	Antibiotic efflux	4	0	26	12	24	0	4	34	180	1446	1200	2076	5006
ARO:3000178	tet(K)	TetK is a tetracycline efflux protein found in both Gram-negative (Haemophilus and Gallibacterium) and Gram-positive (many species, including mycobacteria) bacteria.	major facilitator superfamily (MFS) antibiotic efflux pump	tetracycline antibiotic	Tetracycline	Antibiotic efflux	0	0	0	0	0	0	0	0	0	24	16	48	88
ARO:3000179	tet(L)	TetL is a tetracycline efflux protein found in many species of Gram-negative and Gram-positive bacteria.	major facilitator superfamily (MFS) antibiotic efflux pump	tetracycline antibiotic	Tetracycline	Antibiotic efflux	58	78	10	28	58	124	40	42	62	172	208	138	1018
ARO:3000180	tetA(P)	TetA(P) is a inner membrane tetracycline efflux protein found on the same operon as the ribosomal protection protein TetB(P). It is found in Clostridium, a Gram-positive bacterium.	major facilitator superfamily (MFS) antibiotic efflux pump	tetracycline antibiotic	Tetracycline	Antibiotic efflux	78	34	8	34	28	118	40	58	44	170	152	62	826
ARO:3000181	tet(V)	TetV is a tetracycline efflux protein that has been found in Mycolicibacterium smegmatis and Mycolicibacterium fortuitum.	major facilitator superfamily (MFS) antibiotic efflux pump	tetracycline antibiotic	Tetracycline	Antibiotic efflux	2	26	24	52	22	28	106	36	38	98	142	44	618
ARO:3000182	tet(Y)	TetY is a tetracycline efflux pump found in Gram-negative bacteria (Aeromonas and Escherichia). It is associated with plasmid DNA.	major facilitator superfamily (MFS) antibiotic efflux pump	tetracycline antibiotic	Tetracycline	Antibiotic efflux	106	196	310	38	408	222	70	16	280	582	672	744	3644

ARO:3000183	tet(Z)	TetZ is a tetracycline efflux protein found in Gram-positive bacteria (Corynebacterium and Lactobacillus). It is associated with plasmid DNA.	major facilitator superfamily (MFS) antibiotic efflux pump	tetracycline antibiotic	Tetracycline	Antibiotic efflux	54	22	22	28	108	46	130	68	168	556	1200	612	3014
ARO:3000186	tetM	TetM is a ribosomal protection protein that confers tetracycline resistance. It is found on transposable DNA elements and its horizontal transfer between bacterial species has been documented.	tetracycline-resistant ribosomal protection protein	tetracycline antibiotic	Tetracycline	Antibiotic target protection	2378	4030	1954	1550	2596	2592	2044	1986	2728	5750	5590	7276	40474
ARO:3000190	tetO	TetO is a ribosomal protection protein. It is associated with conjugative plasmids.	tetracycline-resistant ribosomal protection protein	tetracycline antibiotic	Tetracycline	Antibiotic target protection	1778	1734	1640	690	648	1618	758	798	966	352	394	304	11680
ARO:3000191	tetQ	TetQ is a ribosomal protection protein. Its gene is associated with a conjugative transposon and has been found in both Gram-positive and Gram-negative bacteria.	tetracycline-resistant ribosomal protection protein	tetracycline antibiotic	Tetracycline	Antibiotic target protection	5600	9880	10588	5326	2612	7342	3828	5830	4058	2174	1254	1056	59548
ARO:3000192	tetS	Tet(S) is a ribosomal protection protein found in Gram-positive and Gram-negative strains. It is similar to tet(M) and tet(O).	tetracycline-resistant ribosomal protection protein	tetracycline antibiotic	Tetracycline	Antibiotic target protection	3492	5166	2770	2444	2636	3358	2766	2746	2616	2406	1990	2806	35196
ARO:3000193	tetT	Tet(T) is a ribosomal protection protein of streptococci. It is similar to Tet(Q).	tetracycline-resistant ribosomal protection protein	tetracycline antibiotic	Tetracycline	Antibiotic target protection	6612	7960	5842	2862	3666	7690	3090	3622	4256	10416	8722	13714	78452
ARO:3000194	tetW	TetW is a ribosomal protection protein. It is associated with both conjugative and non conjugative DNA and has been found strains of Clostridioides difficile.	tetracycline-resistant ribosomal protection protein	tetracycline antibiotic	Tetracycline	Antibiotic target protection	4930	5306	4892	4798	2808	6120	5646	5340	3016	2554	2720	2052	50182
ARO:3000195	tetB(P)	TetB(P) is a tetracycline ribosomal protection protein found on the same operon as tetA(P), a tetracycline efflux protein.	tetracycline-resistant ribosomal protection protein	tetracycline antibiotic	Tetracycline	Antibiotic target protection	7536	8016	7818	5570	3746	8374	6512	6110	3954	5516	5518	6042	74712
ARO:3000196	tet32	Tet32 is a tetracycline resistance gene similar to Tet(O), and binds	tetracycline-resistant	tetracycline antibiotic	Tetracycline	Antibiotic target	950	1010	1314	334	288	730	388	440	420	312	494	276	6956

		to the ribosome to confer tetracycline resistance as a ribosomal protection protein.	ribosomal protection protein			protection													
ARO:3000197	tet36	Tet36 is a tetracycline resistance gene found in Bacteroides similar to Tet(Q), and binds to the ribosome to confer antibiotic resistance as a ribosomal protection protein.	tetracycline-resistant ribosomal protection protein	tetracycline antibiotic	Tetracycline	Antibiotic target protection	976	1316	1480	740	706	1488	902	822	824	2670	1934	2512	16370
ARO:3000198	FosX	FosX is an enzyme used to confer resistance to fosfomycin. It's dependent on the cofactor, manganese (II), and uses water to generate a vicinal diol.	fosfomycin thiol transferase	fosfomycin	Fosfomycin	Antibiotic inactivation	0	0	0	0	0	0	2	4	0	2	16	16	40
ARO:3000205	tetX	TetX is a flavin-dependent monooxygenase conferring resistance to tetracycline antibiotics. TetX hydroxylates position 11a of the tetraketide group thus inactivating the antibiotic.	tetracycline inactivation enzyme	glycylcycline;tetracycline antibiotic	Tetracycline	Antibiotic inactivation	526	642	568	356	398	610	486	536	432	1936	1938	2492	10920
ARO:3000206	emrK	emrK is a membrane fusion protein that is a homolog of EmrA. Together with the inner membrane transporter EmrY and the outer membrane channel TolC, it mediates multidrug efflux.	major facilitator superfamily (MFS) antibiotic efflux pump	tetracycline antibiotic	Multidrug	Antibiotic efflux	502	348	378	838	428	174	1120	990	364	156	112	56	5466
ARO:3000215	mecR1	mecR1 is a transmembrane spanning and signal transducing protein which in response to interaction with beta-lactam antibiotics results in upregulation of the mecA/mecR1/mecI operon.	methicillin resistant PBP2	carbapenem;cephalosporin;cephamycin;monobactam;penam	Beta-lactam	Antibiotic target replacement	8	14	24	12	4	20	2	4	20	18	6	8	140
ARO:3000216	acrB	Protein subunit of AcrA-AcrB-TolC multidrug efflux complex. AcrB functions as a herterotrimer which forms the inner membrane component and is primarily responsible for substrate recognition and energy transduction by acting as a drug/proton antiporter.	resistance-nodulation-cell division (RND) antibiotic efflux pump	cephalosporin;fluoroquinolone antibiotic;glycylcycline;penam;phenicol antibiotic;rifamycin antibiotic;tetracycline antibiotic;triclosan	Multidrug	Antibiotic efflux	578	352	264	1334	568	120	2394	1804	502	3106	2562	4508	18092

ARO:3000217	blaR1	blaR1 is a transmembrane spanning and signal transducing protein which in response to interaction with beta-lactam antibiotics results in upregulation of the blaZ/blaR1/blaI operon.	blaZ beta-lactamase	penam	Beta-lactam	Antibiotic inactivation	14	80	210	174	142	4	150	296	182	24	6	4	1286
ARO:3000230	ANT(2'')-Ia	Plasmid or integron-encoded nucleotidylylation of 2-deoxystreptamine aminoglycosides at the hydroxyl group at position 2'' in P. aeruginosa, K. pneumoniae, Morganella morganii, E. coli, S. typhimurium, C. freundii and A. baumannii	ANT(2'')	aminoglycoside antibiotic	Aminoglycoside	Antibiotic inactivation	2	6	40	12	182	6	68	24	162	170	210	222	1104
ARO:3000237	TolC	TolC is a protein subunit of many multidrug efflux complexes in Gram negative bacteria. It is an outer membrane efflux protein and is constitutively open. Regulation of efflux activity is often at its periplasmic entrance by other components of the efflux complex.	ATP-binding cassette (ABC) antibiotic efflux pump;major facilitator superfamily (MFS) antibiotic efflux pump;resistance-nodulation-cell division (RND) antibiotic efflux pump	aminocoumarin antibiotic;aminoglycoside antibiotic;carbapenem;cephalosporin;cephamycin;fluoroquinolone antibiotic;glycylcycline;macrolide antibiotic;penam;penem;peptide antibiotic;phenicol antibiotic;rifamycin antibiotic;tetracycline antibiotic;triclosan	Multidrug	Antibiotic efflux	492	264	122	902	488	52	1466	1142	360	292	406	334	6320
ARO:3000245	RbpA	RNA-polymerase binding protein which confers resistance to rifampin.	RbpA bacterial RNA polymerase-binding protein	rifamycin antibiotic	Rifamycin	Antibiotic target protection	8	2	2	4	18	18	26	10	24	74	304	98	588
ARO:3000250	ErmC	ErmC is a methyltransferase that catalyzes the methylation of A2058 of the 23S ribosomal RNA in two steps. Expression of ErmC is inducible by erythromycin. The leader peptide causes	Erm 23S ribosomal RNA methyltransferase	lincosamide antibiotic;macrolide antibiotic;streptogramin antibiotic	MLS	Antibiotic target alteration	190	56	460	18	124	290	106	58	124	184	172	216	1998

		attenuation of the mRNA and stabilizes the structure preventing further translation. When erythromycin is present, it binds the leader peptide causing a change in conformation allowing for the expression of ErmC.																	
ARO:3000251	msrA	MsrA is an ABC-F subfamily ribosomal protection protein expressed in Staphylococcus species which confers resistance to erythromycin and streptogramin B antibiotics through antibiotic target protection mechanisms. It is associated with plasmid DNA.	ABC-F ATP-binding cassette ribosomal protection protein	lincosamide antibiotic;macrolide antibiotic;oxazolidinone antibiotic;phenicol antibiotic;pleuromutilin antibiotic;streptogramin antibiotic;tetracycline antibiotic	Multidrug	Antibiotic target protection	50	80	60	22	30	38	14	26	52	26	18	38	454
ARO:3000254	emrY	emrY is a multidrug transport that moves substrates across the inner membrane of the Gram-negative E. coli. It is a homolog of emrB.	major facilitator superfamily (MFS) antibiotic efflux pump	tetracycline antibiotic	Multidrug	Antibiotic efflux	892	372	268	1924	850	192	2650	2052	776	860	724	706	12266
ARO:3000263	marA	In the presence of antibiotic stress, E. coli overexpresses the global activator protein MarA, which besides inducing MDR efflux pump AcrAB, also down-regulates synthesis of the porin OmpF.	General Bacterial Porin with reduced permeability to beta-lactams;resistance-nodulation-cell division (RND) antibiotic efflux pump	carbapenem;cephalosporin;cephamycin;fluoroquinolone antibiotic;glycylcycline;monobactam;penam;penem;phenicol antibiotic;rifamycin antibiotic;tetracycline antibiotic;triclosan	Multidrug	Antibiotic efflux;reduced permeability to antibiotic	840	466	478	1464	778	262	1980	1628	634	272	318	144	9264
ARO:3000300	lsaA	LsaA is an ABC-F subfamily protein expressed in Enterococcus faecalis. It confers resistance to clindamycin, quinupristin-dalfopristin, and dalfopristin.	ABC-F ATP-binding cassette ribosomal protection protein	lincosamide antibiotic;macrolide antibiotic;oxazolidinone antibiotic;phenic	Multidrug	Antibiotic target protection	630	726	212	176	166	1302	210	214	162	244	186	152	4380

				ol antibiotic;pleuromutilin antibiotic;streptogramin antibiotic;tetracycline antibiotic														
ARO:3000316	mphA	The mphA gene encodes for resistance enzyme MPH(2')-I which preferentially inactivate 14-membered macrolides (e.g.erythromycin, telithromycin, roxithromycin) over 16-membered macrolides (e.g.tylosin, spiramycin). It phosphorylates macrolides at 2'-OH hydroxyl of desosamine sugar of macrolides in a GTP-dependent manner.	macrolide phosphotransferase (MPH)	macrolide antibiotic	MLS	Antibiotic inactivation	56	42	8	212	82	18	344	196	34	92	98	48	1230
ARO:3000318	mphB	The mphB gene encodes for MPH(2')-II. This enzymes phosphorylates 14-membered and 16-membered macrolides. It phosphorylates macrolides in GTP- dependent manner at 2'-OH hydroxyl of desosamine sugar of macrolides.	macrolide phosphotransferase (MPH)	macrolide antibiotic	MLS	Antibiotic inactivation	466	182	132	1076	396	32	1418	1132	326	128	140	44	5472
ARO:3000326	ErmE	ErmE is a methyltransferase found in the erythromycin producer Saccharopolyspora erythraea. Like other Erm enzymes, it catalyzes the methylation of A2058 of the 23S ribosomal RNA. The gene is found within the erythromycin biosynthetic cluster and is responsible for self-resistance.	Erm 23S ribosomal RNA methyltransferase	lincosamide antibiotic;macrolide antibiotic;streptogramin antibiotic	MLS	Antibiotic target alteration	748	1772	1390	400	412	1246	398	488	452	652	400	738	9096
ARO:3000343	tap	Efflux pump proteins contained within Mycobacterial genomes which confer resistance to a number of different antibiotics including aminoglycosides, and tetracyclines.	major facilitator superfamily (MFS) antibiotic efflux pump	tetracycline antibiotic	Multidrug	Antibiotic efflux	0	0	4	2	0	0	46	14	0	34	42	28	170
ARO:3000347	ErmA	ErmA confers the MLSb phenotype. Similar to ErmC, Expression of ErmA is inducible by erythromycin. The leader	Erm 23S ribosomal RNA	lincosamide antibiotic;macrolide	MLS	Antibiotic target alteration	102	66	60	26	56	104	28	24	56	798	994	1014	3328

		peptide causes attenuation of the mRNA and stabilizes the structure preventing further translation. When erythromycin is present, it binds the leader peptide causing a change in conformation allowing for the expression of ErmA.	methyltransferase	antibiotic;streptogramin antibiotic															
ARO:3000361	EreA	EreA is an erythromycin esterase that hydrolyses the drug's lactone ring.	macrolide esterase	macrolide antibiotic	MLS	Antibiotic inactivation	2	0	2	10	4	2	30	6	2	8	8	4	78
ARO:3000363	EreB	EreB is an erythromycin esterase-like protein that hydrolyses the drug's lactone ring.	macrolide esterase	macrolide antibiotic	MLS	Antibiotic inactivation	40	2	0	0	0	0	0	0	0	0	0	0	42
ARO:3000368	vanC	VanC is a D-Ala-D-Ala ligase homolog that synthesizes D-Ala-D-Ser, an alternative substrate for peptidoglycan synthesis that reduces vancomycin binding affinity. It is specific to Enterococcus gallinarum and E. casseliflavus, providing intrinsic resistance.	glycopeptide resistance gene cluster;van ligase	glycopeptide antibiotic	Glycopeptide	Antibiotic target alteration	8	160	100	26	22	52	38	84	48	80	138	164	920
ARO:3000375	ErmB	ErmB confers the MLSb phenotype. Similar to ErmC, expression of ErmB is inducible by erythromycin. The leader peptide causes attenuation of the mRNA and stabilizes the structure preventing further translation. When erythromycin is present, it binds the leader peptide causing a change in conformation allowing for the expression of ErmB.	Erm 23S ribosomal RNA methyltransferase	lincosamide antibiotic;macrolide antibiotic;streptogramin antibiotic	MLS	Antibiotic target alteration	1544	1196	534	424	170	1818	424	558	248	1666	1270	2316	12168
ARO:3000377	MexA	MexA is the membrane fusion protein of the MexAB-OprM multidrug efflux complex.	resistance-nodulation-cell division (RND) antibiotic efflux pump	aminocoumarin antibiotic;carbapenem;cephalosporin;cephamycin;diaminopyrimidine antibiotic;fluoroquinolone antibiotic;macrolide	Multidrug	Antibiotic efflux	306	260	1010	604	4954	280	862	1116	5076	3518	3682	4232	25900

				antibiotic;monobactam;penam;penem;peptide antibiotic;phenicol antibiotic;sulfonamide antibiotic;tetracycline antibiotic															
ARO:3000378	MexB	MexB is the inner membrane multidrug exporter of the efflux complex MexAB-OprM.	resistance-nodulation-cell division (RND) antibiotic efflux pump	aminocoumarin antibiotic;carbapenem;cephalosporin;cephamycin;diaminopyrimidine antibiotic;fluoroquinolone antibiotic;macrolide antibiotic;monobactam;penam;penem;peptide antibiotic;phenicol antibiotic;sulfonamide antibiotic;tetracycline antibiotic	Multidrug	Antibiotic efflux	1774	2104	1582	1212	5378	1756	1638	1816	5702	4262	3914	4780	35918
ARO:3000379	OprM	OprM is an outer membrane factor protein found in Pseudomonas aeruginosa and Burkholderia vietnamiensis. It is part of the MexAB-OprM, MexVW-OprM, MexXY-OprM and the AmrAB-OprM complex.	resistance-nodulation-cell division (RND) antibiotic efflux pump	acridine dye;aminocoumarin antibiotic;aminoglycoside antibiotic;carbapenem;cephalosporin;cephamycin;diaminopyrimidine antibiotic;fluoroquinolone antibiotic;macrolide antibiotic;monobactam;penam;penem;peptide antibiotic;phenicol	Multidrug	Antibiotic efflux	690	978	1978	1202	4872	784	1672	1554	5142	4250	4064	5306	32492

				antibiotic;sulfonamide antibiotic;tetracycline antibiotic															
ARO:3000391	norA	NorA is a multidrug efflux pump in Staphylococcus aureus that confers resistance to fluoroquinolones and other structurally unrelated antibiotics like acriflavine. It shares 30% similarity with NorA, and is a structural homolog of Bmr of Bacillus subtilis. It is regulated by arlRS and mgrA, the latter also known as NorR.	major facilitator superfamily (MFS) antibiotic efflux pump	acridine dye;fluoroquinolone antibiotic	Multidrug	Antibiotic efflux	2	34	4	30	10	14	10	60	0	0	0	0	164
ARO:3000392	Erm(37)	Erm(37) is found in Mycobacterium species and confers the MLSb phenotype. In addition to methylation of A2058 this Erm methylates adjacent adenosines (A2057 and A2059) as well.	Erm 23S ribosomal RNA methyltransferase	lincosamide antibiotic;macrolide antibiotic;streptogramin antibiotic	MLS	Antibiotic target alteration	0	0	6	0	0	0	0	0	0	92	180	156	434
ARO:3000410	sul1	Sul1 is a sulfonamide resistant dihydropteroate synthase of Gram-negative bacteria. It is linked to other resistance genes of class 1 integrons.	sulfonamide resistant sul	sulfonamide antibiotic;sulfone antibiotic	Sulfonamide	Antibiotic target replacement	290	1006	1370	1320	5190	322	2432	2192	4818	10550	11592	15170	56252
ARO:3000412	sul2	Sul2 is a sulfonamide resistant dihydropteroate synthase of Gram-negative bacteria, usually found on small plasmids.	sulfonamide resistant sul	sulfonamide antibiotic;sulfone antibiotic	Sulfonamide	Antibiotic target replacement	2290	4526	1866	3326	9054	1860	3264	4332	8092	13988	13790	18046	84434
ARO:3000413	sul3	Sul3 is a sulfonamide resistant dihydropteroate synthase similar to Sul1 and Sul2. Its resistance gene was found encoded in E. coli plasmid DNA of sulfonamide resistant isolates.	sulfonamide resistant sul	sulfonamide antibiotic;sulfone antibiotic	Sulfonamide	Antibiotic target replacement	206	638	524	728	392	278	1144	790	436	224	222	170	5752
ARO:3000421	norB	NorB is a multidrug efflux pump in Staphylococcus aureus that confers resistance to fluoroquinolones and other structurally unrelated antibiotics like tetracycline. It shares 30% similarity with NorB, and is a structural homolog of Blt of	major facilitator superfamily (MFS) antibiotic efflux pump	fluoroquinolone antibiotic	Multidrug	Antibiotic efflux	22	180	62	4	84	174	30	6	52	234	250	376	1474

		Bacillus subtilis. It is regulated by mgrA, also known as NorR.																
ARO:3000444	rphA	The enzymatic inactivation of rifampin by phosphorylation at the 21-OH position.	rifampin phosphotransferase	rifamycin antibiotic	Rifamycin	Antibiotic inactivation	86	72	158	262	384	200	402	342	416	926	900	1148	5296
ARO:3000448	QepA1	QepA1 is a plasmid-mediated efflux pump in E. coli, shown to contribute to fluoroquinolone resistance. It is regulated by sox genes, also known as global stress regulators.	major facilitator superfamily (MFS) antibiotic efflux pump	fluoroquinolone antibiotic	Fluoroquinolone	Antibiotic efflux	2	0	4	10	2	0	46	22	0	450	418	598	1552
ARO:3000462	mgtA	A macrolide glycosyltransferase encoded by the mgtA gene in Streptomyces lividans. This enzyme inactivates macrolides using UDP-glucose as a cofactor. Its optimal substrates are lankamycin, calcomycin, rosaramicin, methymycin, and pikromycin, while interactions with erythomycin, oldeandomycin, azithromycin, and tylosin were weaker. It is inactive against spiramycin and carbomycin. Mechanism first described by Cundliffe, 1992.	mgt macrolide glycotransferase	macrolide antibiotic	MLS	Antibiotic inactivation	0	0	0	0	0	0	0	0	0	4	2	0	6
ARO:3000463	gimA	A macrolide glycosyltransferase encoded by the gimA gene in Streptomyces ambofaciens, a natural producer of the macrolide antibiotic spiramycin. Chalcomycin, methymycin, tylosin, pikromycin, rosaramicin, oleandomycin, josamycin, and carbomycin are preferred substrates of gimA glycosyltransferase, while erythromycin and spiramycin have notably low binding affinities. GimA may be able to inactivate spiramycin precursors. Described by Gourmelen et al. 1998.	gimA family macrolide glycosyltransferase	macrolide antibiotic	MLS	Antibiotic inactivation	194	266	176	152	122	238	194	186	176	22	42	16	1784
ARO:3000476	tet(31)	Tet31 is a tetracycline efflux pump found in Aeromonas salmonicida, a Gram-negative	major facilitator superfamily	tetracycline antibiotic	Tetracycline	Antibiotic efflux	0	2	10	34	84	0	90	116	124	92	140	94	786

		bacteria. It has also been shown to be expressed in Gallibacterium anatis.	(MFS) antibiotic efflux pump																
ARO:3000478	tet(33)	Tet33 is a tetracycline efflux pump found in Gram-positive bacteria, including Arthrobacter and Corynebacterium.	major facilitator superfamily (MFS) antibiotic efflux pump	tetracycline antibiotic	Tetracycline	Antibiotic efflux	36	20	26	62	30	82	104	42	96	234	414	198	1344
ARO:3000481	tet(35)	Tet35 is a tetracycline efflux pump found in the Gram-negative Vibrio and Stenotrophomonas. It is unrelated to other tet resistance genes.	ATP-binding cassette (ABC) antibiotic efflux pump	tetracycline antibiotic	Tetracycline	Antibiotic efflux	2630	4790	3182	1460	1230	3932	1646	1798	1398	4672	3758	6594	37090
ARO:3000491	acrD	AcrD is an aminoglycoside efflux pump expressed in E. coli. Its expression can be induced by indole, and is regulated by baeRS and cpxAR.	resistance-nodulation-cell division (RND) antibiotic efflux pump	aminoglycoside antibiotic	Aminoglycoside	Antibiotic efflux	620	354	266	1132	590	174	2074	1354	472	422	576	398	8432
ARO:3000495	ErmD	ErmD confers MLSb phenotype.	Erm 23S ribosomal RNA methyltransferase	lincosamide antibiotic;macrolide antibiotic;streptogramin antibiotic	MLS	Antibiotic target alteration	212	180	258	144	72	184	110	166	72	114	80	88	1680
ARO:3000498	ErmF	ErmF confers the MLSb phenotype.	Erm 23S ribosomal RNA methyltransferase	lincosamide antibiotic;macrolide antibiotic;streptogramin antibiotic	MLS	Antibiotic target alteration	1694	2040	2908	1122	800	2006	964	1542	1142	2130	1830	2128	20306
ARO:3000499	AcrE	AcrE is a membrane fusion protein, similar to AcrA.	resistance-nodulation-cell division (RND) antibiotic efflux pump	cephalosporin;cephamycin;fluoroquinolone antibiotic;penam	Multidrug	Antibiotic efflux	862	200	288	1194	652	246	1606	1140	512	188	190	36	7114
ARO:3000501	rpoB2	Due to gene duplication, the genomes of Nocardia species include both rifampin-sensitive beta-subunit of RNA polymerase (rpoB) and rifampin-resistant beta-subunit of RNA polymerase (rpoB2) genes, with ~88% similarity between the two gene products. Expression of the rpoB2 variant results in	rifamycin-resistant beta-subunit of RNA polymerase (rpoB)	peptide antibiotic;rifamycin antibiotic	Multidrug	Antibiotic target alteration;antibiotic target replacement	7536	10658	8100	5790	3284	9102	6540	6986	4430	7704	8110	8140	86380

		replacement of rifampin sensitivity with rifampin resistance.																	
ARO:3000502	AcrF	AcrF is a inner membrane transporter, similar to AcrB.	resistance-nodulation-cell division (RND) antibiotic efflux pump	cephalosporin;cephamycin;fluoroquinolone antibiotic;penam	Multidrug	Antibiotic efflux	640	264	240	1144	524	138	1784	1408	476	278	310	116	7322
ARO:3000504	golS	GolS is a regulator activated by the presence of golD, and promotes the expression of the MdsABC efflux pump.	resistance-nodulation-cell division (RND) antibiotic efflux pump	carbapenem;cephalosporin;cephamycin;monobactam;penam;penem;phenicol antibiotic	Multidrug	Antibiotic efflux	1574	1354	1160	1882	4050	1460	2712	2792	3750	3064	3288	3782	30868
ARO:3000508	gadX	GadX is an AraC-family regulator that promotes mdtEF expression to confer multidrug resistance.	resistance-nodulation-cell division (RND) antibiotic efflux pump	fluoroquinolone antibiotic;macrolide antibiotic;penam	Multidrug	Antibiotic efflux	1210	452	408	2216	1156	200	3330	2650	872	362	414	306	13576
ARO:3000510	Staphylococcus mupB conferring resistance to mupirocin	An alternative isoleucyl-tRNA synthetase conferring resistance to mupirocin.	antibiotic-resistant isoleucyl-tRNA synthetase (ileS)	mupirocin	Mupirocin	Antibiotic target alteration	4940	8568	7036	3006	4442	6800	3480	3856	4848	4944	4406	4434	60760
ARO:3000516	emrR	EmrR is a negative regulator for the EmrAB-TolC multidrug efflux pump in E. coli. Mutations lead to EmrAB-TolC overexpression.	major facilitator superfamily (MFS) antibiotic efflux pump	fluoroquinolone antibiotic	Multidrug	Antibiotic efflux	326	152	106	608	320	62	964	752	224	120	108	112	3854
ARO:3000518	CRP	CRP is a global regulator that represses MdtEF multidrug efflux pump expression.	resistance-nodulation-cell division (RND) antibiotic efflux pump	fluoroquinolone antibiotic;macrolide antibiotic;penam	Multidrug	Antibiotic efflux	694	738	538	914	1476	520	1276	1060	1218	1700	1562	1946	13642
ARO:3000521	Staphylococcus mupA conferring	An alternative isoleucyl-tRNA synthetase conferring resistance to mupirocin.	antibiotic-resistant isoleucyl-tRNA	mupirocin	Mupirocin	Antibiotic target alteration	10090	11988	8818	6642	3906	10866	8402	7442	4916	10114	8276	11532	102992

	resistance to mupirocin		synthetase (ileS)																
ARO:3000522	ErmG	ErmG is a rRNA adenine N-6-methyltransferase that protects the ribosome from inactivation due to antibiotic binding.	Erm 23S ribosomal RNA methyltransferase	lincosamide antibiotic;macrolide antibiotic;streptogramin antibiotic	MLS	Antibiotic target alteration	308	730	492	94	52	590	132	114	118	98	84	40	2852
ARO:3000526	cmeR	CmeR is a repressor for the CmeABC multidrug efflux pump, binding to the cmeABC promoter region.	resistance-nodulation-cell division (RND) antibiotic efflux pump	cephalosporin;fluoroquinolone antibiotic;fusidic acid;macrolide antibiotic	Multidrug	Antibiotic efflux	16	18	10	10	8	14	18	12	6	34	126	146	418
ARO:3000533	macA	MacA is a membrane fusion protein that forms an antibiotic efflux complex with MacB and TolC. macA corresponds to 1 locus in Pseudomonas aeruginosa PAO1 and 1 locus in Pseudomonas aeruginosa LESB58.	ATP-binding cassette (ABC) antibiotic efflux pump	macrolide antibiotic	MLS	Antibiotic efflux	2162	2388	1396	2010	1546	2036	2274	2014	1812	3826	3772	5452	30688
ARO:3000535	macB	MacB is an ATP-binding cassette (ABC) transporter that exports macrolides with 14- or 15-membered lactones. It forms an antibiotic efflux complex with MacA and TolC. macB corresponds to 1 locus in Pseudomonas aeruginosa PAO1 and 1 locus in Pseudomonas aeruginosa LESB58.	ATP-binding cassette (ABC) antibiotic efflux pump	macrolide antibiotic	MLS	Antibiotic efflux	48978	48486	42436	36076	32332	48462	46500	41274	35282	38412	37434	42408	498080
ARO:3000549	adeS	AdeS is a sensor kinase in the AdeRS regulatory system of AdeABC. It is essential for AdeABC expression.	resistance-nodulation-cell division (RND) antibiotic efflux pump	glycylcycline;tetracycline antibiotic	Multidrug	Antibiotic efflux	1042	1696	1210	1382	892	972	1346	1618	760	1718	1612	2074	16322
ARO:3000553	adeR	AdeR is a positive regulator of AdeABC efflux system. AdeR inactivation leads to susceptibility to aminoglycoside antibiotics.	resistance-nodulation-cell division (RND) antibiotic efflux pump	glycylcycline;tetracycline antibiotic	Multidrug	Antibiotic efflux	1232	1220	1022	838	552	1148	908	966	916	2860	1942	3392	16996
ARO:3000556	tet(44)	Tet44 is a tetracycline resistance gene found in Campylobacter	tetracycline-resistant	tetracycline antibiotic	Tetracycline	Antibiotic target	2028	2718	1130	966	446	2302	742	994	614	1608	1394	1934	16876

		fetus, and binds to the ribosome to confer antibiotic resistance as a ribosomal protection protein.	ribosomal protection protein			protecti on													
ARO:3000 559	adeN	AdeN is a repressor of AdeIJK, a RND-type efflux pump in Acinetobacter baumannii. Its inactivation increases expression of AdeJ.	resistance-nodulation-cell division (RND) antibiotic efflux pump	carbapenem;cep halosporin;diami nopyrimidine antibiotic;fluoro quinolone antibiotic;lincosa mide antibiotic;macrol ide antibiotic;penem ;phenicol antibiotic;rifamy cin antibiotic;tetracy cline antibiotic	Multidrug	Antibioti c efflux	608	1038	50	148	114	572	182	188	124	428	310	486	4248
ARO:3000 561	tet(30)	Tet30 is a tetracycline efflux pump found in agrobacterium, a Gram-negative bacterium.	major facilitator superfamily (MFS) antibiotic efflux pump	tetracycline antibiotic	Tetracyclin e	Antibioti c efflux	0	0	36	0	12	0	0	2	10	16	2	0	78
ARO:3000 565	tet(38)	Tet38 is a tetracycline efflux pump found in the Gram-positive Staphylococcus aureus. It is regulated by mgrA, which also regulates NorB.	major facilitator superfamily (MFS) antibiotic efflux pump	tetracycline antibiotic	Tetracyclin e	Antibioti c efflux	6	4	6	4	16	8	52	10	22	164	330	120	742
ARO:3000 566	tet(39)	Tet39 is a tetracycline efflux pump found in Gram-negative bacteria, including Brevundimonas, Stenotrophomonas, Enterobacter, Alcaligenes, Acinetobacter, and Providencia.	major facilitator superfamily (MFS) antibiotic efflux pump	tetracycline antibiotic	Tetracyclin e	Antibioti c efflux	90	340	22	38	70	160	40	102	86	126	84	118	1276
ARO:3000 567	tet(40)	Tet40 is a tetracycline efflux pump found in the Gram-positive Clostridium. It is similar to tetA(P).	major facilitator superfamily (MFS) antibiotic efflux pump	tetracycline antibiotic	Tetracyclin e	Antibioti c efflux	2732	2662	2908	1172	1184	2796	1220	1244	1662	482	266	206	18534
ARO:3000 569	tet(41)	Tet41 is a tetracycline efflux pump found in Serratia, a Gram-negative bacterium. It is related to Acinetobacter Tet(39).	major facilitator superfamily (MFS)	tetracycline antibiotic	Tetracyclin e	Antibioti c efflux	0	96	36	6	66	40	10	6	18	234	258	266	1036

ARO:3000572	tet(42)	Tet42 is a tetracycline efflux pump found in both Gram-negative (Pseudomonas) and Gram-positive (Microbacterium, Bacillus, Staphylococcus, Paenibacillus) bacteria.	antibiotic efflux pump major facilitator superfamily (MFS) antibiotic efflux pump	tetracycline antibiotic	Tetracycline	Antibiotic efflux	10	8	8	14	40	16	38	22	80	328	618	422	1604
ARO:3000573	tet(43)	Tet(43) is a tetracycline resistance gene with unknown origins, isolated from metagenomic DNA.	major facilitator superfamily (MFS) antibiotic efflux pump	tetracycline antibiotic	Tetracycline	Antibiotic efflux	0	0	0	4	20	4	30	2	10	40	74	16	200
ARO:3000582	L1 beta-lactamase	L1 is an Ambler class B MBL; subclass B3 originally isolated from Stenotrophomonas maltophilia. It has activity against a broad range of beta-lactams and is only active with two Zn(II) ions in the active site.	L1 family beta-lactamase	cephalosporin	Beta-lactam	Antibiotic inactivation	24	6	4	0	0	8	0	0	0	2	10	6	60
ARO:3000589	NDM-1	NDM-1 is a metallo-beta-lactamase isolated from Klebsiella pneumoniae with nearly complete resistance to all beta-lactam antibiotics.	NDM beta-lactamase	carbapenem;cephalosporin;cephamycin;penam	Beta-lactam	Antibiotic inactivation	0	0	2	2	184	0	0	0	180	14	32	8	422
ARO:3000592	ErmN	ErmN is a methyltransferase found in the tylosin producer Streptomyces fradiae. Like other Erm enzymes, it catalyzes the methylation of A2058 of the 23S ribosomal RNA. Specifically, this enzyme transfers only one methyl group. The gene is found in the tylosin biosynthetic cluster and is responsible for self-resistance to tylosin.	Erm 23S ribosomal RNA methyltransferase	lincosamide antibiotic;macrolide antibiotic;streptogramin antibiotic	MLS	Antibiotic target alteration	314	12	46	8	0	134	4	12	0	202	314	248	1294
ARO:3000593	ErmQ	ErmQ confers MLSb phenotype.	Erm 23S ribosomal RNA methyltransferase	lincosamide antibiotic;macrolide antibiotic;streptogramin antibiotic	MLS	Antibiotic target alteration	42	180	230	248	64	110	180	210	60	86	90	38	1538
ARO:3000594	ErmR	ErmR is a methyltransferase found in the erythromycin producer Aeromicrobium erythreum. Like other Erm enzymes, it catalyzes the	Erm 23S ribosomal RNA methyltransferase	lincosamide antibiotic;macrolide antibiotic;streptogramin antibiotic	MLS	Antibiotic target alteration	42	588	172	68	42	312	44	66	76	14	8	10	1442

		methylation of A2058 of the 23S ribosomal RNA. The gene is found within the erythromycin biosynthetic cluster and is responsible for self-resistance.																	
ARO:3000595	ErmT	ErmT confers MLSb phenotype.	Erm 23S ribosomal RNA methyltransferase	lincosamide antibiotic;macrolide antibiotic;streptogramin antibiotic	MLS	Antibiotic target alteration	100	116	72	22	32	74	12	2	50	392	382	346	1600
ARO:3000596	ErmX	ErmX is a rRNA methyltransferase that protects the ribosome from inactivation due to antibiotic binding.	Erm 23S ribosomal RNA methyltransferase	lincosamide antibiotic;macrolide antibiotic;streptogramin antibiotic	MLS	Antibiotic target alteration	24	28	30	38	104	96	120	46	228	394	744	514	2366
ARO:3000598	Erm(31)	Erm(31) confers a MLSb resistant phenotype. Along with erm(30), these genes are responsible for self-resistance in the pikromycin/narbomycin/methymycin/neomethymycin producer, Streptomyces venezuelae.	Erm 23S ribosomal RNA methyltransferase	lincosamide antibiotic;macrolide antibiotic;streptogramin antibiotic	MLS	Antibiotic target alteration	14	0	16	22	48	26	94	22	52	242	394	218	1148
ARO:3000600	Erm(34)	ErmD confers MLSb phenotype.	Erm 23S ribosomal RNA methyltransferase	lincosamide antibiotic;macrolide antibiotic;streptogramin antibiotic	MLS	Antibiotic target alteration	776	490	684	1702	2134	316	1976	1862	1870	1474	1382	1760	16426
ARO:3000601	Erm(38)	ErmD confers MLSb phenotype.	Erm 23S ribosomal RNA methyltransferase	lincosamide antibiotic;macrolide antibiotic;streptogramin antibiotic	MLS	Antibiotic target alteration	0	12	8	40	10	0	56	68	10	22	26	6	258
ARO:3000604	Erm(35)	ErmD confers MLSb phenotype.	Erm 23S ribosomal RNA methyltransferase	lincosamide antibiotic;macrolide antibiotic;streptogramin antibiotic	MLS	Antibiotic target alteration	36	82	176	80	14	54	32	74	18	546	440	834	2386
ARO:3000605	Erm(36)	ErmD confers MLSb phenotype.	Erm 23S ribosomal RNA methyltransferase	lincosamide antibiotic;macrolide antibiotic;streptogramin antibiotic	MLS	Antibiotic target alteration	6	4	24	22	4	12	58	22	14	142	276	168	752
ARO:3000606	FEZ-1	FEZ-1 is an Ambler class B MBL; subclass B3 first isolated from Legionella gormanii. It has	FEZ beta-lactamase	carbapenem;cephalosporin;penam	Beta-lactam	Antibiotic	4	22	6	4	10	4	0	2	8	0	0	0	60

		activity against a broad range of beta-lactams and is only active with two Zn(II) ions in the active site.				inactivation													
ARO:3000614	mefE	mefE is a proton motive efflux pump in Streptococcus pneumoniae that confers resistance to macrolides. It is found on the same operon as mefA and the ABC-efflux pump mel.	major facilitator superfamily (MFS) antibiotic efflux pump	macrolide antibiotic	MLS	Antibiotic efflux	286	32	200	34	162	394	42	54	172	52	66	92	1586
ARO:3000616	mel	Mel, a homolog of MsrA, is an ABC-F subfamily protein associated with macrolide resistance. It is expressed on the same operon as mefA and mefE, both MFS-type efflux proteins that confer macrolide resistance.	ABC-F ATP-binding cassette ribosomal protection protein	lincosamide antibiotic;macrolide antibiotic;oxazolidinone antibiotic;phenicol antibiotic;pleuromutilin antibiotic;streptogramin antibiotic;tetracycline antibiotic	Multidrug	Antibiotic target protection	4028	5566	6072	3408	2070	4882	2706	3624	2598	1902	1402	1196	39454
ARO:3000617	mecA	A foreign PBP2a acquired by lateral gene transfer that able to perform peptidoglycan synthesis in the presence of beta-lactams.	methicillin resistant PBP2	carbapenem;cephalosporin;cephamycin;monobactam;penam	Beta-lactam	Antibiotic target replacement	190	640	830	516	480	428	568	632	620	340	324	358	5926
ARO:3000620	adeL	AdeL is a regulator of AdeFGH in Acinetobacter baumannii. AdeL mutations are associated with AdeFGH overexpression and multidrug resistance.	resistance-nodulation-cell division (RND) antibiotic efflux pump	fluoroquinolone antibiotic;tetracycline antibiotic	Multidrug	Antibiotic efflux	4072	3702	2978	6934	6278	2018	9760	7944	5272	8650	8456	11584	77648
ARO:3000656	AcrS	AcrS is a repressor of the AcrAB efflux complex and is associated with the expression of AcrEF. AcrS is believed to regulate a switch between AcrAB and AcrEF efflux.	resistance-nodulation-cell division (RND) antibiotic efflux pump	cephalosporin;cephamycin;fluoroquinolone antibiotic;glycylcycline;penam;phenicol antibiotic;rifamycin antibiotic;tetracycline antibiotic;triclosan	Multidrug	Antibiotic efflux	958	496	1306	1930	4778	436	3148	2872	4330	2602	2674	2570	28100

ARO:3000676	H-NS	H-NS is a histone-like protein involved in global gene regulation in Gram-negative bacteria. It is a repressor of the membrane fusion protein genes acrE, mdtE, and emrK as well as nearby genes of many RND-type multidrug exporters.	major facilitator superfamily (MFS) antibiotic efflux pump;resistance-nodulation-cell division (RND) antibiotic efflux pump	cephalosporin;cephamycin;fluoroquinolone antibiotic;macrolide antibiotic;penam;tetracycline antibiotic	Multidrug	Antibiotic efflux	468	196	100	818	506	40	1312	896	396	118	164	76	5090
ARO:3000746	mepR	MepR is an upstream repressor of MepA in Staphylococcus aureus. It is part of the mepRAB operon.	multidrug and toxic compound extrusion (MATE) transporter	glycylcycline;tetracycline antibiotic	Multidrug	Antibiotic efflux	56	36	224	14	24	94	42	34	36	48	24	14	646
ARO:3000753	abeM	AbeM is an multidrug efflux pump found in Acinetobacter baumannii.	multidrug and toxic compound extrusion (MATE) transporter	acridine dye;fluoroquinolone antibiotic;triclosan	Multidrug	Antibiotic efflux	714	1916	420	248	412	986	320	294	326	666	546	1000	7848
ARO:3000768	abeS	AbeS in an efflux pump of the SMR family of transporters found in Acinetobacter baumannii.	small multidrug resistance (SMR) antibiotic efflux pump	aminocoumarin antibiotic;macrolide antibiotic	Multidrug	Antibiotic efflux	8	46	148	34	72	78	120	44	50	192	236	284	1312
ARO:3000774	adeA	AdeA is the membrane fusion protein of the multidrug efflux complex AdeABC.	resistance-nodulation-cell division (RND) antibiotic efflux pump	glycylcycline;tetracycline antibiotic	Multidrug	Antibiotic efflux	424	678	1014	128	222	326	118	236	294	410	454	472	4776
ARO:3000775	adeB	AdeB is the multidrug transporter of the adeABC efflux system.	resistance-nodulation-cell division (RND) antibiotic efflux pump	glycylcycline;tetracycline antibiotic	Multidrug	Antibiotic efflux	184	148	132	270	82	154	256	320	90	2166	2116	3288	9206
ARO:3000777	adeF	AdeF is the membrane fusion protein of the multidrug efflux complex AdeFGH.	resistance-nodulation-cell division (RND)	fluoroquinolone antibiotic;tetracycline antibiotic	Multidrug	Antibiotic efflux	542	1520	2162	1426	1036	780	1200	1532	1322	1068	668	660	13916

			antibiotic efflux pump																
ARO:3000778	adeG	AdeG is the inner membrane transporter of the AdeFGH multidrug efflux complex.	resistance-nodulation-cell division (RND) antibiotic efflux pump	fluoroquinolone antibiotic;tetracycline antibiotic	Multidrug	Antibiotic efflux	1458	616	586	1082	534	652	1546	1290	536	2568	2938	3918	17724
ARO:3000779	adeH	AdeH is the outer membrane channel protein of the AdeFGH multidrug efflux complex.	resistance-nodulation-cell division (RND) antibiotic efflux pump	fluoroquinolone antibiotic;tetracycline antibiotic	Multidrug	Antibiotic efflux	776	2156	1974	1336	856	1082	796	1170	964	1118	598	858	13684
ARO:3000780	adeI	AdeI is the membrane fusion protein of the AdeIJK multidrug efflux complex.	resistance-nodulation-cell division (RND) antibiotic efflux pump	carbapenem;cephalosporin;diaminopyrimidine antibiotic;fluoroquinolone antibiotic;lincosamide antibiotic;macrolide antibiotic;penem;phenicol antibiotic;rifamycin antibiotic;tetracycline antibiotic	Multidrug	Antibiotic efflux	906	1768	324	334	258	1126	314	388	238	306	204	478	6644
ARO:3000781	adeJ	AdeJ is a RND efflux protein that acts as the inner membrane transporter of the AdeIJK efflux complex. It has 57% identity with E. coli AcrB.	resistance-nodulation-cell division (RND) antibiotic efflux pump	carbapenem;cephalosporin;diaminopyrimidine antibiotic;fluoroquinolone antibiotic;lincosamide antibiotic;macrolide antibiotic;penem;phenicol antibiotic;rifamycin antibiotic;tetracycline antibiotic	Multidrug	Antibiotic efflux	988	2594	344	448	316	1498	386	450	332	412	372	754	8894
ARO:3000782	adeK	AdeK is the outer membrane factor protein in the adeIJK multidrug efflux complex.	resistance-nodulation-cell division	carbapenem;cephalosporin;diaminopyrimidine	Multidrug	Antibiotic efflux	1082	2728	1178	1026	670	1720	870	1156	666	1394	1210	1796	15496

			(RND) antibiotic efflux pump	antibiotic;fluoroquinolone antibiotic;lincosamide antibiotic;macrolide antibiotic;penem;phenicol antibiotic;rifamycin antibiotic;tetracycline antibiotic															
ARO:3000783	cmeA	CmeA is the membrane fusion protein of the CmeABC multidrug efflux complex.	resistance-nodulation-cell division (RND) antibiotic efflux pump	cephalosporin;fluoroquinolone antibiotic;fusidic acid;macrolide antibiotic	Multidrug	Antibiotic efflux	242	270	356	174	198	292	128	170	256	428	356	706	3576
ARO:3000784	cmeB	CmeB is the inner membrane transporter the CmeABC multidrug efflux complex.	resistance-nodulation-cell division (RND) antibiotic efflux pump	cephalosporin;fluoroquinolone antibiotic;fusidic acid;macrolide antibiotic	Multidrug	Antibiotic efflux	918	1682	1690	942	368	1484	790	976	578	2816	2906	4004	19154
ARO:3000785	cmeC	CmeC is the outer membrane channel protein of the CmeABC multidrug efflux complex.	resistance-nodulation-cell division (RND) antibiotic efflux pump	cephalosporin;fluoroquinolone antibiotic;fusidic acid;macrolide antibiotic	Multidrug	Antibiotic efflux	22	48	32	18	16	24	30	16	20	1322	1142	2230	4920
ARO:3000789	mdsA	MdsA is the membrane fusion protein of the multidrug and metal efflux complex MdsABC.	resistance-nodulation-cell division (RND) antibiotic efflux pump	carbapenem;cephalosporin;cephamycin;monobactam;penam;penem;phenicol antibiotic	Multidrug	Antibiotic efflux	12	0	80	78	28	20	28	32	14	28	60	12	392
ARO:3000790	mdsB	MdsB is the inner membrane transporter of the multidrug and metal efflux complex MdsABC. mdsB corresponds to 1 locus in Pseudomonas aeruginosa PAO1 (gene name: mexQ) and 2 loci in Pseudomonas aeruginosa LESB58.	resistance-nodulation-cell division (RND) antibiotic efflux pump	carbapenem;cephalosporin;cephamycin;monobactam;penam;penem;phenicol antibiotic	Multidrug	Antibiotic efflux	98	258	386	78	196	196	146	130	158	590	746	836	3818
ARO:3000791	mdsC	MdsC is the outer membrane channel of the multidrug and metal efflux complex MdsABC.	resistance-nodulation-cell division	carbapenem;cephalosporin;cephamycin;monobact	Multidrug	Antibiotic efflux	134	186	168	148	46	112	176	172	92	56	100	62	1452

			(RND) antibiotic efflux pump	am;penam;penem;phenicol antibiotic																
ARO:3000792	mdtA	MdtA is the membrane fusion protein of the multidrug efflux complex mdtABC.	resistance-nodulation-cell division (RND) antibiotic efflux pump	aminocoumarin antibiotic	Multidrug	Antibiotic efflux	750	1026	542	1140	678	732	1492	1814	658	900	1150	1138	12020
ARO:3000793	mdtB	MdtB is a transporter that forms a heteromultimer complex with MdtC to form a multidrug transporter. MdtBC is part of the MdtABC-TolC efflux complex.	resistance-nodulation-cell division (RND) antibiotic efflux pump	aminocoumarin antibiotic	Multidrug	Antibiotic efflux	1846	1658	1548	1904	1252	1630	2566	2266	1420	7374	6156	10672	40292
ARO:3000794	mdtC	MdtC is a transporter that forms a heteromultimer complex with MdtB to form a multidrug transporter. MdtBC is part of the MdtABC-TolC efflux complex. In the absence of MdtB, MdtC can form a homomultimer complex that results in a functioning efflux complex with a narrower drug specificity. mdtC corresponds to 3 loci in Pseudomonas aeruginosa PAO1 (gene name: muxC/muxB) and 3 loci in Pseudomonas aeruginosa LESB58.	resistance-nodulation-cell division (RND) antibiotic efflux pump	aminocoumarin antibiotic	Multidrug	Antibiotic efflux	2874	3070	4014	3380	7980	2006	4538	4828	7710	4564	4006	4552	53522
ARO:3000795	mdtE	MdtE is the membrane fusion protein of the MdtEF multidrug efflux complex. It shares 70% sequence similarity with AcrA.	resistance-nodulation-cell division (RND) antibiotic efflux pump	fluoroquinolone antibiotic;macrolide antibiotic;penam	Multidrug	Antibiotic efflux	522	284	332	1286	606	158	1782	1320	586	386	368	324	7954
ARO:3000796	mdtF	MdtF is the multidrug inner membrane transporter for the MdtEF-TolC efflux complex.	resistance-nodulation-cell division (RND) antibiotic efflux pump	fluoroquinolone antibiotic;macrolide antibiotic;penam	Multidrug	Antibiotic efflux	740	466	226	1260	640	258	1924	1550	586	446	282	248	8626
ARO:3000800	MexC	MexC is the membrane fusion protein of the MexCD-OprJ multidrug efflux complex.	resistance-nodulation-cell division (RND)	aminocoumarin antibiotic;aminoglycoside antibiotic;cephal	Multidrug	Antibiotic efflux	102	186	1504	518	5738	176	912	1438	5328	1914	2244	2534	22594

			antibiotic efflux pump	osporin;diaminopyrimidine antibiotic;fluoroquinolone antibiotic;macrolide antibiotic;penam;phenicol antibiotic;tetracycline antibiotic															
ARO:3000801	MexD	MexD is the multidrug inner membrane transporter of the MexCD-OprJ complex.	resistance-nodulation-cell division (RND) antibiotic efflux pump	aminocoumarin antibiotic;aminoglycoside antibiotic;cephalosporin;diaminopyrimidine antibiotic;fluoroquinolone antibiotic;macrolide antibiotic;penam;phenicol antibiotic;tetracycline antibiotic	Multidrug	Antibiotic efflux	312	358	3528	2142	17074	270	3066	5504	15418	5610	6216	7364	66862
ARO:3000802	OprJ	OprJ is the outer membrane channel component of the MexCD-OprJ multidrug efflux complex.	resistance-nodulation-cell division (RND) antibiotic efflux pump	aminocoumarin antibiotic;aminoglycoside antibiotic;cephalosporin;diaminopyrimidine antibiotic;fluoroquinolone antibiotic;macrolide antibiotic;penam;phenicol antibiotic;tetracycline antibiotic	Multidrug	Antibiotic efflux	62	122	1354	826	7020	46	1308	1812	6268	1910	2204	2256	25188
ARO:3000803	MexE	MexE is the membrane fusion protein of the MexEF-OprN multidrug efflux complex.	resistance-nodulation-cell division (RND) antibiotic efflux pump	diaminopyrimidine antibiotic;fluoroquinolone antibiotic;phenicol antibiotic	Multidrug	Antibiotic efflux	222	24	44	26	14	154	18	70	40	492	498	458	2060
ARO:3000804	MexF	MexF is the multidrug inner membrane transporter of the MexEF-OprN complex. mexF	resistance-nodulation-cell division	diaminopyrimidine antibiotic;fluoro	Multidrug	Antibiotic efflux	368	192	262	216	82	356	208	224	146	314	388	450	3206

		corresponds to 2 loci in Pseudomonas aeruginosa PAO1 (gene name: mexF/mexB) and 4 loci in Pseudomonas aeruginosa LESB58 (gene name: mexD/mexB).	(RND) antibiotic efflux pump	quinolone antibiotic;phenicol antibiotic															
ARO:3000805	OprN	OprN is the outer membrane channel component of the MexEF-OprN multidrug efflux complex.	resistance-nodulation-cell division (RND) antibiotic efflux pump	diaminopyrimidine antibiotic;fluoroquinolone antibiotic;phenicol antibiotic	Multidrug	Antibiotic efflux	688	264	336	166	128	640	140	194	274	352	512	502	4196
ARO:3000806	MexG	MexG is a membrane protein required for MexGHI-OpmD efflux activity.	resistance-nodulation-cell division (RND) antibiotic efflux pump	acridine dye;fluoroquinolone antibiotic;tetracycline antibiotic	Multidrug	Antibiotic efflux	0	20	0	4	2	2	0	2	0	0	0	2	32
ARO:3000807	MexH	MexH is the membrane fusion protein of the efflux complex MexGHI-OpmD.	resistance-nodulation-cell division (RND) antibiotic efflux pump	acridine dye;fluoroquinolone antibiotic;tetracycline antibiotic	Multidrug	Antibiotic efflux	674	146	680	616	4520	380	804	1248	3864	4312	4056	5826	27126
ARO:3000808	MexI	MexI is the inner membrane transporter of the efflux complex MexGHI-OpmD.	resistance-nodulation-cell division (RND) antibiotic efflux pump	acridine dye;fluoroquinolone antibiotic;tetracycline antibiotic	Multidrug	Antibiotic efflux	360	292	466	228	1694	322	320	254	1694	1352	1392	1842	10216
ARO:3000809	OpmD	OpmD is the outer membrane channel protein of the efflux complex MexGHI-OpmD.	resistance-nodulation-cell division (RND) antibiotic efflux pump	acridine dye;fluoroquinolone antibiotic;tetracycline antibiotic	Multidrug	Antibiotic efflux	0	6	76	62	12	8	224	90	12	82	138	112	822
ARO:3000810	mtrC	MtrC is the membrane fusion protein of the MtrCDE multidrug efflux complex.	resistance-nodulation-cell division (RND) antibiotic efflux pump	macrolide antibiotic;penam	Multidrug	Antibiotic efflux	140	142	322	132	38	198	250	170	26	258	296	140	2112
ARO:3000811	mtrD	MtrD is the inner membrane multidrug transporter of the MtrCDE efflux complex.	resistance-nodulation-cell division (RND)	macrolide antibiotic;penam	Multidrug	Antibiotic efflux	38	40	314	172	304	82	338	276	208	2654	2088	3668	10182

ARO:3000812	mtrE	MtrE is an outer membrane exporter protein that is part of the MtrCDE multidrug efflux complex.	antibiotic efflux pump resistance-nodulation-cell division (RND) antibiotic efflux pump	macrolide antibiotic;penam	Multidrug	Antibiotic efflux	44	74	84	58	44	54	46	84	40	732	542	962	2764
ARO:3000815	mgrA	MgrA, also known as NorR, is a regulator for norA, norB, and tet38. It is a positive regulator for norA expression, but is a direct repressor for norB and an indirect repressor of tet38.	ATP-binding cassette (ABC) antibiotic efflux pump;major facilitator superfamily (MFS) antibiotic efflux pump	acridine dye;cephalosporin;fluoroquinolone antibiotic;penam;peptide antibiotic;tetracycline antibiotic	Multidrug	Antibiotic efflux	110	60	58	6	22	84	54	18	22	76	186	80	776
ARO:3000816	mtrA	MtrA is a transcriptional activator of the MtrCDE multidrug efflux pump of Neisseria gonorrhoeae.	resistance-nodulation-cell division (RND) antibiotic efflux pump	macrolide antibiotic;penam	Multidrug	Antibiotic efflux	5490	5614	5570	4928	6486	6142	5674	5814	6836	10122	11564	13126	87366
ARO:3000822	pmrA	PmrA is a MFS-type efflux pump expressed in Streptococcus pneumoniae that confers low-level resistance to norfloxacin, ciprofloxacin, and acriflavine.	major facilitator superfamily (MFS) antibiotic efflux pump	fluoroquinolone antibiotic	Fluoroquinolone	Antibiotic efflux	56	28	330	2	184	22	6	14	122	366	586	496	2212
ARO:3000823	ramA	RamA (resistance antibiotic multiple) is a positive regulator of AcrAB-TolC and leads to high level multidrug resistance in Klebsiella pneumoniae, Salmonella enterica, and Enterobacter aerugenes, increasing the expression of both the mar operon as well as AcrAB. RamA also decreases OmpF expression.	General Bacterial Porin with reduced permeability to beta-lactams;resistance-nodulation-cell division (RND) antibiotic efflux pump	carbapenem;cephalosporin;cephamycin;fluoroquinolone antibiotic;glycylcycline;monobactam;penam;penem;phenicol antibiotic;rifamycin antibiotic;tetracycline antibiotic;triclosan	Multidrug	Antibiotic efflux;reduced permeability to antibiotic	1174	662	534	1056	608	600	1546	1282	658	378	320	234	9052
ARO:3000826	sdiA	SdiA is a cell division regulator that is also a positive regulator	resistance-nodulation-	cephalosporin;fluoroquinolone	Multidrug	Antibiotic efflux	400	134	94	506	258	136	742	790	232	108	84	54	3538

		of AcrAB only when it's expressed from a plasmid. When the sdiA gene is on the chromosome, it has no effect on expression of acrAB	cell division (RND) antibiotic efflux pump	antibiotic;glycylcycline;penam;phenicol antibiotic;rifamycin antibiotic;tetracycline antibiotic;triclosan																
ARO:3000828	baeR	BaeR is a response regulator that promotes the expression of MdtABC and AcrD efflux complexes.	resistance-nodulation-cell division (RND) antibiotic efflux pump	aminocoumarin antibiotic;aminoglycoside antibiotic	Multidrug	Antibiotic efflux	2426	2602	2278	3054	2614	1710	4296	3324	2284	3282	3330	3754	34954
ARO:3000829	baeS	BaeS is a sensor kinase in the BaeSR regulatory system. While it phosphorylates BaeR to increase its activity, BaeS is not necessary for overexpressed BaeR to confer resistance.	resistance-nodulation-cell division (RND) antibiotic efflux pump	aminocoumarin antibiotic;aminoglycoside antibiotic	Multidrug	Antibiotic efflux	6794	7178	6340	5124	6582	7430	5628	5966	6886	12956	12494	16012	99390
ARO:3000830	cpxA	CpxA is a membrane-localized sensor kinase that is activated by envelope stress. It starts a kinase cascade that activates CpxR, which promotes efflux complex expression.	resistance-nodulation-cell division (RND) antibiotic efflux pump	aminocoumarin antibiotic;aminoglycoside antibiotic	Multidrug	Antibiotic efflux	4246	3838	4278	7252	16344	3250	10160	9982	14652	16662	16590	22046	129300
ARO:3000832	evgA	EvgA, when phosphorylated, is a positive regulator for efflux protein complexes emrKY and mdtEF. While usually phosphorylated in a EvgS dependent manner, it can be phosphorylated in the absence of EvgS when overexpressed.	major facilitator superfamily (MFS) antibiotic efflux pump;resistance-nodulation-cell division (RND) antibiotic efflux pump	fluoroquinolone antibiotic;macrolide antibiotic;penam;tetracycline antibiotic	Multidrug	Antibiotic efflux	4104	2418	2522	3268	2694	2608	4726	4018	2842	2170	2298	2456	36124
ARO:3000833	evgS	EvgS is a sensor protein that phosphorylates the regulatory protein EvgA. evgS corresponds to 1 locus in Pseudomonas aeruginosa PAO1 and 1 locus in Pseudomonas aeruginosa LESB58.	major facilitator superfamily (MFS) antibiotic efflux pump;resista	fluoroquinolone antibiotic;macrolide antibiotic;penam;tetracycline antibiotic	Multidrug	Antibiotic efflux	19064	15552	14500	15514	28772	14200	20340	21002	29780	25110	22942	27384	254160

			nce-nodulation-cell division (RND) antibiotic efflux pump																
ARO:3000838	arlR	ArlR is a response regulator that binds to the norA promoter to activate expression. ArlR must first be phosphorylated by ArlS.	major facilitator superfamily (MFS) antibiotic efflux pump	acridine dye;fluoroquinolone antibiotic	Multidrug	Antibiotic efflux	8972	7126	7420	6498	13220	8122	7494	8856	13628	14376	13932	18122	127766
ARO:3000839	arlS	ArlS is a protein histidine kinase that phosphorylates ArlR, a promoter for norA expression.	major facilitator superfamily (MFS) antibiotic efflux pump	acridine dye;fluoroquinolone antibiotic	Multidrug	Antibiotic efflux	8890	7120	5446	3246	2612	9792	3184	3912	3550	5760	4950	5500	63962
ARO:3000842	EBR-1 beta-lactamase	EBR-1 is an Ambler class B beta-lactamase found in Empedobacter brevis and is known to mediate the hydrolysis of penicillins, cephalosporins, and carbapenems efficiently but not aztreonam.	EBR beta-lactamase	carbapenem;cephalosporin;penam	Beta-lactam	Antibiotic inactivation	0	0	8	0	0	0	0	0	0	0	0	0	8
ARO:3000843	MUS-1	MUS-1 is a chromosome-encoded beta-lactamase from Myroides odoratus and Myroides odoratimimus.	MUS beta-lactamase	carbapenem	Beta-lactam	Antibiotic inactivation	0	2	14	0	20	0	2	0	0	100	114	104	356
ARO:3000847	KHM-1	KHM-1 is a plasmid-mediated metallo-beta-lactamase found in Citrobacter freundii that confers resistance to all broad-spectrum beta-lactams, execpt for monobactams.	KHM beta-latamase	carbapenem;cephalosporin;cephamycin;penam;penem	Beta-lactam	Antibiotic inactivation	0	0	140	0	2	0	0	0	2	0	0	0	144
ARO:3000850	GOB-1	GOB-1 confers resistance to cephalosporins in Elizabethkingia (Chryseobacterium) meningoseptica.	GOB beta-lactamase	carbapenem;cephalosporin;penam	Beta-lactam	Antibiotic inactivation	2	12	14	4	8	0	16	4	4	0	0	0	64
ARO:3000851	THIN-B	THIN-B, isolated from Janthinobacterium lividum, hydrolyzes a broad spectrum of beta-lactams including penicillins, cephalosporins, and carbapenems.	THIN-B beta-lactamase	carbapenem;cephalosporin;penam	Beta-lactam	Antibiotic inactivation	0	2	2	0	0	2	32	8	0	0	0	0	46

ARO:3000853	AIM-1	AIM-1 is an Ambler class B beta-lactamase that hydrolyzes most beta-lactams except aztreonam and ceftazidime. It was isolated from Pseudomonas aeruginosa and was the first subclass B3 mobile-elements encoded beta-lactamase discovered.	AIM beta-lactamase	cephalosporin;cephamycin;penam	Beta-lactam	Antibiotic inactivation	4	4	2	8	12	8	28	22	14	52	40	10	204
ARO:3000854	SMB-1	SMB-1 can hydrolyze a variety of beta-lactams, including penicillins, cephalosporins, and carbapenems. It was identified in Serratia marcescens.	SMB beta-lactamase	carbapenem;cephalosporin;penam	Beta-lactam	Antibiotic inactivation	2	20	10	12	18	4	24	26	14	0	0	0	130
ARO:3000855	CAU-1	CAU-1 is a B3 metallo-beta-lactamase that is encoded by the Caulobacter crescentus chromosome	CAU beta-lactamase	carbapenem;penam	Beta-lactam	Antibiotic inactivation	0	0	12	0	0	0	0	0	2	0	0	0	14
ARO:3000856	BJP-1	BJP-1 is a subclass B3 ortholog found in Bradyrhizobium japonicum that hydrolyzes most beta-lactams except aztreonam, ticarcillin, and temocillin.	BJP beta-lactamase	carbapenem	Beta-lactam	Antibiotic inactivation	22	14	74	54	20	26	46	66	16	36	14	8	396
ARO:3000865	oleD	OleD is a glycotransferase found in Streptomyces antibioticus, a natural producer of oleandomycin. OleD can glycosylate a wide range of macrolides. Unlike oleI, oleD is not found in the oleandomycin biosynthetic cluster.	ole glycosyltransferase	macrolide antibiotic	MLS	Antibiotic inactivation	4	24	16	0	36	14	6	12	48	16	0	4	180
ARO:3000866	oleI	OleI is a glycosyltransferase found in Streptomyces antibioticus, specifically the oleandomycin biosynthetic cluster. OleI glycosylates oleandomycin to confer self-resistance.	ole glycosyltransferase	macrolide antibiotic	MLS	Antibiotic inactivation	8	68	46	100	66	38	62	110	58	26	22	6	610
ARO:3000873	TEM-1	TEM-1 is a broad-spectrum beta-lactamase found in many Gram-negative bacteria. Confers resistance to penicillins and first generation cephalosphorins.	TEM beta-lactamase	cephalosporin;monobactam;penam;penem	Beta-lactam	Antibiotic inactivation	670	230	198	1452	844	56	1932	1596	642	244	286	110	8260
ARO:3000968	TEM-105	TEM-105 is a beta-lactamase found in E. coli.	TEM beta-lactamase	cephalosporin;monobactam;penam;penem	Beta-lactam	Antibiotic inactivation	0	0	0	0	0	0	0	0	0	2	8	0	10

ARO:3001013	TEM-146	TEM-146 is a beta-lactamase found in E. coli.	TEM beta-lactamase	cephalosporin;monobactam;penam;penem	Beta-lactam	Antibiotic inactivation	0	0	6	0	0	0	0	0	0	72	70	122	270
ARO:3001068	SHV-9	SHV-9 is an extended-spectrum beta-lactamase found in Klebsiella pneumoniae.	SHV beta-lactamase	carbapenem;cephalosporin;penam	Beta-lactam	Antibiotic inactivation	0	0	18	8	0	0	22	24	2	0	2	0	76
ARO:3001205	BRP(MBL)	A novel bleomycin resistance protein encoded by a metallo-beta-lactamase-associated ble gene. Expression of BRP(MBL) confers resistance to bleomycin and bleomycin-like antibiotics in Enterobacteriaceae and Acinetobacter, where it is co-expressed with an MBL and controlled by the same promoter region.	Bleomycin resistant protein	glycopeptide antibiotic	Glycopeptide	Antibiotic inactivation	2	4	12	6	102	0	14	6	96	26	26	28	322
ARO:3001209	mecC	A foreign PBP2a acquired by lateral gene transfer that able to perform peptidoglycan synthesis in the presence of beta-lactams.	methicillin resistant PBP2	carbapenem;cephalosporin;cephamycin;monobactam;penam	Beta-lactam	Antibiotic target replacement	1026	850	1000	526	332	1076	488	574	300	1566	1434	1754	10926
ARO:3001214	mdtM	Multidrug resistance protein MdtM	major facilitator superfamily (MFS) antibiotic efflux pump	acridine dye;fluoroquinolone antibiotic;lincosamide antibiotic;nucleoside antibiotic;phenicol antibiotic	Multidrug	Antibiotic efflux	610	320	206	1260	728	84	1906	1448	590	328	350	226	8056
ARO:3001216	mdtH	Multidrug resistance protein MdtH	major facilitator superfamily (MFS) antibiotic efflux pump	fluoroquinolone antibiotic	Multidrug	Antibiotic efflux	394	186	150	930	404	76	1462	1040	372	158	178	72	5422
ARO:3001265	Erm(30)	Erm(30) confers a MLSb resistant phenotype. Along with erm(31), these genes are responsible for self-resistance in the pikromycin/narbomycin/methymycin/neomethymycin	Erm 23S ribosomal RNA methyltransferase	lincosamide antibiotic;macrolide antibiotic;streptogramin antibiotic	MLS	Antibiotic target alteration	954	152	240	196	840	1114	332	278	750	460	602	516	6434

		producer, Streptomyces venezuelae.																	
ARO:3001299	tlrB conferring tylosin resistance	TlrB is a methyltransferase found in Streptomyces fradiae and confers resistance to mycinamicin, tylosin and lincosamides. Specifically, this enzyme adds a methyl group to guanosine 748 (E. coli numbering). TlrB is found in the tylosin biosynthetic cluster and is one mechanism by which S. fradiae protects itself from self-destruction when producing this macrolide.	non-erm 23S ribosomal RNA methyltransferase (G748)	lincosamide antibiotic;macrolide antibiotic	MLS	Antibiotic target alteration	14	34	212	2	124	26	10	4	110	752	786	1056	3130
ARO:3001300	myrA	myrA is a methyltransferase found in Micromonospora griseorubida and confers resistance to mycinamicin, tylosin and lincosamides. Specifically, this enzyme adds a methyl group to guanosine 748 (E. coli numbering) of 23S ribosomal RNA. MyrA is found in the mycinamicin biosynthetic cluster and is one mechanism by which M. griseorubida protects itself from self-destruction when producing this macrolide.	non-erm 23S ribosomal RNA methyltransferase (G748)	lincosamide antibiotic;macrolide antibiotic	MLS	Antibiotic target alteration	42	64	88	186	206	60	210	164	220	854	1760	1338	5192
ARO:3001301	RlmA(II)	RlmA(II) is a methyltransferase found in Streptococcus pneumoniae and confers resistance to mycinamicin, tylosin and lincosamides. Specifically, this enzyme adds a methyl group to guanosine 748 (E. coli numbering) of 23S ribosomal RNA.	non-erm 23S ribosomal RNA methyltransferase (G748)	lincosamide antibiotic;macrolide antibiotic	MLS	Antibiotic target alteration	2402	4038	3874	2156	1614	3788	1862	2724	2086	2306	1790	1822	30462
ARO:3001302	chrB	ChrB is a methyltransferase found in Streptomyces bikiniensis and confers resistance to chalcomycin, mycinamicin, tylosin and lincosamides. Specifically, this enzyme adds a methyl group to guanosine 748 (E. coli numbering). chrB is found in the	non-erm 23S ribosomal RNA methyltransferase (G748)	lincosamide antibiotic;macrolide antibiotic	MLS	Antibiotic target alteration	766	1740	538	610	2602	1016	610	962	2510	1158	890	1262	14664

		chalcomycin biosynthetic cluster and is responsible for self-resistance in S. bikiniensis.																
ARO:3001303	ErmO-srmA	ErmO (gene srmA) is a methyltransferase found in the spiramycin producer Streptomyces ambofaciens. Like other Erm enzymes, it catalyzes the methylation of A2058 of the 23S ribosomal RNA. Specifically, this enzyme transfers only one methyl group. The gene is responsible for self-resistance to spiramycin.	Erm 23S ribosomal RNA methyltransferase	lincosamide antibiotic;macrolide antibiotic;streptogramin antibiotic	MLS	Antibiotic target alteration	44	16	8	2	2	28	4	0	2	2	6	0	114
ARO:3001304	ErmS	ErmS is a methyltransferase found in the tylosin producer Streptomyces fradiae. Like other Erm enzymes, it catalyzes the methylation of A2058 of the 23S ribosomal RNA. Specifically, this enzyme transfers two methyl groups. The gene is found within the tylosin biosynthetic cluster and is responsible for self-resistance	Erm 23S ribosomal RNA methyltransferase	lincosamide antibiotic;macrolide antibiotic;streptogramin antibiotic	MLS	Antibiotic target alteration	0	0	44	0	36	0	6	0	32	60	86	148	412
ARO:3001306	ErmW	ErmW is a methyltransferase found in the mycinamicin producer Micromonospora griseorubida. Like other Erm enzymes, it catalyzes the methylation of A2058 of the 23S ribosomal RNA. The gene is found within the mycinamicin biosynthetic cluster and is responsible for self-resistance.	Erm 23S ribosomal RNA methyltransferase	lincosamide antibiotic;macrolide antibiotic;streptogramin antibiotic	MLS	Antibiotic target alteration	2	22	168	30	172	10	8	40	226	338	292	398	1706
ARO:3001313	facT	Efflux protein facT confers resistance to factumycin. The gene has been heterologously expressed in S. coelicolor and its function was confirmed (Thaker et al. Med. Chem. Commun. 2012, 3: 1020). It is possible that this gene can efflux other kirromycin-like elfamycins, but this has not been tested.	major facilitator superfamily (MFS) antibiotic efflux pump	elfamycin antibiotic	Elfamycin	Antibiotic efflux	190	40	170	78	206	236	150	134	260	398	592	608	3062
ARO:3001327	MdtK	A multidrug and toxic compound extrusions (MATE)	multidrug and toxic	fluoroquinolone antibiotic	Multidrug	Antibiotic efflux	984	786	642	932	744	528	1482	1134	780	208	202	98	8520

		transporter conferring resistance to norfloxacin, doxorubicin and acriflavine.	compound extrusion (MATE) transporter																
ARO:3001328	Escherichia coli mdfA	Multidrug efflux pump in E. coli. This multidrug efflux system was originally identified as the Cmr/CmlA chloramphenicol exporter.	major facilitator superfamily (MFS) antibiotic efflux pump	benzalkonium chloride;rhodamine;tetracycline antibiotic	Multidrug	Antibiotic efflux	928	1158	234	1076	586	576	1402	1088	450	326	312	320	8456
ARO:3001329	mdtG	The MdtG protein, also named YceE, appears to be a member of the major facilitator superfamily of transporters, and it has been reported, when overexpressed, to increase fosfomycin and deoxycholate resistances. mdtG is a member of the marA-soxS-rob regulon.	major facilitator superfamily (MFS) antibiotic efflux pump	fosfomycin	Fosfomycin	Antibiotic efflux	1378	722	202	928	426	1420	1338	1028	388	126	122	30	8108
ARO:3001392	TEM-215	From the Lahey list of beta-lactamases. Not yet released.	TEM beta-lactamase	cephalosporin;monobactam;penam;penem	Beta-lactam	Antibiotic inactivation	24	132	0	28	8	86	10	14	14	46	22	46	430
ARO:3001396	OXA-1	OXA-1 is a beta-lactamase found in E. coli	OXA beta-lactamase	cephalosporin;penam	Beta-lactam	Antibiotic inactivation	0	0	32	46	104	0	78	90	142	512	440	582	2026
ARO:3001398	OXA-3	OXA-3 is a beta-lactamase found in P. aeruginosa	OXA beta-lactamase	cephalosporin;penam	Beta-lactam	Antibiotic inactivation	0	0	14	0	236	0	8	84	220	104	68	128	862
ARO:3001405	OXA-10	OXA-10 is a beta-lactamase found in Acinetobacter baumannii and P. aeruginosa.	OXA beta-lactamase	cephalosporin;penam	Beta-lactam	Antibiotic inactivation	6	6	24	56	12	0	42	132	4	124	120	238	764
ARO:3001408	OXA-13	OXA-13 is a beta-lactamase found in P. aeruginosa.	OXA beta-lactamase	cephalosporin;penam	Beta-lactam	Antibiotic inactivation	0	12	6	0	0	0	12	6	2	0	2	2	42
ARO:3001413	OXA-18	OXA-18 is a beta-lactamase found in P. aeruginosa and Rickettsia.	OXA beta-lactamase	cephalosporin;penam	Beta-lactam	Antibiotic inactivation	0	0	0	0	0	0	0	0	4	0	0	0	4
ARO:3001416	OXA-21	OXA-21 is a beta-lactamase found in A. baumannii	OXA beta-lactamase	cephalosporin;penam	Beta-lactam	Antibiotic inactivation	0	4	186	30	162	12	66	26	276	402	360	526	2050

ARO:3001424	OXA-29	OXA-29 is a beta-lactamase found in Legionella gormanii	OXA beta-lactamase	cephalosporin;penam	Beta-lactam	Antibiotic inactivation	0	0	2	0	14	0	0	0	0	10	14	16	56
ARO:3001483	OXA-205	OXA-205 is a beta-lactamase. From the Lahey list of OXA beta-lactamases.	OXA beta-lactamase	cephalosporin;penam	Beta-lactam	Antibiotic inactivation	0	0	116	70	1086	2	58	444	986	274	234	288	3558
ARO:3001549	OXA-362	OXA-362 is a beta-lactamase. From the Lahey list of OXA beta-lactamases.	OXA beta-lactamase	cephalosporin;penam	Beta-lactam	Antibiotic inactivation	298	120	2	4	4	18	0	0	6	10	10	10	482
ARO:3001558	OXA-372	OXA-372 is a beta-lactamase. From the Lahey list of OXA beta-lactamases.	OXA beta-lactamase	cephalosporin;penam	Beta-lactam	Antibiotic inactivation	0	0	0	0	0	0	4	4	0	40	24	54	126
ARO:3001611	OXA-58	OXA-58 is a beta-lactamase found in A. baumannii	OXA beta-lactamase	cephalosporin;penam	Beta-lactam	Antibiotic inactivation	48	140	6	122	18	94	126	154	14	36	20	30	808
ARO:3001676	OXA-235	OXA-235 is a beta-lactamase found in A. baumannii	OXA beta-lactamase	cephalosporin;penam	Beta-lactam	Antibiotic inactivation	0	0	2	4	0	0	0	0	0	0	2	4	12
ARO:3001730	OXA-274	OXA-274 is a beta-lactamase found in Acinetobacter spp.	OXA beta-lactamase	cephalosporin;penam	Beta-lactam	Antibiotic inactivation	0	0	2	0	14	0	0	0	24	0	0	0	40
ARO:3001731	OXA-275	OXA-275 is a beta-lactamase found in Acinetobacter spp.	OXA beta-lactamase	cephalosporin;penam	Beta-lactam	Antibiotic inactivation	0	0	0	0	12	0	0	0	6	0	0	0	18
ARO:3001733	OXA-277	OXA-277 is a beta-lactamase found in Acinetobacter spp.	OXA beta-lactamase	cephalosporin;penam	Beta-lactam	Antibiotic inactivation	26	176	2	12	24	114	10	10	24	26	4	34	462
ARO:3001773	OXA-61	OXA-61 is a beta-lactamase found in Campylobacter jejuni	OXA beta-lactamase	cephalosporin;penam	Beta-lactam	Antibiotic inactivation	26	2	0	2	0	38	120	26	0	4	4	4	226
ARO:3001775	OXA-119	OXA-119 is a beta-lactamase found in Enterobacteriaceae	OXA beta-lactamase	cephalosporin;penam	Beta-lactam	Antibiotic inactivation	0	0	2	8	4	0	78	36	0	96	60	126	410
ARO:3001777	OXA-347	OXA-347 is a beta-lactamase found in Enterobacteriaceae	OXA beta-lactamase	cephalosporin;penam	Beta-lactam	Antibiotic	6	40	52	12	4	50	48	16	22	706	414	582	1952

						inactivation													
ARO:3001793	OXA-320	OXA-320 is a beta-lactamase found in Proteus mirabilis	OXA beta-lactamase	cephalosporin;penam	Beta-lactam	Antibiotic inactivation	0	4	4	2	0	8	8	4	2	0	0	4	36
ARO:3001797	OXA-46	OXA-46 is a beta-lactamase found in Pseudomonas aeruginosa	OXA beta-lactamase	cephalosporin;penam	Beta-lactam	Antibiotic inactivation	0	0	0	0	0	0	0	0	0	24	100	58	182
ARO:3001808	OXA-60	OXA-60 is a beta-lactamase found in Ralstonia pickettii	OXA beta-lactamase	cephalosporin;penam	Beta-lactam	Antibiotic inactivation	0	0	0	2	0	0	2	6	2	10	10	2	34
ARO:3001809	OXA-209	OXA-209 is a beta-lactamase found in Riemerella anatipestifer	OXA beta-lactamase	cephalosporin;penam	Beta-lactam	Antibiotic inactivation	4	2	4	2	2	8	6	4	2	84	66	84	268
ARO:3001810	OXA-53	OXA-53 is a beta-lactamase found in Salmonella enterica	OXA beta-lactamase	cephalosporin;penam	Beta-lactam	Antibiotic inactivation	0	2	6	0	580	0	0	0	494	1268	740	1862	4952
ARO:3001811	OXA-129	OXA-129 is a beta-lactamase found in Salmonella enterica	OXA beta-lactamase	cephalosporin;penam	Beta-lactam	Antibiotic inactivation	0	0	0	0	0	0	0	0	0	0	4	0	4
ARO:3001815	ACC-1	ACC-1 is a beta-lactamase found in Klebsiella pneumoniae	ACC beta-lactamase	cephalosporin;monobactam;penam	Beta-lactam	Antibiotic inactivation	2	2	46	28	16	0	8	30	14	6	0	0	152
ARO:3001816	ACC-2	ACC-2 is a beta-lactamase found in Hafnia alvei	ACC beta-lactamase	cephalosporin;monobactam;penam	Beta-lactam	Antibiotic inactivation	2	6	2	30	16	2	0	26	12	0	0	0	96
ARO:3001817	ACC-3	ACC-3 is a beta-lactamase found in Hafnia alvei	ACC beta-lactamase	cephalosporin;monobactam;penam	Beta-lactam	Antibiotic inactivation	14	2	86	50	14	12	68	60	18	66	22	46	458
ARO:3001818	ACC-4	ACC-4 is a beta-lactamase found in Escherichia coli	ACC beta-lactamase	cephalosporin;monobactam;penam	Beta-lactam	Antibiotic inactivation	0	10	2	8	0	2	8	12	0	60	46	32	180
ARO:3001819	ACC-5	ACC-5 is a beta-lactamase. From the Lahey list of ACC beta-lactamases. It is detected among Gram-negative bacteria isolates (Gamma- and Alpha-	ACC beta-lactamase	cephalosporin;monobactam;penam	Beta-lactam	Antibiotic inactivation	0	0	0	0	0	0	0	0	0	0	0	12	12

		proteobacteria) from urban water sources.																	
ARO:3001825	ACT-6	ACT-6 is a beta-lactamase found in Klebsiella pneumoniae	ACT beta-lactamase	carbapenem;cephalosporin;cephamycin;penam	Beta-lactam	Antibiotic inactivation	0	0	2	0	0	0	0	0	0	126	162	138	428
ARO:3001826	ACT-9	ACT-9 is a beta-lactamase found in Pantoea agglomerans	ACT beta-lactamase	carbapenem;cephalosporin;cephamycin;penam	Beta-lactam	Antibiotic inactivation	0	8	2	58	4	0	24	68	2	0	0	0	166
ARO:3001834	ACT-12	ACT-12 is a beta-lactamase. From the Lahey list of ACT beta-lactamases.	ACT beta-lactamase	carbapenem;cephalosporin;cephamycin;penam	Beta-lactam	Antibiotic inactivation	0	0	0	24	0	8	4	4	2	2	2	0	46
ARO:3001840	ACT-19	ACT-19 is a beta-lactamase. From the Lahey list of ACT beta-lactamases.	ACT beta-lactamase	carbapenem;cephalosporin;cephamycin;penam	Beta-lactam	Antibiotic inactivation	2	14	28	4	4	2	6	18	2	2	0	0	82
ARO:3001843	ACT-22	ACT-22 is a beta-lactamase. From the Lahey list of ACT beta-lactamases.	ACT beta-lactamase	carbapenem;cephalosporin;cephamycin;penam	Beta-lactam	Antibiotic inactivation	14	2	0	4	0	8	12	10	2	0	0	2	54
ARO:3001847	ACT-27	ACT-27 is a beta-lactamase. From the Lahey list of ACT beta-lactamases.	ACT beta-lactamase	carbapenem;cephalosporin;cephamycin;penam	Beta-lactam	Antibiotic inactivation	156	8	0	0	0	118	0	0	8	0	0	0	290
ARO:3001848	ACT-28	ACT-28 is a beta-lactamase. From the Lahey list of ACT beta-lactamases.	ACT beta-lactamase	carbapenem;cephalosporin;cephamycin;penam	Beta-lactam	Antibiotic inactivation	8	2	0	0	0	18	0	0	0	8	4	22	62
ARO:3001886	CTX-M-24	CTX-M-24 is a beta-lactamase found in the Enterobacteriaceae family	CTX-M beta-lactamase	cephalosporin	Beta-lactam	Antibiotic inactivation	20	0	0	62	40	0	72	54	24	2	2	2	278
ARO:3001917	CTX-M-55	CTX-M-55 is a beta-lactamase found in the Enterobacteriaceae family	CTX-M beta-lactamase	cephalosporin	Beta-lactam	Antibiotic inactivation	0	0	4	52	14	0	38	18	2	10	0	0	138
ARO:3001965	CTX-M-105	CTX-M-105 is a beta-lactamase found in Escherichia coli	CTX-M beta-lactamase	cephalosporin	Beta-lactam	Antibiotic inactivation	0	0	0	0	0	0	0	0	0	2	16	4	22
ARO:3001980	CTX-M-121	CTX-M-121 is a beta-lactamase found in Escherichia coli	CTX-M beta-lactamase	cephalosporin	Beta-lactam	Antibiotic inactivation	0	0	6	2	0	4	10	4	0	0	0	0	26

ARO:3002012	CMY-1	CMY-1 is a beta-lactamase found in the Enterobacteriaceae family	CMY beta-lactamase	cephamycin	Beta-lactam	Antibiotic inactivation	0	0	12	14	18	0	26	12	6	82	84	60	314
ARO:3002037	CMY-26	CMY-26 is a beta-lactamase found in the Enterobacteriaceae family	CMY beta-lactamase	cephamycin	Beta-lactam	Antibiotic inactivation	0	0	44	0	88	0	0	0	166	70	26	56	450
ARO:3002062	CMY-51	CMY-51 is a beta-lactamase. From the Lahey list of CMY beta-lactamases.	CMY beta-lactamase	cephamycin	Beta-lactam	Antibiotic inactivation	2	16	282	292	1688	20	294	564	1560	572	588	730	6608
ARO:3002086	CMY-73	CMY-73 is a beta-lactamase. From the Lahey list of CMY beta-lactamases.	CMY beta-lactamase	cephamycin	Beta-lactam	Antibiotic inactivation	104	230	172	88	52	86	52	70	80	14	6	8	962
ARO:3002106	CMY-93	CMY-93 is a beta-lactamase. From the Lahey list of CMY beta-lactamases.	CMY beta-lactamase	cephamycin	Beta-lactam	Antibiotic inactivation	0	0	0	0	0	0	0	0	0	20	0	14	34
ARO:3002112	CMY-100	CMY-100 is a beta-lactamase. From the Lahey list of CMY beta-lactamases.	CMY beta-lactamase	cephamycin	Beta-lactam	Antibiotic inactivation	20	0	0	0	0	0	0	0	4	0	0	0	24
ARO:3002125	CMY-114	CMY-114 is a beta-lactamase. From the Lahey list of CMY beta-lactamases.	CMY beta-lactamase	cephamycin	Beta-lactam	Antibiotic inactivation	2	0	2	0	0	0	0	0	2	14	30	16	66
ARO:3002133	DHA-2	DHA-2 is a beta-lactamase found in Klebsiella pneumoniae	DHA beta-lactamase	cephalosporin;cephamycin	Beta-lactam	Antibiotic inactivation	0	2	6	0	0	0	0	0	0	114	172	210	504
ARO:3002138	DHA-5	DHA-5 is a beta-lactamase. From the Lahey list of DHA beta-lactamases.	DHA beta-lactamase	cephalosporin;cephamycin	Beta-lactam	Antibiotic inactivation	66	2	0	0	2	16	12	0	0	0	0	0	98
ARO:3002140	DHA-9	DHA-9 is a beta-lactamase. From the Lahey list of DHA beta-lactamases.	DHA beta-lactamase	cephalosporin;cephamycin	Beta-lactam	Antibiotic inactivation	30	0	0	0	0	22	0	0	0	0	0	0	52
ARO:3002143	DHA-12	DHA-12 is a beta-lactamase. From the Lahey list of DHA beta-lactamases.	DHA beta-lactamase	cephalosporin;cephamycin	Beta-lactam	Antibiotic inactivation	8	20	16	0	2	24	0	0	10	6	2	6	94
ARO:3002147	DHA-16	DHA-16 is a beta-lactamase. From the Lahey list of DHA beta-lactamases.	DHA beta-lactamase	cephalosporin;cephamycin	Beta-lactam	Antibiotic	0	10	0	0	0	0	8	0	0	0	0	2	20

						inactivation													
ARO:3002153	DHA-22	DHA-22 is a beta-lactamase. From the Lahey list of DHA beta-lactamases.	DHA beta-lactamase	cephalosporin;cephamycin	Beta-lactam	Antibiotic inactivation	0	0	0	0	0	0	0	0	0	0	0	8	8
ARO:3002155	FOX-1	FOX-1 is a beta-lactamase found in Klebsiella pneumoniae	FOX beta-lactamase	cephalosporin;cephamycin	Beta-lactam	Antibiotic inactivation	0	0	0	0	0	0	4	0	0	16	8	0	28
ARO:3002157	FOX-3	FOX-3 is a beta-lactamase found in Klebsiella oxytoca	FOX beta-lactamase	cephalosporin;cephamycin	Beta-lactam	Antibiotic inactivation	0	0	0	10	0	0	4	4	0	0	0	0	18
ARO:3002162	FOX-10	FOX-10 is a beta-lactamase. From the Lahey list of FOX beta-lactamases.	FOX beta-lactamase	cephalosporin;cephamycin	Beta-lactam	Antibiotic inactivation	0	0	0	4	0	0	0	4	0	0	0	0	8
ARO:3002163	FOX-9	FOX-9 is a beta-lactamase. From the Lahey list of FOX beta-lactamases.	FOX beta-lactamase	cephalosporin;cephamycin	Beta-lactam	Antibiotic inactivation	0	0	2	0	2	0	0	0	22	4	14	4	48
ARO:3002167	MIR-4	MIR-4 is a beta-lactamase found in Escherichia coli	MIR beta-lactamase	cephalosporin;monobactam	Beta-lactam	Antibiotic inactivation	0	0	0	20	0	0	112	14	0	0	10	0	156
ARO:3002169	MIR-3	MIR-3 is a beta-lactamase. From the Lahey list of MIR beta-lactamases.	MIR beta-lactamase	cephalosporin;monobactam	Beta-lactam	Antibiotic inactivation	0	0	8	6	10	0	16	22	8	96	82	56	304
ARO:3002177	MIR-12	MIR-12 is a beta-lactamase. From the Lahey list of MIR beta-lactamases.	MIR beta-lactamase	cephalosporin;monobactam	Beta-lactam	Antibiotic inactivation	0	0	20	0	0	0	0	0	0	96	122	100	338
ARO:3002182	MOX-1	MOX-1 is a beta-lactamase found in Klebsiella pneumoniae	MOX beta-lactamase	cephalosporin;cephamycin;penam	Beta-lactam	Antibiotic inactivation	6	0	4	8	4	30	4	8	4	2	0	6	76
ARO:3002183	MOX-2	MOX-2 is a beta-lactamase found in Klebsiella pneumoniae	MOX beta-lactamase	cephalosporin;cephamycin;penam	Beta-lactam	Antibiotic inactivation	164	148	84	32	12	252	38	46	14	8	0	4	802
ARO:3002184	MOX-4	MOX-4 is a beta-lactamase found in Aeromonas caviae	MOX beta-lactamase	cephalosporin;cephamycin;penam	Beta-lactam	Antibiotic inactivation	0	0	0	0	0	0	0	0	0	222	86	192	500

ARO:3002185	MOX-6	MOX-6 is a beta-lactamase found in Klebsiella pneumoniae	MOX beta-lactamase	cephalosporin;cephamycin;penam	Beta-lactam	Antibiotic inactivation	14	8	26	0	2	8	8	0	14	0	2	2	84
ARO:3002186	MOX-3	MOX-3 is a beta-lactamase. From the Lahey list of MOX beta-lactamases.	MOX beta-lactamase	cephalosporin;cephamycin;penam	Beta-lactam	Antibiotic inactivation	0	0	10	6	10	0	16	4	10	18	18	58	150
ARO:3002191	MOX-9	MOX-9 is a beta-lactamase found in Citrobacter freundii.	MOX beta-lactamase	cephalosporin;cephamycin;penam	Beta-lactam	Antibiotic inactivation	0	0	0	28	0	0	14	112	0	0	0	0	154
ARO:3002226	IMP-35	IMP-35 is a beta-lactamase found in Pseudomonas aeruginosa	IMP beta-lactamase	carbapenem;cephalosporin;cephamycin;penam;penem	Beta-lactam	Antibiotic inactivation	0	0	8	2	2	0	62	12	0	40	38	102	266
ARO:3002242	CARB-3	CARB-3 is a beta-lactamase found in Pseudomonas aeruginosa	CARB beta-lactamase	penam	Beta-lactam	Antibiotic inactivation	30	558	112	84	302	48	120	126	254	2254	1728	3376	8992
ARO:3002243	CARB-4	CARB-4 is a beta-lactamase found in Pseudomonas aeruginosa	CARB beta-lactamase	penam	Beta-lactam	Antibiotic inactivation	0	0	40	2	108	0	2	6	70	190	152	374	944
ARO:3002244	CARB-5	CARB-5 is a beta-lactamase found in Acinetobacter calcoaceticus	CARB beta-lactamase	penam	Beta-lactam	Antibiotic inactivation	0	0	0	2	0	0	8	2	0	22	28	28	90
ARO:3002248	CARB-9	CARB-9 is a beta-lactamase found in Vibrio cholerae	CARB beta-lactamase	penam	Beta-lactam	Antibiotic inactivation	0	0	94	54	756	0	40	434	860	268	168	212	2886
ARO:3002249	CARB-10	CARB-10 is a beta-lactamase found in Acinetobacter baumannii	CARB beta-lactamase	penam	Beta-lactam	Antibiotic inactivation	0	0	0	0	0	0	0	0	0	6	2	6	14
ARO:3002255	CARB-16	CARB-16 is a beta-lactamase. From the Lahey list of IMP beta-lactamases.	CARB beta-lactamase	penam	Beta-lactam	Antibiotic inactivation	8	2	12	24	44	2	14	42	170	66	60	46	490
ARO:3002327	KPC-17	KPC-17 is a beta-lactamase. From the Lahey list of KPC beta-lactamases.	KPC beta-lactamase	carbapenem;cephalosporin;monobactam;penam	Beta-lactam	Antibiotic inactivation	0	0	12	30	0	0	34	38	0	0	2	0	116
ARO:3002363	PER-1	PER-1 is a beta-lactamase found in Pseudomonas aeruginosa	PER beta-lactamase	carbapenem;cephalosporin;mono	Beta-lactam	Antibiotic	8	20	6	20	16	8	8	8	6	0	0	0	100

				bactam;penam;penem		inactivation												
ARO:3002364	PER-2	PER-2 is a beta-lactamase found in Salmonella typhimurium	PER beta-lactamase	carbapenem;cephalosporin;monobactam;penam;penem	Beta-lactam	Antibiotic inactivation	0	0	8	0	0	2	0	0	0	0	0	0	10
ARO:3002366	PER-4	PER-4 is a beta-lactamase. From the Lahey list of PER beta-lactamases.	PER beta-lactamase	carbapenem;cephalosporin;monobactam;penam;penem	Beta-lactam	Antibiotic inactivation	0	0	0	2	12	0	0	4	8	0	2	0	28
ARO:3002372	VEB-3	VEB-3 is a beta-lactamase found in Enterobacter cloacae	VEB beta-lactamase	cephalosporin;monobactam	Beta-lactam	Antibiotic inactivation	0	0	0	8	6	0	2	0	0	0	0	0	16
ARO:3002373	VEB-4	VEB-4 is a beta-lactamase found in Proteus mirabilis	VEB beta-lactamase	cephalosporin;monobactam	Beta-lactam	Antibiotic inactivation	0	0	0	0	8	0	2	0	6	0	0	0	16
ARO:3002376	VEB-7	VEB-7 is a beta-lactamase. From the Lahey list of VEB beta-lactamases.	VEB beta-lactamase	cephalosporin;monobactam	Beta-lactam	Antibiotic inactivation	0	0	0	2	0	0	2	4	0	166	114	190	478
ARO:3002387	BEL-3	BEL-3 is a beta-lactamase found in Pseudomonas aeruginosa	BEL beta-lactamase	cephalosporin;monobactam;penam	Beta-lactam	Antibiotic inactivation	0	0	0	0	0	0	6	0	0	0	0	0	6
ARO:3002481	AER-1	AER-1 is a beta-lactamase found in Aeromonas hydrophila	AER beta-lactamase	penam	Beta-lactam	Antibiotic inactivation	0	0	0	2	0	0	24	4	0	2	0	0	32
ARO:3002482	LRA-1	LRA-1 is a beta-lactamase isolated from soil samples in Alaska	class A LRA beta-lactamase	cephalosporin;penam	Beta-lactam	Antibiotic inactivation	0	0	0	10	0	0	52	10	4	50	48	40	214
ARO:3002483	LRA-5	LRA-5 is a beta-lactamase isolated from soil samples in Alaska	class A LRA beta-lactamase	cephalosporin;penam	Beta-lactam	Antibiotic inactivation	0	0	42	12	18	0	24	12	6	186	272	294	866
ARO:3002484	LRA-13	LRA-13 is a class D/class C fusion bifunctional beta-lactamase isolated from soil samples in Alaska.	class C LRA beta-lactamase;class D LRA beta-lactamase	cephalosporin;penam	Beta-lactam	Antibiotic inactivation	0	0	22	0	0	0	0	0	0	42	86	92	242
ARO:3002485	LRA-2	LRA-2 is a beta-lactamase isolated from soil samples in Alaska	subclass B3 LRA beta-lactamase	cephalosporin;penam	Beta-lactam	Antibiotic	110	36	42	66	8	38	36	40	8	46	74	158	662

						inactivation													
ARO:3002487	LRA-8	LRA-8 is a beta-lactamase isolated from soil samples in Alaska	subclass B3 LRA beta-lactamase	cephalosporin;penam	Beta-lactam	Antibiotic inactivation	110	90	72	148	34	98	92	170	58	100	62	44	1078
ARO:3002488	LRA-9	LRA-9 is a beta-lactamase isolated from soil samples in Alaska	subclass B3 LRA beta-lactamase	cephalosporin;penam	Beta-lactam	Antibiotic inactivation	184	18	0	2	0	146	0	4	2	0	0	0	356
ARO:3002489	LRA-10	LRA-10 is a beta-lactamase isolated from soil samples in Alaska	class C LRA beta-lactamase	cephalosporin;penam	Beta-lactam	Antibiotic inactivation	0	0	0	0	0	0	28	2	0	0	0	0	30
ARO:3002492	LRA-18	LRA-18 is a beta-lactamase isolated from soil samples in Alaska	class C LRA beta-lactamase	cephalosporin;penam	Beta-lactam	Antibiotic inactivation	0	0	20	6	24	0	18	2	10	160	224	170	634
ARO:3002493	SRT-1	SRT-1, isolated from Serratia marcescens, confers resistance to cephalosporins but not carbapenems, penems and monobactams.	SRT beta-lactamase	cephalosporin	Beta-lactam	Antibiotic inactivation	2	6	22	8	10	20	2	8	48	160	180	192	658
ARO:3002494	SRT-2	SRT-2, isolated from Serratia marcescens, is a chromosomal beta-lactamase that confers resistance to cefotaxime	SRT beta-lactamase	cephalosporin	Beta-lactam	Antibiotic inactivation	176	22	214	22	144	166	120	26	196	528	638	638	2890
ARO:3002498	PDC-2	PDC-2 is a extended-spectrum beta-lactamase found in Pseudomonas aeruginosa.	PDC beta-lactamase	carbapenem;cephalosporin;monobactam	Beta-lactam	Antibiotic inactivation	60	6	0	0	0	106	0	0	0	0	0	0	172
ARO:3002507	PDC-8	PDC-8 is a extended-spectrum beta-lactamase found in Pseudomonas aeruginosa.	PDC beta-lactamase	carbapenem;cephalosporin;monobactam	Beta-lactam	Antibiotic inactivation	0	38	6	0	0	10	0	2	0	4	0	0	60
ARO:3002509	PDC-10	PDC-10 is a extended-spectrum beta-lactamase found in Pseudomonas aeruginosa.	PDC beta-lactamase	carbapenem;cephalosporin;monobactam	Beta-lactam	Antibiotic inactivation	0	0	0	0	0	0	0	0	0	6	88	32	126
ARO:3002510	LRA-3	LRA-3 is a beta-lactamase isolated from soil samples in Alaska	subclass B3 LRA beta-lactamase	cephalosporin;penam	Beta-lactam	Antibiotic inactivation	0	0	0	2	0	6	2	0	0	0	0	0	10
ARO:3002511	LRA-12	LRA-12 is a beta-lactamase isolated from soil samples in Alaska	subclass B3 LRA beta-lactamase	cephalosporin;penam	Beta-lactam	Antibiotic inactivation	0	14	0	0	6	4	4	4	18	4	8	2	64

ARO:3002512	LRA-17	LRA-17 is a beta-lactamase isolated from soil samples in Alaska	subclass B3 LRA beta-lactamase	cephalosporin;penam	Beta-lactam	Antibiotic inactivation	42	28	24	2	2	18	8	6	4	6	6	2	148
ARO:3002513	LRA-19	LRA-19 is a beta-lactamase isolated from soil samples in Alaska	subclass B3 LRA beta-lactamase	cephalosporin;penam	Beta-lactam	Antibiotic inactivation	48	16	92	80	6	46	48	82	6	6	2	0	432
ARO:3002514	OCH-1	OCH-1 beta-lactamase is an Ambler class C chromosomal-encoded beta-lactamases in Ochrobactrum anthropi	OCH beta-lactamase	cephalosporin;cephamycin;monobactam;penam;penem	Beta-lactam	Antibiotic inactivation	0	0	0	0	0	0	0	0	2	4	2	4	12
ARO:3002516	OCH-3	OCH-3 beta-lactamase is an Ambler class C chromosomal-encoded beta-lactamases in Ochrobactrum anthropi	OCH beta-lactamase	cephalosporin;cephamycin;monobactam;penam;penem	Beta-lactam	Antibiotic inactivation	0	0	2	0	0	0	0	0	12	0	0	0	14
ARO:3002518	OCH-5	OCH-5 beta-lactamase is an Ambler class C chromosomal-encoded beta-lactamases in Ochrobactrum anthropi	OCH beta-lactamase	cephalosporin;cephamycin;monobactam;penam;penem	Beta-lactam	Antibiotic inactivation	0	0	10	0	0	0	0	0	6	0	0	0	16
ARO:3002522	novA	A type III ABC transporter, identified on the novobiocin biosynthetic gene cluster, involved in the transport and resistance of novobiocin.	ATP-binding cassette (ABC) antibiotic efflux pump	aminocoumarin antibiotic	Aminocoumarin	Antibiotic efflux	6542	6392	5888	3554	2742	7736	4392	3802	3776	8420	10378	10280	73902
ARO:3002528	AAC(3)-Ia	AAC(3)-Ia is an aminoglycoside acetyltransferase encoded by plasmids, transposons, integrons in S. marcescens, E. coli, Acinetobacter baumannii, Klebsiella pneumoniae, Klebsiella oxytoca, P. aeruginosa, Salmonella typhimurium and Proteus mirabilis	AAC(3)	aminoglycoside antibiotic	Aminoglycoside	Antibiotic inactivation	8	30	76	16	0	6	4	14	4	0	0	0	158
ARO:3002531	AAC(3)-Ic	AAC(3)-Ic is an integron-encoded aminoglycoside acetyltransferase in P. aeruginosa	AAC(3)	aminoglycoside antibiotic	Aminoglycoside	Antibiotic inactivation	4	0	0	2	0	0	4	4	4	0	2	0	20
ARO:3002533	AAC(3)-IIa	AAC(3)-IIa is a plasmid-encoded aminoglycoside acetyltransferase in K. pneumoniae, E. cloacae, Actinobacillus pleuropneumoniae, S. typhimurium, Citrobacter freundii, and P. aeruginosa.	AAC(3)	aminoglycoside antibiotic	Aminoglycoside	Antibiotic inactivation	10	0	0	22	0	2	20	52	0	0	0	0	106

ARO:3002 534	AAC(3)-IIb	AAC(3)-IIb is an aminoglycoside acetyltransferase in E. coli, A. faecalis and S. marcescens	AAC(3)	aminoglycoside antibiotic	Aminoglycoside	Antibiotic inactivation	0	0	0	0	0	0	0	0	2	0	16	0	18
ARO:3002 535	AAC(3)-IIc	AAC(3)-IIc is a plasmid-encoded aminoglycoside acetyltransferase in E. coli and P. aeruginosa	AAC(3)	aminoglycoside antibiotic	Aminoglycoside	Antibiotic inactivation	0	0	4	2	0	0	2	2	0	12	20	6	48
ARO:3002 536	AAC(3)-IIIa	AAC(3)-IIIa is a chromosomal-encoded aminoglycoside acetyltransferase in P. aeruginosa	AAC(3)	aminoglycoside antibiotic	Aminoglycoside	Antibiotic inactivation	708	10	2	0	0	926	0	0	36	0	0	4	1686
ARO:3002 539	AAC(3)-IV	AAC(3)-IV is a plasmid-encoded aminoglycoside acetyltransferase in E. coli, C. jejuni and P. stutzeri	AAC(3)	aminoglycoside antibiotic	Aminoglycoside	Antibiotic inactivation	96	218	40	134	42	186	58	154	94	50	140	102	1314
ARO:3002 541	AAC(3)-VIIa	AAC(3)-VIIa is a chromosomal-encoded aminoglycoside acetyltransferase in Streptomyces rimosus	AAC(3)	aminoglycoside antibiotic	Aminoglycoside	Antibiotic inactivation	84	46	0	2	30	58	0	0	6	0	0	0	226
ARO:3002 542	AAC(3)-VIIIa	AAC(3)-VIIIa is a chromosomal-encoded aminoglycoside acetyltransferase in Streptomyces fradiae	AAC(3)	aminoglycoside antibiotic	Aminoglycoside	Antibiotic inactivation	10	38	0	0	0	22	0	0	2	0	0	2	74
ARO:3002 543	AAC(3)-IXa	AAC(3)-IXa is a chromosomal-encoded aminoglycoside acetyltransferase in Micromonospora chalcea	AAC(3)	aminoglycoside antibiotic	Aminoglycoside	Antibiotic inactivation	8	2	8	4	0	2	2	0	0	0	0	0	26
ARO:3002 544	AAC(3)-Xa	AAC(3)-Xa is a chromosomal-encoded aminoglycoside acetyltransferase in Streptomyces griseus	AAC(3)	aminoglycoside antibiotic	Aminoglycoside	Antibiotic inactivation	4	34	18	8	420	30	4	16	382	0	0	0	916
ARO:3002 545	AAC(6')-Ia	AAC(6')-Ia is an aminoglycoside acetyltransferase encoded by plasmids, transposons, integrons in Citrobacter diversus, E. coli, K. pneumoniae, Shigella sonnei, and P. aeruginosa.	AAC(6')	aminoglycoside antibiotic	Aminoglycoside	Antibiotic inactivation	4	2	0	0	4	0	0	0	0	10	10	24	54
ARO:3002 560	AAC(6')-Iq	AAC(6')-Iq is a aminoglycoside acetyltransferase encoded by plasmids and integrons in K. pneumoniae	AAC(6')	aminoglycoside antibiotic	Aminoglycoside	Antibiotic inactivation	0	0	0	0	0	0	0	0	2	0	0	0	2
ARO:3002 561	AAC(6')-Ir	AAC(6')-Ir is a chromosomal-encoded aminoglycoside acetyltransferase in Acinetobacter colistiniresistens	AAC(6')	aminoglycoside antibiotic	Aminoglycoside	Antibiotic inactivation	0	0	14	0	0	4	0	0	0	4	0	0	22

ARO:3002562	AAC(6')-Is	AAC(6')-Is is a chromosomal-encoded aminoglycoside acetyltransferase in Acinetobacter variabilis	AAC(6')	aminoglycoside antibiotic	Aminoglycoside	Antibiotic inactivation	2	8	6	0	0	10	2	0	4	12	6	18	68
ARO:3002572	AAC(6')-Iad	AAC(6')-Iad is a plasmid-encoded aminoglycoside acetyltransferase in Acinetobacter genomosp. 3	AAC(6')	aminoglycoside antibiotic	Aminoglycoside	Antibiotic inactivation	276	166	274	138	90	268	134	196	92	160	80	62	1936
ARO:3002573	AAC(6')-Iae	AAC(6')-Iae is an integron-encoded aminoglycoside acetyltransferase in P. aeruginosa and S. enterica	AAC(6')	aminoglycoside antibiotic	Aminoglycoside	Antibiotic inactivation	12	0	0	0	0	0	0	0	0	0	0	0	12
ARO:3002579	AAC(6')-Ib8	AAC(6')-Ib8 is a plasmid-encoded aminoglycoside acetyltransferase in E. cloacae	AAC(6')	aminoglycoside antibiotic	Aminoglycoside	Antibiotic inactivation	0	2	10	0	0	0	0	0	0	2	6	6	26
ARO:3002584	AAC(6')-29b	AAC(6')-29b is an integron-encoded aminoglycoside acetyltransferase in P. aeruginosa	AAC(6')	aminoglycoside antibiotic	Aminoglycoside	Antibiotic inactivation	0	0	0	0	0	0	10	0	0	0	0	0	10
ARO:3002588	AAC(6')-I30	AAC(6')-I30 is an integron-encoded aminoglycoside acetyltransferase in S. enterica	AAC(6')	aminoglycoside antibiotic	Aminoglycoside	Antibiotic inactivation	16	58	108	46	20	40	38	34	42	14	10	8	434
ARO:3002589	AAC(6')-Iid	AAC(6')-Iid is a chromosomal-encoded aminoglycoside acetyltransferase in Enterococcus durans	AAC(6')	aminoglycoside antibiotic	Aminoglycoside	Antibiotic inactivation	0	0	0	0	0	0	0	0	0	12	20	2	34
ARO:3002594	AAC(6')-IIa	AAC(6')-IIa is an aminoglycoside acetyltransferase encoded by plasmids and integrons in P. aeruginosa and S. enterica	AAC(6')	aminoglycoside antibiotic	Aminoglycoside	Antibiotic inactivation	0	0	0	0	0	0	14	2	0	0	4	0	20
ARO:3002595	AAC(6')-IIb	AAC(6')-IIb is an integron-encoded aminoglycoside acetyltransferase in P. fluorescens	AAC(6')	aminoglycoside antibiotic	Aminoglycoside	Antibiotic inactivation	0	0	0	0	0	0	2	0	0	0	2	0	4
ARO:3002596	AAC(6')-IIc	AAC(6')-IIc is an aminoglycoside acetyltransferase encoded by plasmids and integrons in E. cloacae	AAC(6')	aminoglycoside antibiotic	Aminoglycoside	Antibiotic inactivation	0	0	0	0	2	0	2	12	4	68	58	78	224
ARO:3002597	AAC(6')-Ie-APH(2'')-Ia	AAC(6')-Ie-APH(2'')-Ia is an aminoglycoside acetyltransferase encoded by plasmids and transposons in S. aureus, E. faecalis, E. faecium and Staphylococcus warneri	AAC(6');APH(2'')	aminoglycoside antibiotic	Aminoglycoside	Antibiotic inactivation	110	56	202	36	36	52	66	72	34	214	280	268	1426

ARO:3002598	ANT(3'')-Ii-AAC(6')-IId fusion protein	ANT(3'')-Ii-AAC(6')-IId is an integron-encoded aminoglycoside acetyltransferase in S. marcescens.	AAC(6');ANT(3'')	aminoglycoside antibiotic	Aminoglycoside	Antibiotic inactivation	2	18	340	154	880	36	382	224	830	2390	2516	3938	11710
ARO:3002599	AAC(6')-30/AAC(6')-Ib' fusion protein	AAC(6')-30/AAC(6')-Ib' is an integron-encoded aminoglycoside acetyltransferase in P. aeruginosa	AAC(6')	aminoglycoside antibiotic	Aminoglycoside	Antibiotic inactivation	0	6	28	4	144	0	20	24	118	104	86	132	666
ARO:3002602	aadA2	aadA2 is an aminoglycoside nucleotidyltransferase gene encoded by plasmids and integrons in K. pneumoniae, Salmonella spp., Corynebacterium glutamicum, C. freundii and Aeromonas spp.	ANT(3'')	aminoglycoside antibiotic	Aminoglycoside	Antibiotic inactivation	400	798	312	1304	2484	160	2158	1432	2112	1288	1892	1702	16042
ARO:3002604	aadA4	aadA4 is an aminoglycoside nucleotidyltransferase gene encoded by plasmids and chromosomes in Bordetella parapertussis and E. coli	ANT(3'')	aminoglycoside antibiotic	Aminoglycoside	Antibiotic inactivation	0	0	0	0	0	0	0	0	0	0	6	6	12
ARO:3002605	aadA5	aadA5 is an aminoglycoside nucleotidyltransferase gene encoded by plasmids, transposons and integrons in E. coli, K. pneumoniae, Kluyvera georgiana, P. aeruginosa and E. cloacae	ANT(3'')	aminoglycoside antibiotic	Aminoglycoside	Antibiotic inactivation	4	4	76	72	300	8	142	80	274	344	442	568	2314
ARO:3002606	aadA6	aadA6 is an integron-encoded aminoglycoside nucleotidyltransferase gene in P. aeruginosa	ANT(3'')	aminoglycoside antibiotic	Aminoglycoside	Antibiotic inactivation	0	2	4	14	0	0	38	8	0	10	24	24	124
ARO:3002607	aadA7	aadA7 is an integron-encoded aminoglycoside nucleotidyltransferase gene in V. fluvialis, P. aeruginosa, E. coli, V. cholerae and S. enterica	ANT(3'')	aminoglycoside antibiotic	Aminoglycoside	Antibiotic inactivation	4436	5306	3116	2016	838	5290	1958	2516	1266	934	532	474	28682
ARO:3002609	aadA9	aadA9 is a plasmid-encoded aminoglycoside nucleotidyltransferase gene in C. glutamicum	ANT(3'')	aminoglycoside antibiotic	Aminoglycoside	Antibiotic inactivation	54	70	18	6	14	182	12	12	42	766	892	724	2792
ARO:3002611	aadA11	aadA11 is an integron-encoded aminoglycoside nucleotidyltransferase gene in E. coli and P. aeruginosa	ANT(3'')	aminoglycoside antibiotic	Aminoglycoside	Antibiotic inactivation	0	0	16	2	0	0	32	4	10	50	108	132	354

ARO:3002613	aadA13	aadA13 is an aminoglycoside nucleotidyltransferase gene encoded by plasmids and integrons in Pseudomonas rettgeri, P. aeruginosa, Y. enterocolitica and E. coli	ANT(3'')	aminoglycoside antibiotic	Aminoglycoside	Antibiotic inactivation	412	602	926	292	256	512	206	354	422	566	474	442	5464
ARO:3002614	aadA14	aadA14 is a plasmid-encoded aminoglycoside nucleotidyltransferase gene in Pasteurella multocida	ANT(3'')	aminoglycoside antibiotic	Aminoglycoside	Antibiotic inactivation	0	4	2	4	12	4	2	88	58	16	28	24	242
ARO:3002616	aadA16	aadA16 is an aminoglycoside nucleotidyltransferase gene encoded by plasmids and integrons in E. coli, V. cholerae and K. pneumoniae	ANT(3'')	aminoglycoside antibiotic	Aminoglycoside	Antibiotic inactivation	0	12	12	24	140	10	34	40	190	152	164	212	990
ARO:3002620	aadA23	aadA23 is an integron-encoded aminoglycoside nucleotidyltransferase gene in S. enterica	ANT(3'')	aminoglycoside antibiotic	Aminoglycoside	Antibiotic inactivation	0	0	0	0	0	0	0	0	0	0	0	6	6
ARO:3002626	ANT(6)-Ia	ANT(6)-Ia is an aminoglycoside nucleotidyltransferase gene encoded by plasmids and chromosomes in Staphylococcus epidermidis, E. faecium, Streptococcus suis, S. aureus, E. faecalis and Streptococcus mitis	ANT(6)	aminoglycoside antibiotic	Aminoglycoside	Antibiotic inactivation	242	264	364	106	172	270	138	212	230	416	436	494	3344
ARO:3002627	aadK	aadK is a chromosomal-encoded aminoglycoside nucleotidyltransferase gene in B. subtilis and Bacillus spp.	ANT(6)	aminoglycoside antibiotic	Aminoglycoside	Antibiotic inactivation	202	156	162	2	34	336	10	30	28	124	134	164	1382
ARO:3002628	aad(6)	aad(6) is a plasmid-encoded aminoglycoside nucleotidyltransferase gene in E. faecalis and Streptococcus oralis	ANT(6)	aminoglycoside antibiotic	Aminoglycoside	Antibiotic inactivation	264	274	492	268	188	254	280	300	318	188	158	120	3104
ARO:3002629	ANT(6)-Ib	ANT(6)-Ib is an aminoglycoside nucleotidyltransferase gene encoded by transferable pathogenicity islands in C. fetus subsp. fetus and B. subtilis	ANT(6)	aminoglycoside antibiotic	Aminoglycoside	Antibiotic inactivation	2534	3104	3046	1602	864	3136	1548	2100	1232	880	548	576	21170
ARO:3002630	ANT(9)-Ia	ANT(9)-Ia is an aminoglycoside nucleotidyltransferase encoded by plasmids and transposons in S. aureus, Enterococcus spp., Staphylococcus sciuri and E. faecalis	ANT(9)	aminoglycoside antibiotic	Aminoglycoside	Antibiotic inactivation	0	0	8	0	22	0	2	2	2	4	0	0	40
ARO:3002631	spd	spd is a plasmid-encoded aminoglycoside	ANT(9)	aminoglycoside antibiotic	Aminoglycoside	Antibiotic	4	0	4	6	60	22	0	2	146	54	88	84	470

		nucleotidyltransferase gene in S. aureus				inactivation													
ARO:3002634	APH(2'')-Ie	APH(2'')-Ie is a plasmid or transposon-encoded aminoglycoside phosphotransferase in E. faecium and E. casseliflavus	APH(2'')	aminoglycoside antibiotic	Aminoglycoside	Antibiotic inactivation	0	0	0	0	0	0	0	2	0	32	42	16	92
ARO:3002635	APH(2'')-IIa	APH(2'')-IIa is a chromosomal-encoded aminoglycoside phosphotransferase in E. faecium and E. coli	APH(2'')	aminoglycoside antibiotic	Aminoglycoside	Antibiotic inactivation	52	86	102	94	70	90	56	128	136	26	4	18	862
ARO:3002636	APH(2'')-IIIa	APH(2'')-IIIa is a plasmid-encoded aminoglycoside phosphotransferase in Enterococcus gallinarum	APH(2'')	aminoglycoside antibiotic	Aminoglycoside	Antibiotic inactivation	2	0	0	0	18	2	0	0	8	34	38	24	126
ARO:3002638	APH(3'')-Ia	APH(3'')-Ia is a chromosomal-encoded aminoglycoside phosphotransferase in S. griseus	APH(3'')	aminoglycoside antibiotic	Aminoglycoside	Antibiotic inactivation	0	0	0	2	2	0	14	0	0	16	44	6	84
ARO:3002639	APH(3'')-Ib	APH(3'')-Ib is an aminoglycoside phosphotransferase encoded by plasmids, transposons, integrative conjugative elements and chromosomes in Enterobacteriaceae and Pseudomonas spp.	APH(3'')	aminoglycoside antibiotic	Aminoglycoside	Antibiotic inactivation	498	606	968	1312	4000	614	1252	1928	3594	4530	4888	5838	30028
ARO:3002641	APH(3')-Ia	APH(3')-Ia is a transposon-encoded aminoglycoside phosphotransferase in E. coli and S. enterica. It is identical at the protein sequence to APH(3')-Ic, an aminoglycoside phosphotransferase encoded by plasmids, transposons and genomic islands in K. pneumoniae, A. baumannii, S. marcescens, Corynebacterium spp., Photobacterium spp. and Citrobacter spp.	APH(3')	aminoglycoside antibiotic	Aminoglycoside	Antibiotic inactivation	200	886	176	428	598	370	630	578	842	1026	1072	1502	8308
ARO:3002642	APH(3')-Ib	APH(3')-Ib is a plasmid-encoded aminoglycoside phosphotransferase in E. coli	APH(3')	aminoglycoside antibiotic	Aminoglycoside	Antibiotic inactivation	0	0	6	14	2	0	10	12	6	6	12	6	74
ARO:3002644	APH(3')-IIa	APH(3')-IIa is a transposon-encoded aminoglycoside phosphotransferase in E. coli	APH(3')	aminoglycoside antibiotic	Aminoglycoside	Antibiotic inactivation	0	6	0	24	10	6	24	14	4	64	54	68	274

ARO:3002647	APH(3')-IIIa	APH(3')-IIIa is a plasmid-encoded aminoglycoside phosphotransferase in S. aureus and Enterococcus spp.	APH(3')	aminoglycoside antibiotic	Aminoglycoside	Antibiotic inactivation	1764	1724	6924	3592	2142	1730	3064	4058	2778	1550	1044	1200	31570
ARO:3002648	APH(3')-IVa	APH(3')-IVa is a chromosomal-encoded aminoglycoside phosphotransferase in B. circulans	APH(3')	aminoglycoside antibiotic	Aminoglycoside	Antibiotic inactivation	92	86	18	6	16	100	66	16	14	62	54	46	576
ARO:3002649	APH(3')-Va	APH(3')-Va is a chromosomal-encoded aminoglycoside phosphotransferase in Streptomyces fradiae	APH(3')	aminoglycoside antibiotic	Aminoglycoside	Antibiotic inactivation	2	24	0	22	2	22	58	32	6	36	48	12	264
ARO:3002652	APH(3')-VIa	APH(3')-VIa is a plasmid-encoded aminoglycoside phosphotransferase in A. baumannii	APH(3')	aminoglycoside antibiotic	Aminoglycoside	Antibiotic inactivation	8	6	368	80	1778	0	142	470	1536	1162	1110	1534	8194
ARO:3002654	APH(3')-VIIa	APH(3')-VIIa is a plasmid-encoded aminoglycoside phosphotransferase in C. jejuni	APH(3')	aminoglycoside antibiotic	Aminoglycoside	Antibiotic inactivation	4	16	120	28	26	14	34	32	16	48	8	18	364
ARO:3002655	APH(4)-Ia	APH(4)-Ia is a plasmid-encoded aminoglycoside phosphotransferase in E. coli	APH(4)	aminoglycoside antibiotic	Aminoglycoside	Antibiotic inactivation	1470	844	1126	770	624	880	570	868	778	370	310	260	8870
ARO:3002657	APH(6)-Ia	APH(6)-Ia is a chromosomal-encoded aminoglycoside phosphotransferase in S. griseus	APH(6)	aminoglycoside antibiotic	Aminoglycoside	Antibiotic inactivation	0	0	0	0	0	0	20	10	0	8	10	6	54
ARO:3002659	APH(6)-Ic	APH(6)-Ic is a transposon-encoded aminoglycoside phosphotransferase in S. enterica, P. aeruginosa and E. coli	APH(6)	aminoglycoside antibiotic	Aminoglycoside	Antibiotic inactivation	0	8	2	22	0	6	8	10	6	56	44	56	218
ARO:3002660	APH(6)-Id	APH(6)-Id is an aminoglycoside phosphotransferase encoded by plasmids, integrative conjugative elements and chromosomal genomic islands in K. pneumoniae, Salmonella spp., E. coli, Shigella flexneri, Providencia alcalifaciens, Pseudomonas spp., V. cholerae, Edwardsiella tarda, Pasteurella multocida and Aeromonas bestiarum	APH(6)	aminoglycoside antibiotic	Aminoglycoside	Antibiotic inactivation	850	798	1640	1924	8368	800	1986	3638	7668	7774	7856	10288	53590
ARO:3002666	rmtF	RmtF is a 16S rRNA methyltransferase found in	16S rRNA methyltransf	aminoglycoside antibiotic	Aminoglycoside	Antibiotic target	10	0	14	6	10	24	16	8	8	6	4	0	106

		Pseudomonas aeruginosa which methylates G1405 of the 16S rRNA. It confers high level resistance to many aminoglycosides.	erase (G1405)			alteration												
ARO:3002667	rmtD	RmtD is a 16S rRNA methyltransferase found in Pseudomonas aeruginosa which methylates G1405 of the 16S rRNA. It confers high level resistance to many aminoglycosides.	16S rRNA methyltransferase (G1405)	aminoglycoside antibiotic	Aminoglycoside	Antibiotic target alteration	4	0	8	0	4	8	0	0	6	0	0	4	34
ARO:3002672	Bacillus pumilus cat86	cat86 is a chromosome-encoded variant of the cat gene found in Bacillus pumilus	chloramphenicol acetyltransferase (CAT)	phenicol antibiotic	Phenicol	Antibiotic inactivation	0	0	18	8	0	0	8	20	2	40	12	34	142
ARO:3002674	Clostridium butyricum catB	catB is a chromosome-encoded variant of the cat gene found in Clostridium butyricum	chloramphenicol acetyltransferase (CAT)	phenicol antibiotic	Phenicol	Antibiotic inactivation	8	6	8	24	44	0	20	38	70	16	6	12	252
ARO:3002675	catB2	catB2 is a plasmid-encoded variant of the cat gene found in Escherichia coli, Salmonella enteritidis and Pasteurella multocida	chloramphenicol acetyltransferase (CAT)	phenicol antibiotic	Phenicol	Antibiotic inactivation	12	118	90	12	184	12	38	42	184	62	36	76	866
ARO:3002676	catB3	catB3 is a plasmid or chromosome-encoded variant of the cat gene found in Salmonella typhimurium, Acinetobacter baumannii and Escherichia coli	chloramphenicol acetyltransferase (CAT)	phenicol antibiotic	Phenicol	Antibiotic inactivation	354	382	452	140	280	470	206	154	472	730	566	856	5062
ARO:3002678	Pseudomonas aeruginosa catB6	catB6 is a plasmid-encoded variant of the cat gene found in Pseudomonas aeruginosa	chloramphenicol acetyltransferase (CAT)	phenicol antibiotic	Phenicol	Antibiotic inactivation	16	68	10	4	4	28	10	8	18	10	8	2	186
ARO:3002679	Pseudomonas aeruginosa catB7	catB7 is a chromosome-encoded variant of the cat gene found in Pseudomonas aeruginosa	chloramphenicol acetyltransferase (CAT)	phenicol antibiotic	Phenicol	Antibiotic inactivation	4	14	0	6	2	0	16	4	52	0	0	0	98
ARO:3002680	catB8	catB8 is a plasmid or integron-encoded variant of the cat gene found in Klebsiella pneumoniae, Salmonella typhi and Pseudomonas aeruginosa	chloramphenicol acetyltransferase (CAT)	phenicol antibiotic	Phenicol	Antibiotic inactivation	246	176	200	66	294	366	126	88	314	1106	1138	1610	5730

ARO:3002681	catB9	catB9 is a chromosome-encoded variant of the cat gene found in Vibrio cholerae	chloramphenicol acetyltransferase (CAT)	phenicol antibiotic	Phenicol	Antibiotic inactivation	568	654	48	120	86	522	88	146	70	176	74	210	2762
ARO:3002683	catI	catI is a chromosome and transposon-encoded variant of the cat gene found in Escherichia coli and Acinetobacter baumannii	chloramphenicol acetyltransferase (CAT)	phenicol antibiotic	Phenicol	Antibiotic inactivation	126	108	80	12	94	94	34	16	100	8	2	0	674
ARO:3002684	catII	catII is a plasmid-encoded variant of the cat gene found in Haemophilus influenzae, Agrobacterium tumefaciens and Escherichia coli	chloramphenicol acetyltransferase (CAT)	phenicol antibiotic	Phenicol	Antibiotic inactivation	44	0	2	0	0	0	4	0	0	0	0	0	50
ARO:3002685	catIII	catIII is a plasmid-encoded variant of the cat gene found in Shigella flexneri	chloramphenicol acetyltransferase (CAT)	phenicol antibiotic	Phenicol	Antibiotic inactivation	2	6	8	44	6	2	14	60	12	20	16	24	214
ARO:3002686	catP	catP is a transposon and chromosome-encoded variant of the cat gene found in Clostridium perfringens and Neisseria meningitidis	chloramphenicol acetyltransferase (CAT)	phenicol antibiotic	Phenicol	Antibiotic inactivation	40	52	78	52	12	40	46	46	22	4	8	6	406
ARO:3002687	catQ	catQ is a chromosome-encoded variant of the cat gene found in Clostridium perfringens	chloramphenicol acetyltransferase (CAT)	phenicol antibiotic	Phenicol	Antibiotic inactivation	88	246	154	92	64	170	100	96	104	78	38	24	1254
ARO:3002690	Streptomyces lividans cmlR	cmlR is a plasmid or chromosome-encoded chloramphenicol resistance determinant (putative transmembrane protein) that is found in Escherichia coli and Streptomyces lividans	major facilitator superfamily (MFS) antibiotic efflux pump	phenicol antibiotic	Phenicol	Antibiotic efflux	22	8	76	48	0	10	94	48	10	1508	942	2460	5226
ARO:3002691	Salmonella enterica cmlA	cmlA is a plasmid-encoded chloramphenicol exporter that is found in Salmonella typhimurium	major facilitator superfamily (MFS) antibiotic efflux pump	phenicol antibiotic	Phenicol	Antibiotic efflux	0	0	0	0	0	0	0	0	0	4	2	0	6
ARO:3002693	cmlA1	cmlA1 is a plasmid or transposon-encoded chloramphenicol exporter that is found in Pseudomonas aeruginosa and Klebsiella pneumoniae	major facilitator superfamily (MFS) antibiotic efflux pump	phenicol antibiotic	Phenicol	Antibiotic efflux	110	354	50	438	108	32	516	472	100	116	92	136	2524

ARO:3002694	cmlA4	cmlA4 is a plasmid-encoded chloramphenicol exporter that is found in Klebsiella pneumoniae	major facilitator superfamily (MFS) antibiotic efflux pump	phenicol antibiotic	Phenicol	Antibiotic efflux	0	0	0	2	0	0	6	0	0	8	0	0	16
ARO:3002695	cmlA5	cmlA5 is a plasmid or transposon-encoded chloramphenicol exporter that is found in Escherichia coli	major facilitator superfamily (MFS) antibiotic efflux pump	phenicol antibiotic	Phenicol	Antibiotic efflux	104	388	24	564	132	28	672	540	110	102	104	120	2888
ARO:3002696	cmlA6	cmlA6 is a plasmid-encoded chloramphenicol exporter that is found in Pseudomonas aeruginosa	major facilitator superfamily (MFS) antibiotic efflux pump	phenicol antibiotic	Phenicol	Antibiotic efflux	52	154	6	224	52	18	214	208	30	1846	1336	2784	6924
ARO:3002698	cmlB	cmlB is a plasmid-encoded chloramphenicol exporter that is found in Klebsiella aerogenes	major facilitator superfamily (MFS) antibiotic efflux pump	phenicol antibiotic	Phenicol	Antibiotic efflux	704	218	162	754	372	368	1140	922	336	170	156	116	5418
ARO:3002699	cmlB1	cmlB1 is a plasmid-encoded chloramphenicol exporter that is found in Bordetella bronchiseptica	major facilitator superfamily (MFS) antibiotic efflux pump	phenicol antibiotic	Phenicol	Antibiotic efflux	0	6	0	18	4	0	86	22	0	18	20	64	238
ARO:3002700	cmlv	cmlv is a chromosome-encoded chloramphenicol phoshotransferase that is found in Streptomyces venezuelae	chloramphenicol phosphotransferase	phenicol antibiotic	Phenicol	Antibiotic inactivation	1270	366	298	1650	720	346	2304	1916	666	2074	1692	2900	16202
ARO:3002701	Rhodococcus fascians cmr	cmr is a plasmid-encoded chloramphenicol exporter that is found in Rhodococcus fascians and Corynebacterium glutamicum	major facilitator superfamily (MFS) antibiotic efflux pump	phenicol antibiotic	Phenicol	Antibiotic efflux	8	46	12	24	0	30	16	4	4	28	42	82	296
ARO:3002702	cmrA	cmrA is a transposon-encoded chloramphenicol exporter that is found in Rhodococcus rhodochrous	major facilitator superfamily (MFS) antibiotic efflux pump	phenicol antibiotic	Phenicol	Antibiotic efflux	552	162	140	968	432	86	1382	1054	324	234	246	220	5800

ARO:3002703	cmx	cmx is a plasmid or transposon-encoded chloramphenicol exporter that is found in Corynebacterium striatum and Pseudomonas aeruginosa.	major facilitator superfamily (MFS) antibiotic efflux pump	phenicol antibiotic	Phenicol	Antibiotic efflux	998	1678	844	472	758	1218	950	514	936	3814	5760	4506	22448
ARO:3002704	fexA	fexA is a plasmid-encoded chloramphenicol exporter that is found in Staphylococcus lentus	major facilitator superfamily (MFS) antibiotic efflux pump	phenicol antibiotic	Phenicol	Antibiotic efflux	194	68	96	34	56	370	76	40	114	122	188	152	1510
ARO:3002705	floR	floR is a plasmid or chromosome-encoded chloramphenicol exporter that is found in Bordetella bronchiseptica, Escherichia coli, Klebsiella pneumoniae, Salmonella enterica subsp. enterica serovar Typhimurium str. DT104 and Vibrio cholerae	major facilitator superfamily (MFS) antibiotic efflux pump	phenicol antibiotic	Phenicol	Antibiotic efflux	1870	3448	2890	4404	11560	1978	5540	6484	10168	14728	14542	19346	96958
ARO:3002707	QnrA1	QnrA1 is a plasmid-mediated quinolone resistance protein found in Klebsiella pneumoniae	quinolone resistance protein (qnr)	fluoroquinolone antibiotic	Fluoroquinolone	Antibiotic target protection	0	0	0	0	0	0	0	0	0	32	30	26	88
ARO:3002710	QnrA4	QnrA4 is a plasmid-mediated quinolone resistance protein found in Shewanella algae	quinolone resistance protein (qnr)	fluoroquinolone antibiotic	Fluoroquinolone	Antibiotic target protection	0	0	0	0	0	0	0	0	0	20	30	4	54
ARO:3002715	QnrB2	QnrB2 is a plasmid-mediated quinolone resistance protein found in Citrobacter koseri	quinolone resistance protein (qnr)	fluoroquinolone antibiotic	Fluoroquinolone	Antibiotic target protection	0	0	0	0	0	0	0	0	0	10	4	36	50
ARO:3002724	QnrB10	QnrB10 is a plasmid-mediated quinolone resistance protein found in Citrobacter freundii	quinolone resistance protein (qnr)	fluoroquinolone antibiotic	Fluoroquinolone	Antibiotic target protection	0	0	0	2	0	0	12	4	0	0	2	0	20
ARO:3002734	QnrB19	QnrB19 is a plasmid-mediated quinolone resistance protein found in Escherichia coli	quinolone resistance protein (qnr)	fluoroquinolone antibiotic	Fluoroquinolone	Antibiotic target protection	2	0	0	8	0	0	12	4	0	0	8	0	34
ARO:3002735	QnrB20	QnrB20 is a plasmid-mediated quinolone resistance protein found in Escherichia coli	quinolone resistance protein (qnr)	fluoroquinolone antibiotic	Fluoroquinolone	Antibiotic target protection	0	0	0	4	0	0	10	22	0	0	0	0	36

ARO:3002744	QnrB29	QnrB29 is a plasmid-mediated quinolone resistance protein found in Citrobacter freundii	quinolone resistance protein (qnr)	fluoroquinolone antibiotic	Fluoroquinolone	Antibiotic target protection	0	4	0	0	4	0	0	0	0	6	12	4	30
ARO:3002747	QnrB32	QnrB32 is a plasmid-mediated quinolone resistance protein found in Citrobacter freundii	quinolone resistance protein (qnr)	fluoroquinolone antibiotic	Fluoroquinolone	Antibiotic target protection	0	0	0	4	0	0	20	0	0	2	0	6	32
ARO:3002750	QnrB35	QnrB35 is a plasmid-mediated quinolone resistance protein found in Citrobacter freundii	quinolone resistance protein (qnr)	fluoroquinolone antibiotic	Fluoroquinolone	Antibiotic target protection	0	0	0	0	0	0	0	0	0	0	0	10	10
ARO:3002787	QnrC	QnrC is a plasmid-mediated quinolone resistance protein found in Proteus mirabilis	quinolone resistance protein (qnr)	fluoroquinolone antibiotic	Fluoroquinolone	Antibiotic target protection	12	2	0	0	0	24	4	0	0	0	0	4	46
ARO:3002790	QnrS1	QnrS1 is a plasmid-mediated quinolone resistance protein found in Shigella flexneri	quinolone resistance protein (qnr)	fluoroquinolone antibiotic	Fluoroquinolone	Antibiotic target protection	370	248	90	968	402	46	1180	1046	248	72	108	36	4814
ARO:3002795	QnrS6	QnrS6 is a plasmid-mediated quinolone resistance protein found in Aeromonas hydrophila	quinolone resistance protein (qnr)	fluoroquinolone antibiotic	Fluoroquinolone	Antibiotic target protection	0	0	0	8	0	0	0	14	0	0	0	0	22
ARO:3002799	QnrVC1	QnrVC1 is an integron-mediated quinolone resistance protein found in Vibrio cholerae	quinolone resistance protein (qnr)	fluoroquinolone antibiotic	Fluoroquinolone	Antibiotic target protection	12	0	0	0	0	6	0	0	0	0	0	0	18
ARO:3002800	QnrVC3	QnrVC3 is an integron-mediated quinolone resistance protein found in Vibrio cholerae	quinolone resistance protein (qnr)	fluoroquinolone antibiotic	Fluoroquinolone	Antibiotic target protection	0	0	2	0	0	0	2	0	0	78	68	62	212
ARO:3002801	QnrVC4	QnrVC4 is an integron-mediated quinolone resistance protein found in Aeromonas punctata	quinolone resistance protein (qnr)	fluoroquinolone antibiotic	Fluoroquinolone	Antibiotic target protection	0	0	0	10	0	0	0	30	0	118	198	190	546
ARO:3002802	QnrVC5	QnrVC5 is an integron-mediated quinolone resistance protein found in Vibrio fluvialis	quinolone resistance protein (qnr)	fluoroquinolone antibiotic	Fluoroquinolone	Antibiotic target protection	0	0	8	4	0	0	0	0	2	16	10	16	56
ARO:3002803	QnrVC6	QnrVC6 is an integron-mediated quinolone resistance protein found in Acinetobacter baumannii	quinolone resistance protein (qnr)	fluoroquinolone antibiotic	Fluoroquinolone	Antibiotic target protection	0	0	0	0	0	0	6	0	0	344	282	348	980
ARO:3002812	pp-flo	pp-flo is a plasmid chloramphenicol exporter that	major facilitator superfamily	phenicol antibiotic	Phenicol	Antibiotic efflux	98	110	64	14	92	108	16	22	26	112	228	202	1092

		is found in Photobacterium damselae subsp. piscicida	(MFS) antibiotic efflux pump															
ARO:3002813	lmrB	lmrB is a chromosomally-encoded efflux pump that confers resistance to lincosamides in Bacillus subtilis	ATP-binding cassette (ABC) antibiotic efflux pump	lincosamide antibiotic	MLS	Antibiotic efflux	1160	290	480	258	1476	1376	506	518	1462	956	1510	1232	11224
ARO:3002814	clbA	clbA is a plasmid-encoded cfr gene found in Bacillus velezensis (Bacillus amyloliquefaciens subsp. plantarum).	Cfr 23S ribosomal RNA methyltransferase	lincosamide antibiotic;phenicol antibiotic;pleuromutilin antibiotic;streptogramin antibiotic	Multidrug	Antibiotic target alteration	3234	3578	4176	1816	1180	3656	1732	2198	1642	1710	1266	1570	27758
ARO:3002815	clbB	clbB is a plasmid-encoded cfr gene found in Bacillus brevis	Cfr 23S ribosomal RNA methyltransferase	lincosamide antibiotic;phenicol antibiotic;pleuromutilin antibiotic;streptogramin antibiotic	Multidrug	Antibiotic target alteration	120	226	292	266	86	184	156	230	92	154	176	166	2148
ARO:3002816	clbC	clbC is a plasmid-encoded cfr gene found in Bacillus clausii	Cfr 23S ribosomal RNA methyltransferase	lincosamide antibiotic;phenicol antibiotic;pleuromutilin antibiotic;streptogramin antibiotic	Multidrug	Antibiotic target alteration	726	1048	250	248	88	554	218	274	114	248	422	426	4616
ARO:3002817	carA	carA is an ABC-F subfamily protein involved in macrolide resistance. It is found in Streptomyces thermotolerans	ABC-F ATP-binding cassette ribosomal protection protein	lincosamide antibiotic;macrolide antibiotic;oxazolidinone antibiotic;phenicol antibiotic;pleuromutilin antibiotic;streptogramin antibiotic;tetracycline antibiotic	Multidrug	Antibiotic target protection	2502	2234	2330	2548	2422	2060	3778	2918	2534	4544	4864	5352	38086
ARO:3002819	msrC	msrC is a chromosomal-encoded ABC-F subfamily protein expressed in Enterococcus faecium that confers resistance to erythromycin and other	ABC-F ATP-binding cassette ribosomal	lincosamide antibiotic;macrolide antibiotic;oxazolidinone	Multidrug	Antibiotic target protection	482	156	140	108	86	544	142	144	96	320	326	346	2890

		macrolide and streptogramin B antibiotics.	protection protein	antibiotic;phenicol antibiotic;pleuromutilin antibiotic;streptogramin antibiotic;tetracycline antibiotic															
ARO:3002823	ErmH	ErmH is a plasmid-mediated methyltransferase found in Streptomyces thermotolerans	Erm 23S ribosomal RNA methyltransferase	lincosamide antibiotic;macrolide antibiotic;streptogramin antibiotic	MLS	Antibiotic target alteration	242	100	116	34	10	126	60	24	32	22	44	20	830
ARO:3002824	ErmV	ErmV is a plasmid-mediated methyltransferase found in Streptomyces viridochromogenes	Erm 23S ribosomal RNA methyltransferase	lincosamide antibiotic;macrolide antibiotic;streptogramin antibiotic	MLS	Antibiotic target alteration	0	0	0	2	2	0	10	0	0	28	50	28	120
ARO:3002825	ErmY	ErmY is a plasmid-mediated methyltransferase found in Staphylococcus aureus	Erm 23S ribosomal RNA methyltransferase	lincosamide antibiotic;macrolide antibiotic;streptogramin antibiotic	MLS	Antibiotic target alteration	0	20	0	0	0	0	0	0	0	0	24	4	48
ARO:3002827	tlrC	tlrC is an ABC-F subfamily protein found in Streptomyces fradiae and confers resistance to mycinamicin, tylosin and lincosamides. tlrC is found in the tylosin biosynthetic cluster and is one mechanism by which S. fradiae protects itself from self-destruction when producing this macrolide.	ABC-F ATP-binding cassette ribosomal protection protein	lincosamide antibiotic;macrolide antibiotic;oxazolidinone antibiotic;phenicol antibiotic;pleuromutilin antibiotic;streptogramin antibiotic;tetracycline antibiotic	Multidrug	Antibiotic target protection	840	870	868	610	798	814	968	902	924	2788	2748	3680	16810
ARO:3002828	srmB	srmB is an ABC-F subfamily protein found in Streptomyces ambofaciens that confers resistance to spiramycin	ABC-F ATP-binding cassette ribosomal protection protein	lincosamide antibiotic;macrolide antibiotic;oxazolidinone antibiotic;phenicol antibiotic;pleuromutilin antibiotic;streptogramin	Multidrug	Antibiotic target protection	1168	1610	1554	858	636	1340	1230	1028	1040	1940	2246	1892	16542

ARO:3002829	vgaA	vgaA is an ABC-F subfamily protein expressed in staphylococci that confers resistance to streptogramin A antibiotics and related compounds. It is associated with plasmid DNA.	ABC-F ATP-binding cassette ribosomal protection protein	antibiotic;tetracycline antibiotic lincosamide antibiotic;macrolide antibiotic;oxazolidinone antibiotic;phenicol antibiotic;pleuromutilin antibiotic;streptogramin	Multidrug	Antibiotic target protection	0	44	8	0	0	8	0	0	0	0	2	0	62
ARO:3002830	vgaALC	vgaALC is an ABC-F subfamily protein expressed in staphylococci that confers resistance to streptogramin A antibiotics and related compounds. It is associated with plasmid DNA.	ABC-F ATP-binding cassette ribosomal protection protein	antibiotic;tetracycline antibiotic lincosamide antibiotic;macrolide antibiotic;oxazolidinone antibiotic;phenicol antibiotic;pleuromutilin antibiotic;streptogramin	Multidrug	Antibiotic target protection	256	284	432	202	194	464	184	258	322	166	202	256	3220
ARO:3002831	vgaC	vgaC is an ABC-F subfamily protein expressed in staphylococci that confers resistance to streptogramin A antibiotics and related compounds. It is associated with plasmid DNA.	ABC-F ATP-binding cassette ribosomal protection protein	antibiotic;tetracycline antibiotic lincosamide antibiotic;macrolide antibiotic;oxazolidinone antibiotic;phenicol antibiotic;pleuromutilin antibiotic;streptogramin	Multidrug	Antibiotic target protection	154	408	358	428	1722	238	698	916	1790	1012	1280	982	9986
ARO:3002832	vgaD	vgaD is an ABC-F subfamily protein expressed in Enterococcus faecium that confers resistance to streptogramin A antibiotics and	ABC-F ATP-binding cassette ribosomal protection protein	antibiotic;tetracycline antibiotic lincosamide antibiotic;macrolide antibiotic;oxazolidinone antibiotic;phenic	Multidrug	Antibiotic target protection	314	404	406	290	92	322	224	230	154	316	284	320	3356

		related compounds. It is associated with plasmid DNA.		ol antibiotic;pleuromutilin antibiotic;streptogramin antibiotic;tetracycline antibiotic															
ARO:3002833	vgaE	vgaE is an ABC-F subfamily protein expressed in staphylococci that confers resistance to streptogramin A antibiotics and related compounds. It is associated with transposon DNA.	ABC-F ATP-binding cassette ribosomal protection protein	lincosamide antibiotic;macrolide antibiotic;oxazolidinone antibiotic;phenicol antibiotic;pleuromutilin antibiotic;streptogramin antibiotic;tetracycline antibiotic	Multidrug	Antibiotic target protection	138	156	496	158	118	124	168	174	128	448	406	566	3080
ARO:3002835	lnuA	lnuA is a plasmid-mediated nucleotidyltransferase found in Staphylococcus chromogenes	lincosamide nucleotidyltransferase (LNU)	lincosamide antibiotic	MLS	Antibiotic inactivation	52	22	54	28	0	86	36	36	12	10	32	4	372
ARO:3002837	lnuC	lnuC is a transposon-mediated nucleotidyltransferase found in Streptococcus agalactiae	lincosamide nucleotidyltransferase (LNU)	lincosamide antibiotic	MLS	Antibiotic inactivation	2442	2712	2198	1230	636	2470	1032	1340	862	424	214	148	15708
ARO:3002838	lnuD	lnuD is a plasmid-mediated nucleotidyltransferase found in Streptococcus uberis	lincosamide nucleotidyltransferase (LNU)	lincosamide antibiotic	MLS	Antibiotic inactivation	6	46	8	16	8	12	24	28	16	118	106	82	470
ARO:3002839	lnuF	lnuF is an integron-mediated nucleotidyltransferase found in Escherichia coli	lincosamide nucleotidyltransferase (LNU)	lincosamide antibiotic	MLS	Antibiotic inactivation	14	14	10	88	190	4	124	90	166	566	446	1002	2714
ARO:3002840	vatA	vatA is a plasmid-mediated acetyltransferase found in Staphylococcus aureus	streptogramin vat acetyltransferase	streptogramin antibiotic	MLS	Antibiotic inactivation	36	172	60	22	16	114	20	18	18	0	6	0	482
ARO:3002841	vatB	vatB is a plasmid-mediated acetyltransferase found in Staphylococcus aureus	streptogramin vat acetyltransferase	streptogramin antibiotic	MLS	Antibiotic inactivation	2014	1330	990	608	492	1678	610	776	598	582	572	584	10834
ARO:3002842	vatC	vatC is a plasmid-mediated acetyltransferase found in Staphylococcus cohnii	streptogramin vat	streptogramin antibiotic	MLS	Antibiotic	196	206	144	136	30	140	64	136	34	16	0	4	1106

			acetyltransferase			inactivation													
ARO:3002843	vatD	vatD is a transposon-mediated acetyltransferase found in Enterococcus faecium	streptogramin vat acetyltransferase	streptogramin antibiotic	MLS	Antibiotic inactivation	40	140	248	78	1442	68	46	490	1312	428	344	324	4960
ARO:3002844	vatE	vatE is a transposon-mediated acetyltransferase found in Enterococcus faecium	streptogramin vat acetyltransferase	streptogramin antibiotic	MLS	Antibiotic inactivation	822	262	306	218	60	1210	152	220	176	318	200	226	4170
ARO:3002845	vatH	vatH is a plasmid-mediated acetyltransferase found in Enterococcus faecium	streptogramin vat acetyltransferase	streptogramin antibiotic	MLS	Antibiotic inactivation	468	566	744	310	204	704	264	358	290	448	386	548	5290
ARO:3002846	arr-1	arr-1 is a chromosome-encoded ribosyltransferase found in Mycolicibacterium smegmatis	rifampin ADP-ribosyltransferase (Arr)	rifamycin antibiotic	Rifamycin	Antibiotic inactivation	14	6	2	0	4	10	10	2	0	54	50	60	212
ARO:3002848	arr-3	arr-3 is a plasmid-encoded ribosyltransferase found in Vibrio fluvialis	rifampin ADP-ribosyltransferase (Arr)	rifamycin antibiotic	Rifamycin	Antibiotic inactivation	2	6	6	38	4	6	48	84	24	32	32	46	328
ARO:3002849	arr-4	arr-4 is an integron-encoded ribosyltransferase found in Pseudomonas aeruginosa	rifampin ADP-ribosyltransferase (Arr)	rifamycin antibiotic	Rifamycin	Antibiotic inactivation	0	0	0	2	0	16	10	10	8	10	24	20	100
ARO:3002853	arr-8	arr-8 is an integron-encoded ribosyltransferase found in Klebsiella oxytoca	rifampin ADP-ribosyltransferase (Arr)	rifamycin antibiotic	Rifamycin	Antibiotic inactivation	0	0	0	0	0	0	0	0	0	8	2	2	12
ARO:3002854	dfrA1	dfrA1 is an integron-encoded dihydrofolate reductase	trimethoprim resistant dihydrofolate reductase dfr	diaminopyrimidine antibiotic	Diaminopyrimidine	Antibiotic target replacement	46	116	270	224	1066	58	228	408	1102	2142	1814	2810	10284
ARO:3002857	dfrA26	dfrA26 is an integron-encoded dihydrofolate reductase found in Escherichia coli	trimethoprim resistant dihydrofolate reductase dfr	diaminopyrimidine antibiotic	Diaminopyrimidine	Antibiotic target replacement	510	542	392	268	188	644	302	294	418	1532	1204	2144	8438
ARO:3002858	dfrA12	dfrA12 is an integron-encoded dihydrofolate reductase found in Vibrio cholerae	trimethoprim resistant dihydrofolate reductase dfr	diaminopyrimidine antibiotic	Diaminopyrimidine	Antibiotic target replacement	480	196	174	900	384	56	1212	906	236	200	250	212	5206

ARO:3002859	dfrA14	dfrA14 is an integron-encoded dihydrofolate reductase found in Escherichia coli	trimethoprim resistant dihydrofolate reductase dfr	diaminopyrimidine antibiotic	Diaminopyrimidine	Antibiotic target replacement	12	6	40	106	24	4	94	134	22	100	140	198	880
ARO:3002860	dfrA17	dfrA17 is an integron-encoded dihydrofolate reductase found in Escherichia coli	trimethoprim resistant dihydrofolate reductase dfr	diaminopyrimidine antibiotic	Diaminopyrimidine	Antibiotic target replacement	0	0	24	30	54	0	120	50	46	94	140	204	762
ARO:3002861	dfrA5	dfrA5 is an integron-encoded dihydrofolate reductase found in Vibrio cholerae	trimethoprim resistant dihydrofolate reductase dfr	diaminopyrimidine antibiotic	Diaminopyrimidine	Antibiotic target replacement	0	0	4	0	0	0	14	6	0	14	22	26	86
ARO:3002863	dfrA8	dfrA8 is a transposon-encoded dihydrofolate reductase found in Salmonella enterica	trimethoprim resistant dihydrofolate reductase dfr	diaminopyrimidine antibiotic	Diaminopyrimidine	Antibiotic target replacement	224	54	6	6	2	86	14	10	4	0	2	2	410
ARO:3002864	dfrB1	A plasmid-associated trimethoprim-resistant dihydrofolate reductase detected in Bordetella bronchispetica on pKBB958.	trimethoprim resistant dihydrofolate reductase dfr	diaminopyrimidine antibiotic	Diaminopyrimidine	Antibiotic target replacement	0	0	8	6	0	0	0	0	0	6	6	0	26
ARO:3002865	dfrC	dfrC is a chromosome-encoded dihydrofolate reductase found in Staphylococcus aureus	trimethoprim resistant dihydrofolate reductase dfr	diaminopyrimidine antibiotic	Diaminopyrimidine	Antibiotic target replacement	162	158	284	122	58	274	68	106	146	58	54	28	1518
ARO:3002866	dfrD	dfrD is a plasmid-encoded dihydrofolate reductase found in Listeria monocytogenes	trimethoprim resistant dihydrofolate reductase dfr	diaminopyrimidine antibiotic	Diaminopyrimidine	Antibiotic target replacement	524	488	232	76	78	364	82	92	64	240	198	288	2726
ARO:3002867	dfrF	dfrF is a chromosome-encoded dihydrofolate reductase found in Streptococcus pyogenes	trimethoprim resistant dihydrofolate reductase dfr	diaminopyrimidine antibiotic	Diaminopyrimidine	Antibiotic target replacement	130	232	194	110	166	108	102	150	204	48	36	28	1508
ARO:3002868	dfrG	dfrG is a plasmid-encoded dihydrofolate reductase found in Staphylococcus aureus	trimethoprim resistant dihydrofolate reductase dfr	diaminopyrimidine antibiotic	Diaminopyrimidine	Antibiotic target replacement	106	42	90	42	106	70	48	48	94	160	156	186	1148

ARO:3002869	dfrK	dfrK is a plasmid-encoded dihydrofolate reductase found in Staphylococcus aureus	trimethoprim resistant dihydrofolate reductase dfr	diaminopyrimidine antibiotic	Diaminopyrimidine	Antibiotic target replacement	52	50	44	16	4	2	38	12	6	2	14	20	260
ARO:3002871	tet37	tet37 is a chromosome-encoded oxidoreductase isolated from an uncultured bacterium that confers resistance to tetracycline	tetracycline inactivation enzyme	tetracycline antibiotic	Tetracycline	Antibiotic inactivation	486	704	786	340	184	700	240	324	290	834	532	860	6280
ARO:3002872	FosA3	An enzyme that confers resistance to fosfomycin in Escherichia coli by breaking the epoxide ring of the molecule. It depends on the cofactors Manganese (II) and Potassium and uses Glutathione (GSH) as the nucleophilic molecule.	fosfomycin thiol transferase	fosfomycin	Fosfomycin	Antibiotic inactivation	0	0	0	6	0	0	0	0	0	0	0	0	6
ARO:3002873	FosB3	A thiol transferase that leads to the resistance of fosfomycin in Enterococcus faecium. Contrasting FosA, FosB is dependent on the cofactor Magnesium (II) and uses either bacillithiol to open up the epoxide ring of fosfomycin.	fosfomycin thiol transferase	fosfomycin	Fosfomycin	Antibiotic inactivation	20	36	14	0	0	10	8	2	0	0	0	0	90
ARO:3002874	FosC2	FosC2 is an enzyme that phosphorylates fosfomycin to confer resistance in Escherichia coli	fosC phosphotransferase family	fosfomycin	Fosfomycin	Antibiotic inactivation	18	0	0	0	0	6	16	0	0	26	38	54	158
ARO:3002875	dfrE	dfrE is a chromosome-encoded dihydrofolate reductase found in Enterococcus faecalis	trimethoprim resistant dihydrofolate reductase dfr	diaminopyrimidine antibiotic	Diaminopyrimidine	Antibiotic target replacement	246	164	46	20	8	454	8	16	36	56	36	26	1116
ARO:3002878	BcII	Bacillus cereus beta-lactamase II is a zinc metallo-beta-lactamase that hydrolyzes a large number of penicillins and cephalosporins in the Bacillus cereus strain 5/B/6	Bc beta-lactamase	cephalosporin;penam	Beta-lactam	Antibiotic inactivation	2	0	2	0	4	2	0	0	20	2	20	14	66
ARO:3002881	lmrC	lmrC is an ABC-F subfamily protein that confers resistance to lincosamides in Streptomyces lincolnensis and Lactococcus lactis. It can dimerize with lmrD	ABC-F ATP-binding cassette ribosomal protection protein	lincosamide antibiotic;macrolide antibiotic;oxazolidinone antibiotic;phenic	Multidrug	Antibiotic target protection	4322	6276	8288	4200	3030	5572	4014	4590	4128	7822	8596	8168	69006

				ol antibiotic;pleuromutilin antibiotic;streptogramin antibiotic;tetracycline antibiotic														
ARO:3002882	lmrD	lmrD is a chromosomally-encoded efflux pump that confers resistance to lincosamides in Streptomyces lincolnensis and Lactococcus lactis. It can dimerize with lmrC	ATP-binding cassette (ABC) antibiotic efflux pump	lincosamide antibiotic	MLS	Antibiotic efflux	9104	8998	6370	8428	7584	8836	10484	9454	7740	4716	4606	4780	91100
ARO:3002883	rgt1438	rgt1438 is a glycosyltransferase that confers rifampin resistance in Streptomyces	rifampin glycosyltransferase	rifamycin antibiotic	Rifamycin	Antibiotic inactivation	248	46	40	42	18	152	132	70	8	6	18	8	788
ARO:3002884	iri	iri is a monooxygenase that confers resistance to rifampin found in Rhodococcus hoagii	rifampin monooxygenase	rifamycin antibiotic	Rifamycin	Antibiotic inactivation	1032	1604	328	1256	600	770	2026	1288	566	594	712	864	11640
ARO:3002891	otr(A)	otr(A) is an oxytetracycline resistance ribosomal protection protein found in Streptomyces rimosus	tetracycline-resistant ribosomal protection protein	tetracycline antibiotic	Tetracycline	Antibiotic target protection	5326	5354	5714	3892	4994	5398	4598	4274	4930	5994	6594	6392	63460
ARO:3002892	otr(B)	otr(B) is a tetracycline resistance efflux pump found in Streptomyces rimosus	major facilitator superfamily (MFS) antibiotic efflux pump	tetracycline antibiotic	Tetracycline	Antibiotic efflux	178	16	26	90	48	94	258	104	64	710	1040	928	3556
ARO:3002893	tcr3	tcr3 is a tetracycline efflux pump that confers self-resistance to Kitasatospora aureofaciens	major facilitator superfamily (MFS) antibiotic efflux pump	tetracycline antibiotic	Tetracycline	Antibiotic efflux	582	178	386	1142	2200	78	1612	1462	1724	826	690	748	11628
ARO:3002894	otrC	otrC is a tetracycline resistance efflux pump found in Streptomyces rimosus	ATP-binding cassette (ABC) antibiotic efflux pump	tetracycline antibiotic	Tetracycline	Antibiotic efflux	290	378	202	100	110	272	120	138	134	230	268	222	2464
ARO:3002895	SAT-2	SAT-2 is a plasmid-mediated streptothricin acetyltransferase, which confers resistance to streptothricin, a nucleoside	streptothricin acetyltransferase (SAT)	nucleoside antibiotic	Nucleoside	Antibiotic inactivation	124	302	58	114	110	162	92	86	84	106	94	182	1514

ARO:3002897	SAT-4	antibiotic. Originally described from an E. coli plasmid sequence by Heim et al., 1989. SAT-4 is a plasmid-mediated streptothricin acetyltransferase and streptothricin (a nucleoside antibiotic) resistant determinant. Originally described from a Campylobacter coli BE/G4 plasmid gene sequence by Jacob et al, 1994.	streptothricin acetyltransferase (SAT)	nucleoside antibiotic	Nucleoside	Antibiotic inactivation	690	348	910	436	354	438	374	430	452	248	240	174	5094
ARO:3002898	SAT-3	SAT-3 is a plasmid-mediated streptothricin acetyltransferase and streptothricin resistance determinant. Originally described from an E. coli plasmid gene by Tietze and Brevet, 1995.	streptothricin acetyltransferase (SAT)	nucleoside antibiotic	Nucleoside	Antibiotic inactivation	0	16	4	0	0	12	2	4	16	0	6	2	62
ARO:3002907	vanE	VanE is a D-Ala-D-Ala ligase homolog that can synthesize D-Ala-D-Ser, an alternative substrate for peptidoglycan synthesis that reduces vancomycin binding affinity in Enterococcus faecalis	glycopeptide resistance gene cluster;van ligase	glycopeptide antibiotic	Glycopeptide	Antibiotic target alteration	732	752	844	376	246	956	286	388	424	612	622	662	6900
ARO:3002908	vanF	VanF is a D-Ala-D-Ala ligase homolog that can synthesize D-Ala-D-Lac, an alternative substrate for peptidoglycan synthesis that reduces vancomycin binding affinity. It is associated with both vancomycin and teicoplanin resistance in Paenibacillus popilliae	glycopeptide resistance gene cluster;van ligase	glycopeptide antibiotic	Glycopeptide	Antibiotic target alteration	42	56	124	110	22	114	86	160	74	468	312	332	1900
ARO:3002909	vanG	VanG is a D-Ala-D-Ala ligase homolog that can synthesize D-Ala-D-Ser, an alternative substrate for peptidoglycan synthesis that reduces vancomycin binding affinity in Enterococcus faecalis	glycopeptide resistance gene cluster;van ligase	glycopeptide antibiotic	Glycopeptide	Antibiotic target alteration	2260	2438	1204	2550	1398	1692	3052	2616	1342	1006	1050	872	21480
ARO:3002910	vanL	VanL is a D-Ala-D-Ala ligase homolog that can synthesize D-Ala-D-Ser, an alternative substrate for peptidoglycan synthesis that reduces	glycopeptide resistance gene cluster;van ligase	glycopeptide antibiotic	Glycopeptide	Antibiotic target alteration	1166	512	558	818	450	716	1284	1078	396	228	266	142	7614

		vancomycin binding affinity in Enterococcus faecalis																	
ARO:3002911	vanM	VanM is a D-Ala-D-Ala ligase homolog that can synthesize D-Ala-D-Lac, an alternative substrate for peptidoglycan synthesis that reduces vancomycin binding affinity. It is associated with both vancomycin and teicoplanin resistance.	glycopeptide resistance gene cluster;van ligase	glycopeptide antibiotic	Glycopeptide	Antibiotic target alteration	118	304	198	76	18	242	92	78	40	154	268	158	1746
ARO:3002912	vanN	VanN is a D-Ala-D-Ala ligase homolog that can synthesize D-Ala-D-Ser, an alternative substrate for peptidoglycan synthesis that reduces vancomycin binding affinity in Enterococcus faecium	glycopeptide resistance gene cluster;van ligase	glycopeptide antibiotic	Glycopeptide	Antibiotic target alteration	502	614	1344	210	420	464	212	210	724	1106	850	1570	8226
ARO:3002913	vanO	VanO is a D-Ala-D-Ala ligase homolog that can synthesize D-Ala-D-Lac, an alternative substrate for peptidoglycan synthesis that reduces vancomycin binding affinity. It is associated with both vancomycin and teicoplanin resistance.	glycopeptide resistance gene cluster;van ligase	glycopeptide antibiotic	Glycopeptide	Antibiotic target alteration	108	100	168	126	80	138	118	100	90	54	98	90	1270
ARO:3002919	vanRA	vanRA, also known as vanR, is a vanR variant found in the vanA gene cluster	glycopeptide resistance gene cluster;vanR	glycopeptide antibiotic	Glycopeptide	Antibiotic target alteration	1560	1256	1108	1596	866	968	2196	1726	930	472	398	218	13294
ARO:3002921	vanRB	vanRB is a vanR variant found in the vanB gene cluster	glycopeptide resistance gene cluster;vanR	glycopeptide antibiotic	Glycopeptide	Antibiotic target alteration	1676	1488	1652	1568	1044	1576	1836	1568	1176	3748	3164	5180	25676
ARO:3002922	vanRC	vanRC is a vanR variant found in the vanC gene cluster	glycopeptide resistance gene cluster;vanR	glycopeptide antibiotic	Glycopeptide	Antibiotic target alteration	438	102	226	72	74	290	66	156	138	194	168	300	2224
ARO:3002923	vanRD	vanRD is a mutated vanR variant found in the vanD gene cluster that caused constitutive expression of vanD peptidoglycan synthesis	glycopeptide resistance gene cluster;vanR	glycopeptide antibiotic	Glycopeptide	Antibiotic target alteration	194	66	154	40	70	152	56	40	72	58	106	68	1076
ARO:3002924	vanRE	vanRE is a vanR variant found in the vanE gene cluster	glycopeptide resistance	glycopeptide antibiotic	Glycopeptide	Antibiotic target	1732	904	656	654	584	1458	564	944	798	738	654	498	10184

			gene cluster;vanR			alteration												
ARO:3002925	vanRF	vanRF is a vanR variant found in the vanF gene cluster	glycopeptide resistance gene cluster;vanR	glycopeptide antibiotic	Glycopeptide	Antibiotic target alteration	8216	7794	6122	3502	8104	7234	3604	5234	8908	10510	9236	12420	90884
ARO:3002926	vanRG	vanRG is a vanR variant found in the vanG gene cluster	glycopeptide resistance gene cluster;vanR	glycopeptide antibiotic	Glycopeptide	Antibiotic target alteration	1216	1052	884	470	496	1050	418	500	566	710	576	932	8870
ARO:3002927	vanRL	vanRL is a vanR variant found in the vanL gene cluster	glycopeptide resistance gene cluster;vanR	glycopeptide antibiotic	Glycopeptide	Antibiotic target alteration	96	202	120	156	112	96	172	180	104	716	652	780	3386
ARO:3002928	vanRM	vanRM is a vanR variant found in the vanM gene cluster	glycopeptide resistance gene cluster;vanR	glycopeptide antibiotic	Glycopeptide	Antibiotic target alteration	3332	2582	2058	1386	1158	3456	1396	1530	1380	2692	2684	3386	27040
ARO:3002929	vanRN	vanRN is a vanR variant found in the vanN gene cluster	glycopeptide resistance gene cluster;vanR	glycopeptide antibiotic	Glycopeptide	Antibiotic target alteration	102	216	388	142	80	204	220	128	140	164	106	62	1952
ARO:3002930	vanRO	vanRO is a vanR variant found in the vanO gene cluster	glycopeptide resistance gene cluster;vanR	glycopeptide antibiotic	Glycopeptide	Antibiotic target alteration	282	738	1468	1076	6974	478	1514	2192	6908	3380	3266	3298	31574
ARO:3002931	vanSA	vanSA, also known as vanS, is a vanS variant found in the vanA gene cluster	glycopeptide resistance gene cluster;vanS	glycopeptide antibiotic	Glycopeptide	Antibiotic target alteration	1890	1724	1588	886	1436	1612	1218	1238	1316	1940	2000	2430	19278
ARO:3002932	vanSB	vanSB is a vanS variant found in the vanB gene cluster	glycopeptide resistance gene cluster;vanS	glycopeptide antibiotic	Glycopeptide	Antibiotic target alteration	1432	2170	440	1554	628	1172	2058	1472	560	876	908	1142	14412
ARO:3002933	vanSC	vanSC is a vanS variant found in the vanC gene cluster	glycopeptide resistance gene cluster;vanS	glycopeptide antibiotic	Glycopeptide	Antibiotic target alteration	266	726	356	240	180	384	144	188	490	378	408	400	4160
ARO:3002934	vanSD	vanSD is a mutated vanS variant found in the vanD gene cluster that caused constitutive expression of vanD peptidoglycan synthesis	glycopeptide resistance gene cluster;vanS	glycopeptide antibiotic	Glycopeptide	Antibiotic target alteration	4	32	42	10	12	20	14	14	4	8	26	8	194
ARO:3002935	vanSE	vanSE is a vanS variant found in the vanE gene cluster	glycopeptide resistance gene cluster;vanS	glycopeptide antibiotic	Glycopeptide	Antibiotic target alteration	1654	1286	1078	538	342	1250	560	656	448	738	692	810	10052

ARO:3002936	vanSF	vanSF is a vanS variant found in the vanF gene cluster	glycopeptide resistance gene cluster;vanS	glycopeptide antibiotic	Glycopeptide	Antibiotic target alteration	2002	2138	1078	780	524	2056	658	816	706	1388	1224	1546	14916
ARO:3002937	vanSG	vanSG is a vanS variant found in the vanG gene cluster	glycopeptide resistance gene cluster;vanS	glycopeptide antibiotic	Glycopeptide	Antibiotic target alteration	4186	4656	4932	3244	3282	3490	4410	3872	3882	2776	2396	2722	43848
ARO:3002938	vanSL	vanSL is a vanS variant found in the vanL gene cluster	glycopeptide resistance gene cluster;vanS	glycopeptide antibiotic	Glycopeptide	Antibiotic target alteration	596	450	248	1034	546	254	1298	1042	410	260	344	216	6698
ARO:3002939	vanSM	vanSM is a vanS variant found in the vanM gene cluster	glycopeptide resistance gene cluster;vanS	glycopeptide antibiotic	Glycopeptide	Antibiotic target alteration	2422	2974	1898	1762	858	1868	2174	2060	1182	764	570	540	19072
ARO:3002940	vanSN	vanSN is a vanS variant found in the vanN gene cluster	glycopeptide resistance gene cluster;vanS	glycopeptide antibiotic	Glycopeptide	Antibiotic target alteration	510	890	1458	930	5960	976	1372	1768	5946	5650	4708	6368	36536
ARO:3002941	vanSO	vanSO is a vanS variant found in the vanO gene cluster	glycopeptide resistance gene cluster;vanS	glycopeptide antibiotic	Glycopeptide	Antibiotic target alteration	266	444	976	382	348	554	554	450	492	1024	1220	1288	7998
ARO:3002942	vanHA	vanHA, also known as vanH, is a vanH variant in the vanA gene cluster	glycopeptide resistance gene cluster;vanH	glycopeptide antibiotic	Glycopeptide	Antibiotic target alteration	1796	1724	1886	560	552	2222	570	654	1028	570	656	532	12750
ARO:3002943	vanHB	vanHB is a vanH variant in the vanB gene cluster	glycopeptide resistance gene cluster;vanH	glycopeptide antibiotic	Glycopeptide	Antibiotic target alteration	1692	2516	1626	1286	646	1922	1312	1228	864	4208	4108	6280	27688
ARO:3002944	vanHD	vanHD is a vanH variant in the vanD gene cluster	glycopeptide resistance gene cluster;vanH	glycopeptide antibiotic	Glycopeptide	Antibiotic target alteration	3244	3340	2766	2868	2986	2648	3346	3064	3054	3502	3160	4138	38116
ARO:3002945	vanHF	vanHF is a vanH variant in the vanF gene cluster	glycopeptide resistance gene cluster;vanH	glycopeptide antibiotic	Glycopeptide	Antibiotic target alteration	1530	2738	2366	1212	5150	2072	1434	1856	4484	2786	2722	3100	31450
ARO:3002947	vanHM	vanHM is a vanH variant in the vanM gene cluster	glycopeptide resistance gene cluster;vanH	glycopeptide antibiotic	Glycopeptide	Antibiotic target alteration	1348	1412	1878	676	802	1116	688	782	1044	414	390	294	10844
ARO:3002948	vanHO	vanHO is a vanH variant in the vanO gene cluster	glycopeptide resistance	glycopeptide antibiotic	Glycopeptide	Antibiotic target	3414	2690	2056	2032	1364	3880	2578	2170	1396	2110	2070	2462	28222

			gene cluster;vanH			alteration												
ARO:3002949	vanXA	vanXA, also known as vanX, is a vanX variant found in the vanA gene cluster	glycopeptide resistance gene cluster;vanX	glycopeptide antibiotic	Glycopeptide	Antibiotic target alteration	6	30	4	24	4	4	18	12	8	0	0	0	110
ARO:3002950	vanXB	vanXB is a vanX variant found in the vanB gene cluster	glycopeptide resistance gene cluster;vanX	glycopeptide antibiotic	Glycopeptide	Antibiotic target alteration	192	124	80	24	28	196	26	52	42	8	16	14	802
ARO:3002952	vanXF	vanXF is a vanX variant found in the vanF gene cluster	glycopeptide resistance gene cluster;vanX	glycopeptide antibiotic	Glycopeptide	Antibiotic target alteration	0	0	0	8	0	0	40	12	0	16	4	18	98
ARO:3002953	vanXM	vanXM is a vanX variant found in the vanM gene cluster	glycopeptide resistance gene cluster;vanX	glycopeptide antibiotic	Glycopeptide	Antibiotic target alteration	104	418	220	110	88	250	124	106	122	46	64	12	1664
ARO:3002954	vanXO	vanXO is a vanX variant found in the vanO gene cluster	glycopeptide resistance gene cluster;vanX	glycopeptide antibiotic	Glycopeptide	Antibiotic target alteration	398	122	250	228	86	218	288	224	144	94	126	50	2228
ARO:3002955	vanYA	vanYA, also known as vanY, is a vanY variant found in the vanA gene cluster	glycopeptide resistance gene cluster;vanY	glycopeptide antibiotic	Glycopeptide	Antibiotic target alteration	430	356	170	74	50	178	68	36	70	68	78	60	1638
ARO:3002956	vanYB	vanYB is a vanY variant found in the vanB gene cluster	glycopeptide resistance gene cluster;vanY	glycopeptide antibiotic	Glycopeptide	Antibiotic target alteration	366	1326	1426	626	378	738	542	584	498	296	180	154	7114
ARO:3002958	vanYF	vanYF is a vanY variant found in the vanF gene cluster	glycopeptide resistance gene cluster;vanY	glycopeptide antibiotic	Glycopeptide	Antibiotic target alteration	202	468	194	160	86	176	220	214	120	128	50	42	2060
ARO:3002959	vanYG1	vanYG1 is a vanY variant found in the vanG gene cluster	glycopeptide resistance gene cluster;vanY	glycopeptide antibiotic	Glycopeptide	Antibiotic target alteration	12	102	168	40	48	66	46	46	48	18	20	0	614
ARO:3002961	vanYM	vanYM is a vanY variant found in the vanM gene cluster	glycopeptide resistance gene cluster;vanY	glycopeptide antibiotic	Glycopeptide	Antibiotic target alteration	26	182	166	152	40	90	150	156	38	36	26	24	1086
ARO:3002962	vanZA	vanZA, also known as vanZ, is a vanZ variant found in the vanA gene cluster	glycopeptide resistance gene cluster;vanZ	glycopeptide antibiotic	Glycopeptide	Antibiotic target alteration	46	6	10	6	14	22	10	4	28	10	12	20	188

ARO:3002963	vanZF	vanZF is a vanZ variant found in the vanF gene cluster	glycopeptide resistance gene cluster;vanZ	glycopeptide antibiotic	Glycopeptide	Antibiotic target alteration	586	118	92	66	30	490	108	154	84	48	66	46	1888
ARO:3002964	vanWB	vanWB, also known as vanW, is a vanW variant found in the vanB gene cluster	glycopeptide resistance gene cluster;vanW	glycopeptide antibiotic	Glycopeptide	Antibiotic target alteration	234	362	392	266	124	316	190	294	106	66	98	66	2514
ARO:3002965	vanWG	vanWG is a vanW variant found in the vanG gene cluster	glycopeptide resistance gene cluster;vanW	glycopeptide antibiotic	Glycopeptide	Antibiotic target alteration	230	364	220	194	206	280	118	208	228	152	78	50	2328
ARO:3002966	vanXYC	vanXYC is a vanXY variant found in the vanC gene cluster	glycopeptide resistance gene cluster;vanXY	glycopeptide antibiotic	Glycopeptide	Antibiotic target alteration	74	6	0	2	2	16	12	4	0	4	18	0	138
ARO:3002967	vanXYE	vanXYE is a vanXY variant found in the vanE gene cluster	glycopeptide resistance gene cluster;vanXY	glycopeptide antibiotic	Glycopeptide	Antibiotic target alteration	316	124	86	20	76	108	30	24	72	76	32	28	992
ARO:3002968	vanXYL	vanXYL is a vanXY variant found in the vanL gene cluster	glycopeptide resistance gene cluster;vanXY	glycopeptide antibiotic	Glycopeptide	Antibiotic target alteration	46	48	196	80	18	52	80	56	54	14	24	8	676
ARO:3002969	vanXYN	vanXYN is a vanXY variant found in the vanN gene cluster	glycopeptide resistance gene cluster;vanXY	glycopeptide antibiotic	Glycopeptide	Antibiotic target alteration	66	110	338	188	62	148	134	146	72	274	280	290	2108
ARO:3002970	vanTC	vanTC is a vanT variant found in the vanC gene cluster	glycopeptide resistance gene cluster;vanT	glycopeptide antibiotic	Glycopeptide	Antibiotic target alteration	1102	852	860	514	308	1348	450	660	462	676	740	698	8670
ARO:3002971	vanTE	vanTE is a vanT variant found in the vanE gene cluster	glycopeptide resistance gene cluster;vanT	glycopeptide antibiotic	Glycopeptide	Antibiotic target alteration	436	990	1146	138	396	720	166	106	398	362	500	516	5874
ARO:3002972	vanTG	vanTG is a vanT variant found in the vanG gene cluster	glycopeptide resistance gene cluster;vanT	glycopeptide antibiotic	Glycopeptide	Antibiotic target alteration	3112	4972	3218	1782	1542	3772	1252	1744	1594	2176	2038	2388	29590

ARO:3002974	vanTrL	vanTrL is a vanT variant found in the vanL gene cluster. vanTrL codes for the racemase component of vanT	glycopeptide resistance gene cluster;vanT	glycopeptide antibiotic	Glycopeptide	Antibiotic target alteration	116	282	756	820	5318	172	1056	1520	4630	1656	1626	1454	19406
ARO:3002975	vanTN	vanTN is a vanT variant found in the vanN gene cluster	glycopeptide resistance gene cluster;vanT	glycopeptide antibiotic	Glycopeptide	Antibiotic target alteration	470	522	340	132	84	694	276	138	126	234	200	204	3420
ARO:3002982	amrA	amrA is the efflux pump subunit of the AmrAB-OprM multidrug efflux complex. amrA corresponds to 1 locus in Pseudomonas aeruginosa PAO1 and 1 locus in Pseudomonas aeruginosa LESB58.	resistance-nodulation-cell division (RND) antibiotic efflux pump	aminoglycoside antibiotic	Multidrug	Antibiotic efflux	2	156	38	20	26	40	22	30	28	38	26	38	464
ARO:3002983	amrB	amrB is the membrane fusion protein of the AmrAB-OprM multidrug efflux complex.	resistance-nodulation-cell division (RND) antibiotic efflux pump	aminoglycoside antibiotic	Multidrug	Antibiotic efflux	12	16	34	18	56	18	28	22	30	226	270	130	860
ARO:3002985	arnA	arnA modifies lipid A with 4-amino-4-deoxy-L-arabinose (Ara4N) which allows gram-negative bacteria to resist the antimicrobial activity of cationic antimicrobial peptides and antibiotics such as polymyxin. arnA is found in E. coli and P. aeruginosa.	pmr phosphoethanolamine transferase	peptide antibiotic	Peptide	Antibiotic target alteration	3380	4130	3342	2808	1404	4016	2856	2938	1858	2136	1806	2246	32920
ARO:3002986	bacA	The bacA gene product (BacA) recycles undecaprenyl pyrophosphate during cell wall biosynthesis which confers resistance to bacitracin.	undecaprenyl pyrophosphate related proteins	peptide antibiotic	Peptide	Antibiotic target alteration	4312	6050	5260	3402	2732	4930	4152	3852	3250	5068	4834	5882	53724
ARO:3002987	bcrA	bcrA is an ABC transporter found in Bacillus licheniformis that confers bacitracin resistance	ATP-binding cassette (ABC) antibiotic efflux pump	peptide antibiotic	Peptide	Antibiotic efflux	22656	18684	19686	13056	14480	23500	15208	14622	15230	14496	14500	14264	200382
ARO:3002988	bcrB	bcrB is an ABC transporter found in Bacillus licheniformis that confers bacitracin resistance	ATP-binding cassette (ABC) antibiotic efflux pump	peptide antibiotic	Peptide	Antibiotic efflux	174	178	138	60	38	232	28	56	28	8	2	20	962

ARO:3002993	AQU-1	AQU-1 is a chromosomal class C beta-lactamase found in clinical Aeromonas dhakensis isolates	AQU beta-lactamase	cephalosporin	Beta-lactam	Antibiotic inactivation	158	220	128	86	98	198	66	110	146	28	12	14	1264
ARO:3002999	CblA-1	CblA-1 beta-lactamase is a class A beta-lactamase found in Bacteroides uniformis that is species-specific.	CblA beta-lactamase	cephalosporin	Beta-lactam	Antibiotic inactivation	38	72	20	66	4	82	30	102	16	4	2	0	436
ARO:3003002	CfxA2	cfxA2 beta-lactamase is a class A beta-lactamase found in Prevotella intermedia	CfxA beta-lactamase	cephamycin	Beta-lactam	Antibiotic inactivation	132	64	134	68	16	162	52	72	40	22	4	4	770
ARO:3003003	CfxA3	cfxA3 beta-lactamase is a class A beta-lactamase found in Capnocytophaga ochracea	CfxA beta-lactamase	cephamycin	Beta-lactam	Antibiotic inactivation	414	570	898	248	202	544	194	336	350	48	34	36	3874
ARO:3003006	blt	blt is an MFS efflux pump that confers resistance to multiple drugs such as rhodamine and acridine dyes, and fluoroquinolone antibiotics	major facilitator superfamily (MFS) antibiotic efflux pump	acridine dye;fluoroquinolone antibiotic	Multidrug	Antibiotic efflux	190	210	390	40	194	174	40	42	188	1776	1346	2788	7378
ARO:3003007	bmr	bmr is an MFS antibiotic efflux pump that confers resistance to multiple drugs including acridine dyes, fluoroquinolone antibiotics, chloramphenicol, and puromycin	major facilitator superfamily (MFS) antibiotic efflux pump	acridine dye;fluoroquinolone antibiotic;nucleoside antibiotic;phenicol antibiotic	Multidrug	Antibiotic efflux	122	78	6	2	4	296	2	24	6	54	102	84	780
ARO:3003009	ceoA	ceoA is a periplasmic linker subunit of the CeoAB-OpcM efflux pump	resistance-nodulation-cell division (RND) antibiotic efflux pump	aminoglycoside antibiotic;fluoroquinolone antibiotic	Multidrug	Antibiotic efflux	516	228	172	1014	482	120	1302	1022	332	122	158	52	5520
ARO:3003010	ceoB	ceoB is a cytoplasmic membrane component of the CeoAB-OpcM efflux pump	resistance-nodulation-cell division (RND) antibiotic efflux pump	aminoglycoside antibiotic;fluoroquinolone antibiotic	Multidrug	Antibiotic efflux	714	930	1024	926	414	856	612	914	452	288	332	272	7734
ARO:3003013	dfrA15	dfrA15 is an integron-encoded dihydrofolate reductase found in Vibrio cholerae	trimethoprim resistant dihydrofolate reductase dfr	diaminopyrimidine antibiotic	Diaminopyrimidine	Antibiotic target replacement	0	2	22	22	148	0	56	20	184	176	166	236	1032

ARO:3003014	dfrA16	dfrA16 is an integron-encoded dihydrofolate reductase found in Salmonella enterica	trimethoprim resistant dihydrofolate reductase dfr	diaminopyrimidine antibiotic	Diaminopyrimidine	Antibiotic target replacement	20	292	126	148	724	36	214	166	730	440	416	478	3790
ARO:3003016	dfrA20	dfrA20 is a plasmid-encoded dihydrofolate reductase found in Pasteurella multocida	trimethoprim resistant dihydrofolate reductase dfr	diaminopyrimidine antibiotic	Diaminopyrimidine	Antibiotic target replacement	152	276	190	120	44	166	134	112	92	696	778	850	3610
ARO:3003017	dfrA21	dfrA21 is an integron-encoded dihydrofolate reductase found in Salmonella enterica	trimethoprim resistant dihydrofolate reductase dfr	diaminopyrimidine antibiotic	Diaminopyrimidine	Antibiotic target replacement	0	0	0	0	0	0	0	0	0	22	22	18	62
ARO:3003018	dfrA22	dfrA22 is an integron-encoded dihydrofolate reductase found in Salmonella enterica	trimethoprim resistant dihydrofolate reductase dfr	diaminopyrimidine antibiotic	Diaminopyrimidine	Antibiotic target replacement	4	18	26	24	4	12	32	16	14	32	64	52	298
ARO:3003020	dfrA25	dfrA25 is an integron-encoded dihydrofolate reductase found in Salmonella agona	trimethoprim resistant dihydrofolate reductase dfr	diaminopyrimidine antibiotic	Diaminopyrimidine	Antibiotic target replacement	0	0	0	2	0	0	12	12	0	0	0	0	26
ARO:3003021	dfrB2	dfrB2 is an integron-encoded dihydrofolate reductase found in an uncultured bacterium from a wastewater treatment plant	trimethoprim resistant dihydrofolate reductase dfr	diaminopyrimidine antibiotic	Diaminopyrimidine	Antibiotic target replacement	0	0	0	0	0	0	0	2	0	0	8	0	10
ARO:3003022	dfrB3	dfrB3 is an integron-encoded dihydrofolate reductase found in Klebsiella oxytoca	trimethoprim resistant dihydrofolate reductase dfr	diaminopyrimidine antibiotic	Diaminopyrimidine	Antibiotic target replacement	0	0	0	0	0	0	0	0	0	0	8	6	14
ARO:3003023	dfrB6	dfrB6 is an integron-encoded dihydrofolate reductase found in Salmonella enterica	trimethoprim resistant dihydrofolate reductase dfr	diaminopyrimidine antibiotic	Diaminopyrimidine	Antibiotic target replacement	0	0	0	0	0	0	0	0	0	12	12	30	54
ARO:3003030	MexV	MexV is the membrane fusion protein of the MexVW-OprM multidrug efflux complex.	resistance-nodulation-cell division (RND) antibiotic efflux pump	acridine dye;fluoroquinolone antibiotic;macrolide antibiotic;phenicol	Multidrug	Antibiotic efflux	1066	564	474	2056	902	112	3176	2228	912	2808	3168	3446	20912

				antibiotic;tetracycline antibiotic															
ARO:3003031	MexW	MexW is the RND-type membrane protein of the efflux complex MexVW-OprM.	resistance-nodulation-cell division (RND) antibiotic efflux pump	acridine dye;fluoroquinolone antibiotic;macrolide antibiotic;phenicol antibiotic;tetracycline antibiotic	Multidrug	Antibiotic efflux	2076	1358	3446	876	1222	1426	956	1062	1136	6092	6620	7864	34134
ARO:3003033	mexY	MexY is the RND-type membrane protein of the efflux complex MexXY-OprM.	resistance-nodulation-cell division (RND) antibiotic efflux pump	acridine dye;aminoglycoside antibiotic;carbapenem;cephalosporin;cephamycin;fluoroquinolone antibiotic;macrolide antibiotic;penam;phenicol antibiotic;tetracycline antibiotic	Multidrug	Antibiotic efflux	52	58	80	28	32	108	32	42	50	290	264	556	1592
ARO:3003034	mexX	MexX is the membrane fusion protein of the MexXY-OprM multidrug efflux complex.	resistance-nodulation-cell division (RND) antibiotic efflux pump	acridine dye;aminoglycoside antibiotic;carbapenem;cephalosporin;cephamycin;fluoroquinolone antibiotic;macrolide antibiotic;penam;phenicol antibiotic;tetracycline antibiotic	Multidrug	Antibiotic efflux	0	0	2	4	4	0	4	10	2	0	4	4	34
ARO:3003035	mfpA	mfpA is a qnr homolog and a pentapeptide repeat protein that confers resistance to fluoroquinolones in Mycolicibacterium smegmatis	quinolone resistance protein (qnr)	fluoroquinolone antibiotic	Fluoroquinolone	Antibiotic target protection	0	8	16	6	0	0	24	4	6	176	164	36	440
ARO:3003036	oleB	oleB is an ABC-F subfamily protein in Streptomyces antibioticus and is involved in oleandomycin secretion	ABC-F ATP-binding cassette ribosomal	lincosamide antibiotic;macrolide antibiotic;oxazolidinone	Multidrug	Antibiotic target protection	566	780	1118	978	2668	828	1722	1402	3072	3382	4682	3880	25078

			protection protein	antibiotic;phenicol antibiotic;pleuromutilin antibiotic;streptogramin antibiotic;tetracycline antibiotic															
ARO:3003037	opcM	OpcM is an outer membrane factor protein found in Burkholderia cepacia. It is part of the CeoAB-OpcM complex.	resistance-nodulation-cell division (RND) antibiotic efflux pump	aminoglycoside antibiotic;fluoroquinolone antibiotic	Multidrug	Antibiotic efflux	222	72	180	70	54	116	50	74	28	76	124	74	1140
ARO:3003039	OprA	OprA is an outer membrane factor protein found in Pseudomonas aeruginosa. It is part of the MexXY-OprA complex.	resistance-nodulation-cell division (RND) antibiotic efflux pump	aminoglycoside antibiotic;carbapenem;fluoroquinolone antibiotic;macrolide antibiotic;tetracycline antibiotic	Multidrug	Antibiotic efflux	628	308	234	784	288	234	1146	814	268	508	500	402	6114
ARO:3003046	qacA	qacA is a subunit of the qac multidrug efflux pump	major facilitator superfamily (MFS) antibiotic efflux pump	fluoroquinolone antibiotic	Multidrug	Antibiotic efflux	646	24	38	92	26	556	88	100	208	42	60	24	1904
ARO:3003047	qacB	qacB is a subunit of the qac multidrug efflux pump	major facilitator superfamily (MFS) antibiotic efflux pump	fluoroquinolone antibiotic	Multidrug	Antibiotic efflux	0	0	4	4	0	0	18	2	2	64	94	98	286
ARO:3003048	rosA	rosA is part of an efflux pump/potassium antiporter system (RosAB) in Yersinia that confers resistance to cationic antimicrobial peptides such as polymyxin B.	major facilitator superfamily (MFS) antibiotic efflux pump	peptide antibiotic	Peptide	Antibiotic efflux	714	286	238	900	492	378	1452	1204	556	628	700	632	8180
ARO:3003049	rosB	rosB is part of an efflux pump/potassium antiporter system (RosAB) in Yersinia that confers resistance to cationic antimicrobial peptides such as polymyxin B.	major facilitator superfamily (MFS) antibiotic efflux pump	peptide antibiotic	Peptide	Antibiotic efflux	886	840	446	1548	650	664	2224	1648	616	452	530	522	11026

ARO:3003051	smeA	smeA is the membrane fusion protein of the smeABC multidrug efflux complex in Stenotrophomonas maltophilia	resistance-nodulation-cell division (RND) antibiotic efflux pump	aminoglycoside antibiotic;cephalosporin;cephamycin;penam	Multidrug	Antibiotic efflux	1418	3208	3126	1190	1246	1854	918	1212	1442	1568	1254	1632	20068
ARO:3003052	smeB	smeB is the inner membrane multidrug exporter of the efflux complex smeABC in Stenotrophomonas maltophilia	resistance-nodulation-cell division (RND) antibiotic efflux pump	aminoglycoside antibiotic;cephalosporin;cephamycin;penam	Multidrug	Antibiotic efflux	276	746	132	352	116	344	264	320	128	228	176	338	3420
ARO:3003053	smeC	smeC is an outer membrane multidrug efflux protein of the smeABC complex in Stenotrophomonas maltophilia	resistance-nodulation-cell division (RND) antibiotic efflux pump	aminoglycoside antibiotic;cephalosporin;cephamycin;penam	Multidrug	Antibiotic efflux	282	114	198	184	68	112	224	204	78	100	104	84	1752
ARO:3003055	smeD	smeD is the membrane fusion protein of the smeDEF multidrug efflux complex in Stenotrophomonas maltophilia	resistance-nodulation-cell division (RND) antibiotic efflux pump	fluoroquinolone antibiotic;macrolide antibiotic;phenicol antibiotic;tetracycline antibiotic	Multidrug	Antibiotic efflux	42	54	226	178	138	58	456	110	76	3900	3252	5916	14406
ARO:3003056	smeE	smeE is the RND protein of the efflux complex smeDEF in Stenotrophomonas maltophilia	resistance-nodulation-cell division (RND) antibiotic efflux pump	fluoroquinolone antibiotic;macrolide antibiotic;phenicol antibiotic;tetracycline antibiotic	Multidrug	Antibiotic efflux	40	96	128	162	58	102	304	194	92	654	880	748	3458
ARO:3003057	smeF	smeF is an outer membrane multidrug efflux protein of the smeDEF complex in Stenotrophomonas maltophilia	resistance-nodulation-cell division (RND) antibiotic efflux pump	fluoroquinolone antibiotic;macrolide antibiotic;phenicol antibiotic;tetracycline antibiotic	Multidrug	Antibiotic efflux	658	534	516	1182	570	420	1958	1602	630	872	1136	1128	11206
ARO:3003059	tmrB	tmrB is an ATP-binding tunicamycin resistance protein found in Bacillus subtilis	tunicamycin resistance protein	nucleoside antibiotic	Nucleoside	Reduced permeability to antibiotic	30	2	0	0	0	22	0	18	0	8	8	26	114
ARO:3003060	tsnR	tsnR is a 23S ribosomal RNA methyltransferase that methylates adenosine-1067 to	non-erm 23S ribosomal RNA	peptide antibiotic	Peptide	Antibiotic target	290	714	856	154	176	400	192	204	260	276	274	310	4106

		confer resistance to thiostrepton	methyltransferase (A1067)			alteration												
ARO:3003063	ykkC	ykkC is an SMR-type protein that is a subunit of the ykkCD efflux pump	small multidrug resistance (SMR) antibiotic efflux pump	aminoglycoside antibiotic;phenicol antibiotic;tetracycline antibiotic	Multidrug	Antibiotic efflux	392	176	214	594	262	238	884	706	272	262	308	204	4512
ARO:3003064	ykkD	ykkD is an SMR-type protein that is a subunit of the ykkCD efflux pump	small multidrug resistance (SMR) antibiotic efflux pump	aminoglycoside antibiotic;phenicol antibiotic;tetracycline antibiotic	Multidrug	Antibiotic efflux	318	586	22	104	66	330	62	80	78	144	116	182	2088
ARO:3003066	smeR	smeR is the responder component of a two component signal transduction system that includes smeS	resistance-nodulation-cell division (RND) antibiotic efflux pump	aminoglycoside antibiotic;cephalosporin;cephamycin;penam	Multidrug	Antibiotic efflux	1662	2202	1342	2340	1454	1270	3062	2622	1422	1120	1132	1304	20932
ARO:3003067	smeS	smeS is the protein kinase sensor component of a two component signal transduction system that includes smeR	resistance-nodulation-cell division (RND) antibiotic efflux pump	aminoglycoside antibiotic;cephalosporin;cephamycin;penam	Multidrug	Antibiotic efflux	6410	6574	6598	5726	8860	6040	7754	7298	8408	5816	6304	5914	81702
ARO:3003069	vanXYG	vanXYG is a vanXY variant found in the vanG gene cluster	glycopeptide resistance gene cluster;vanXY	glycopeptide antibiotic	Glycopeptide	Antibiotic target alteration	34	98	90	24	28	68	18	10	34	44	68	32	548
ARO:3003070	vanXD	vanXD is a vanX variant found in the vanD gene cluster	glycopeptide resistance gene cluster;vanX	glycopeptide antibiotic	Glycopeptide	Antibiotic target alteration	0	6	0	0	0	0	0	0	0	0	0	0	6
ARO:3003071	mphF	mphF is a macrolide phosphotransferase and resistance gene identified on the IncP plasmid pRSB111	macrolide phosphotransferase (MPH)	macrolide antibiotic	MLS	Antibiotic inactivation	6	6	68	74	1244	2	166	92	828	546	706	598	4336
ARO:3003093	cphA3	CEPH-A3 is an Ambler Class B MBL; subclass B2 originally isolated from Aeromonas veronii. This enzyme has specific activity against carbapenems and is active as a mono-zinc protein.	CphA beta-lactamase	carbapenem	Beta-lactam	Antibiotic inactivation	0	0	0	0	0	0	0	0	2	114	150	170	436

ARO:3003096	CfxA5	cfxA5 beta-lactamase is a class A beta-lactamase found in Bacteroides distasonis	CfxA beta-lactamase	cephamycin	Beta-lactam	Antibiotic inactivation	8	44	48	26	8	32	12	30	12	10	0	0	230
ARO:3003097	CfxA6	cfxA6 beta-lactamase is a class A beta-lactamase found in an uncultured bacterium	CfxA beta-lactamase	cephamycin	Beta-lactam	Antibiotic inactivation	136	346	220	84	16	228	68	90	76	4	4	4	1276
ARO:3003105	dfrA3	dfrA3 is an integron-encoded dihydrofolate reductase found in Escherichia coli	trimethoprim resistant dihydrofolate reductase dfr	diaminopyrimidine antibiotic	Diaminopyrimidine	Antibiotic target replacement	1156	1328	1190	1300	1286	796	1784	1400	1200	1858	2118	2402	17818
ARO:3003106	Erm(42)	Erm42 confers MLSb phenotype in Pasteurella multocida	Erm 23S ribosomal RNA methyltransferase	lincosamide antibiotic;macrolide antibiotic;streptogramin antibiotic	MLS	Antibiotic target alteration	74	390	666	262	1796	180	356	748	1632	4314	3798	6356	20572
ARO:3003107	mef(B)	mef(B) is a macrolide efflux gene located in the vicinity of sul3 in Escherichia coli. There is also a mefB found in Streptococcus agalactiae that confers resistance to macrolides.	major facilitator superfamily (MFS) antibiotic efflux pump	macrolide antibiotic	MLS	Antibiotic efflux	142	70	32	368	166	84	510	334	130	46	82	24	1988
ARO:3003109	msrE	MsrE is an ABC-F subfamily protein expressed to Klebsiella pneumoniae that confers resistance to erythromycin and streptogramin B antibiotics. It is associated with plasmid DNA. It is also 100% identical to ABC-F type ribosomal protection protein Msr(E) which is in multiple species.	ABC-F ATP-binding cassette ribosomal protection protein	lincosamide antibiotic;macrolide antibiotic;oxazolidinone antibiotic;phenicol antibiotic;pleuromutilin antibiotic;streptogramin antibiotic;tetracycline antibiotic	Multidrug	Antibiotic target protection	3762	7446	728	1242	3416	4302	1292	1256	3468	3222	3066	4338	37538
ARO:3003110	catB10	catB10 is an integron-encoded variant of the cat gene found in P. aeruginosa	chloramphenicol acetyltransferase (CAT)	phenicol antibiotic	Phenicol	Antibiotic inactivation	2	18	52	26	28	14	0	48	14	22	36	70	330
ARO:3003111	lsaB	LsaB is an ABC-F subfamily protein expressed in Staphylococcus sciuri. It confers resistance to clindamycin.	ABC-F ATP-binding cassette ribosomal	lincosamide antibiotic;macrolide antibiotic;oxazolidinone	Multidrug	Antibiotic target protection	2612	4288	3810	3478	1786	3096	3638	3768	2130	1458	1026	624	31714

			protection protein	antibiotic;phenicol antibiotic;pleuromutilin antibiotic;streptogramin antibiotic;tetracycline antibiotic															
ARO:3003112	lsaC	LsaC is an ABC-F subfamily protein expressed in Streptococcus agalactiae. It confers resistance to lincomycin, clindamycin, dalfopristin, and tiamulin	ABC-F ATP-binding cassette ribosomal protection protein	lincosamide antibiotic;macrolide antibiotic;oxazolidinone antibiotic;phenicol antibiotic;pleuromutilin antibiotic;streptogramin antibiotic;tetracycline antibiotic	Multidrug	Antibiotic target protection	3886	4298	2252	2404	1684	4098	2680	2610	2082	2844	2072	3226	34136
ARO:3003115	OXA-427	From the Lahey list of beta-lactamases. Not yet released.	OXA beta-lactamase	cephalosporin;penam	Beta-lactam	Antibiotic inactivation	0	0	0	16	0	0	6	58	0	0	0	0	80
ARO:3003171	ACT-36	From the Lahey list of beta-lactamases.	ACT beta-lactamase	carbapenem;cephalosporin;cephamycin;penam	Beta-lactam	Antibiotic inactivation	0	0	0	12	2	8	2	4	0	4	0	0	32
ARO:3003172	ACT-37	From the Lahey list of beta-lactamases.	ACT beta-lactamase	carbapenem;cephalosporin;cephamycin;penam	Beta-lactam	Antibiotic inactivation	170	6	16	2	4	126	0	0	36	82	64	124	630
ARO:3003183	NDM-14	NDM-14 is a beta-lactamase found in Acinetobacter lwoffii.	NDM beta-lactamase	carbapenem;cephalosporin;cephamycin;penam	Beta-lactam	Antibiotic inactivation	0	0	2	72	0	0	76	74	0	2	2	0	228
ARO:3003197	aadA25	streptomycin/spectinomycin resistance gene found in Pasteurella multocida isolated from bovine respiratory tract	ANT(3'')	aminoglycoside antibiotic	Aminoglycoside	Antibiotic inactivation	606	1234	1126	534	278	1118	294	572	450	144	70	40	6466
ARO:3003200	AAC(6')-Ian	AAC(6')-Ian is an amikacin acetyltransferase gene found on a transferable plasmid of the Serratia marcescens strain NUBL-11663. It catalyzes the transfer of an acetyl group from	AAC(6')	aminoglycoside antibiotic	Aminoglycoside	Antibiotic inactivation	70	2	34	32	0	38	30	28	4	6	0	0	244

		acetyl coenzyme A onto an amine at the 6'-position of various aminoglycosides.																
ARO:3003202	TLA-1	TLA-1 is a beta-lactamase found in plasmids of clinical isolates of Escherichia coli strain R170 in Latin America. It preferentially hydrolyzed cephaloridine, cefotaxime, cephalothin, benzylpenicillin, and ceftazidime. The enzyme was markedly inhibited by sulbactam, tazobactam, and clavulanic acid.	TLA beta-lactamase	cephalosporin;fluoroquinolone antibiotic;monobactam	Beta-lactam	Antibiotic inactivation	2	10	0	4	0	14	4	8	4	0	0	0	46
ARO:3003203	TLA-2	TLA-2 is a beta-lactamase is present on a plasmid isolated from an unidentified bacterial strain from a waste water treatment plant. The enzyme mostly hydrolyzes cephalosporins.	TLA beta-lactamase	cephalosporin;fluoroquinolone antibiotic;monobactam	Beta-lactam	Antibiotic inactivation	0	0	54	36	6	0	22	20	4	2	0	0	144
ARO:3003204	TLA-3	TLA-3 is a beta-lactamase found in a transferable plasmid of Serratia marcescens clinical isolate. It confers resistance to ceftazidime, cefotaxime and cefepime, but not to cefmetazole and meropenem	TLA beta-lactamase	cephalosporin;fluoroquinolone antibiotic;monobactam	Beta-lactam	Antibiotic inactivation	40	158	158	38	34	78	30	72	32	2	2	2	646
ARO:3003205	Erm(43)	Erm(43) is a macrolide, lincosamide, and streptogramin B resistance gene found in Staphylococcus lentus chromosome isolated from human, dog and chicken.	Erm 23S ribosomal RNA methyltransferase	lincosamide antibiotic;macrolide antibiotic;streptogramin antibiotic	MLS	Antibiotic target alteration	0	0	0	2	0	0	0	6	0	0	0	0	8
ARO:3003206	lsaE	lsaE is an ABC-F subfamily protein found in porcine MRSA isolates. It confers resistance to pleuromutilin, lincosamide, and streptogramin A	ABC-F ATP-binding cassette ribosomal protection protein	lincosamide antibiotic;macrolide antibiotic;oxazolidinone antibiotic;phenicol antibiotic;pleuromutilin antibiotic;streptogramin antibiotic;tetracycline antibiotic	Multidrug	Antibiotic target protection	16	22	44	10	6	34	34	30	10	144	96	74	520

ARO:3003207	FosK	FosK is a fosfomycin thiol transferase isolated from Acinetobacter soli. It is integrin-mediated. It confers a high level of resistance to fosfomycin.	fosfomycin thiol transferase	fosfomycin	Fosfomycin	Antibiotic inactivation	0	0	2	0	0	0	0	0	0	34	30	54	120
ARO:3003250	bcrC	The bcrC gene product (BcrC) is an undecaprenyl pyrophosphate phosphatase originally isolated from Bacillus subtilis. When overexpressed it can confer resistance to bacitracin.	undecaprenyl pyrophosphate related proteins	peptide antibiotic	Peptide	Antibiotic target alteration	0	0	0	0	0	0	6	0	0	0	0	0	6
ARO:3003318	Streptomyces rishiriensis parY mutant conferring resistance to aminocoumarin	point mutation on the Streptomyces rishiriensis parY resulting in aminocoumarin resistance	aminocoumarin resistant parY	aminocoumarin antibiotic	Aminocoumarin	Antibiotic target alteration	13148	16746	12802	10130	11774	14428	11428	12328	12870	16848	15228	19320	167050
ARO:3003324	Bacillus subtilis mprF	MprF is a integral membrane protein that modifies the negatively-charged phosphatidylglycerol on the membrane surface. This confers resistance to cationic peptides that disrupt the cell membrane, including defensins. Additionally, large-scale mutations causing loss of function of the gene result in increased susceptibility to daptomycin.	defensin resistant mprF	peptide antibiotic	Peptide	Antibiotic target alteration	0	0	30	0	0	0	0	0	2	2	6	0	40
ARO:3003440	mecB	A foreign PBP2 acquired by lateral gene transfer that able to perform peptidoglycan synthesis in the presence of beta-lactams.	methicillin resistant PBP2	carbapenem;cephalosporin;cephamycin;monobactam;penam	Beta-lactam	Antibiotic target replacement	1380	1076	1618	2234	4344	1246	3026	2894	4330	2462	2586	2640	29836
ARO:3003441	cfrA	CfrA is a chloramphenicol-florfenicol resistance gene and methyltransferase enzyme. Methylation of position 8 of A2503 in 23S rRNA confers resistance to chloramphenicol antibiotics first identified by	Cfr 23S ribosomal RNA methyltransferase	lincosamide antibiotic;phenicol antibiotic;pleuromutilin antibiotic;streptogramin antibiotic	Multidrug	Antibiotic target alteration	812	1192	980	1524	856	700	2026	1520	648	2050	1930	2752	16990

		Schwarz 2000 as cfr from Staphylococcus sciuri. Additional Oxazolidinone resistance mediated by the cfr gene in a human isolated was first reported from Colombia in linezolid- and methicillin-resistant Staphylococcus aureus (PMID: 10952608). Described by Arias et al. 2008. Chandela T. et al. (PMID: 28663118) grouped Cfr with ClbA in the Cfr Group.																
ARO:3003 548	mdtN	Multidrug resistance efflux pump. Could be involved in resistance to puromycin, acriflavine and tetraphenylarsonium chloride.	major facilitator superfamily (MFS) antibiotic efflux pump	acridine dye;nucleoside antibiotic	Multidrug	Antibiotic efflux	1192	642	534	1890	986	446	2638	1968	844	660	552	494	12846
ARO:3003 549	mdtO	Multidrug resistance efflux pump. Could be involved in resistance to puromycin, acriflavine and tetraphenylarsonium chloride	major facilitator superfamily (MFS) antibiotic efflux pump	acridine dye;nucleoside antibiotic	Multidrug	Antibiotic efflux	424	198	108	940	558	38	1520	1054	482	174	176	80	5752
ARO:3003 550	mdtP	Multidrug resistance efflux pump. Could be involved in resistance to puromycin, acriflavine and tetraphenylarsonium chloride	major facilitator superfamily (MFS) antibiotic efflux pump	acridine dye;nucleoside antibiotic	Multidrug	Antibiotic efflux	1032	380	242	1904	908	294	2684	1920	640	298	280	130	10712
ARO:3003 551	emeA	A multidrug efflux pump from Enterococcus faecalis. There exist efflux activity of several antimicrobial agents such as DAPI, Hoechst 33342 and acriflavine. Efflux of DAPI via EmeA was strongly inhibited by reserpine.	multidrug and toxic compound extrusion (MATE) transporter	acridine dye	Multidrug	Antibiotic efflux	38	20	108	10	4	62	42	14	10	34	66	110	518
ARO:3003 552	fusB	FusB encodes a 2-domain zinc-binding protein that binds the ribosomal translocase EF-G, causing it to dissociate from the ribosome. This action increases the ribosomal turnover rate and confers resistance to fusidic acid.	fusidic acid inactivation enzyme	fusidic acid	Fusidic acid	Antibiotic inactivation	2	8	0	0	0	0	0	0	0	0	0	0	10

ARO:3003553	CepS	CEPS is a typical class C cephalosporinase found in Aeromonas sobria, first isolated from strain 163a.	CepS beta-lactamase	cephalosporin	Beta-lactam	Antibiotic inactivation	0	24	14	0	10	6	0	0	10	6	4	8	82
ARO:3003557	SFB-1	This enzyme breaks the beta-lactam antibiotic ring open and deactivates the molecule's antibacterial properties.	SHW beta-lactamase	cephalosporin;penam	Beta-lactam	Antibiotic inactivation	0	0	0	0	0	0	0	0	0	54	22	54	130
ARO:3003559	cepA	A beta-lactamase found in Bacteroides fragilis producing either low or high levels of the endogenous cephalosporinase activity	cepA beta-lactamase	cephalosporin	Beta-lactam	Antibiotic inactivation	370	926	782	482	222	564	386	490	354	104	76	44	4800
ARO:3003563	RCP-1	RCP is a class A beta-lactamase found in Rhodopseudomonas capsulata.	RCP beta-lactamase	penam	Beta-lactam	Antibiotic inactivation	0	0	0	0	0	0	24	0	0	32	38	26	120
ARO:3003576	eptA	PmrC mediates the modification of Lipid A by the addition of 4-amino-4-deoxy-L-arabinose (L-Ara4N) and phosphoethanolamine, resulting in a less negative cell membrane and decreased binding of polymyxin B.	pmr phosphoethanolamine transferase	peptide antibiotic	Peptide	Antibiotic target alteration	1076	794	570	1254	706	408	1998	1590	740	368	344	218	10066
ARO:3003577	ugd	PmrE is required for the synthesis and transfer of 4-amino-4-deoxy-L-arabinose (Ara4N) to Lipid A, which allows gram-negative bacteria to resist the antimicrobial activity of cationic antimicrobial peptides and antibiotics such as polymyxin	pmr phosphoethanolamine transferase	peptide antibiotic	Peptide	Antibiotic target alteration	3268	3672	5382	2950	5352	3042	2882	3518	5254	3938	4158	4786	48202
ARO:3003578	PmrF	PmrF is required for the synthesis and transfer of 4-amino-4-deoxy-L-arabinose (Ara4N) to Lipid A, which allows gram-negative bacteria to resist the antimicrobial activity of cationic antimicrobial peptides and antibiotics such as polymyxin. pmrF corresponds to 1 locus in Pseudomonas aeruginosa PAO1 and 1 locus in Pseudomonas aeruginosa LESB58.	pmr phosphoethanolamine transferase	peptide antibiotic	Peptide	Antibiotic target alteration	5112	4910	4456	2464	1836	5728	2154	2568	2042	1736	1424	1742	36172

ARO:3003583	basS	Histidine protein kinase sensor Lipid A modification gene; part of a two-component system involved in polymyxin resistance that senses high extracellular Fe(2+)	pmr phosphoethanolamine transferase	peptide antibiotic	Peptide	Antibiotic efflux;antibiotic target alteration	3896	6870	2734	4372	7368	3764	5950	5774	7350	11468	10406	15916	85868
ARO:3003605	OXA-449	Assigned by Lahey's list of beta-lactamases, no accessions or other information available	OXA beta-lactamase	cephalosporin;penam	Beta-lactam	Antibiotic inactivation	0	0	30	36	4	0	20	50	4	4	0	0	148
ARO:3003617	OXA-461	Assigned by Lahey's list of beta-lactamases, no accessions or other information available	OXA beta-lactamase	cephalosporin;penam	Beta-lactam	Antibiotic inactivation	0	0	0	0	0	0	0	0	0	0	6	0	6
ARO:3003620	OXA-464	Assigned by Lahey's list of beta-lactamases, no accessions or other information available	OXA beta-lactamase	cephalosporin;penam	Beta-lactam	Antibiotic inactivation	0	0	6	0	8	0	0	0	4	98	38	68	222
ARO:3003643	OXA-486	Assigned by Lahey's list of beta-lactamases, no accessions or other information available	OXA beta-lactamase	cephalosporin;penam	Beta-lactam	Antibiotic inactivation	0	0	0	0	0	0	0	0	0	4	10	0	14
ARO:3003645	OXA-488	Assigned by Lahey's list of beta-lactamases, no accessions or other information available	OXA beta-lactamase	cephalosporin;penam	Beta-lactam	Antibiotic inactivation	0	0	64	2	22	0	0	4	12	26	48	44	222
ARO:3003665	NmcR	NmcR is a homolog of the LysR regulator found in Enterobacter cloacae that contribute to the regulation of NmcA beta-lactamase	NmcA beta-lactamase	carbapenem;cephalosporin;cephamycin;penam	Beta-lactam	Antibiotic inactivation	2558	1510	1876	3104	5474	1936	4244	4152	4936	4362	4458	5012	43622
ARO:3003670	PEDO-1	PEDO-1 is a subclass B3 metallo-beta lactamase isolated from Pedobacter roseus exhibiting resistance to carbapenems.	subclass B3 PEDO beta-lactamase	carbapenem	Beta-lactam	Antibiotic inactivation	50	232	56	10	2	140	62	34	20	12	12	6	636
ARO:3003677	AAC(6')-Iaj	AAC(6')-Iaj is a functional acetyltransferase that modifies the amino groups at the 6' positions of aminoglycosides and contributes to aminoglycoside resistance of P. aeruginosa	AAC(6')	aminoglycoside antibiotic	Aminoglycoside	Antibiotic inactivation	0	0	0	0	0	0	0	0	0	4	12	14	30
ARO:3003679	TriA	TriA is a membrane protein that is fused to TriB and both are required for the triclosan efflux	resistance-nodulation-cell division (RND)	triclosan	Triclosan	Antibiotic efflux	1196	2230	458	624	1478	1490	646	850	1112	2266	1766	3788	17904

		pump function of TriABC-OpmH in P. aeruginosa.	antibiotic efflux pump															
ARO:3003680	TriB	TriB is a membrane protein that is fused to TriA and both are required for the triclosan efflux pump function of TriABC-OpmH in P. aeruginosa.	resistance-nodulation-cell division (RND) antibiotic efflux pump	triclosan	Triclosan	Antibiotic efflux	276	28	218	90	34	196	72	124	78	188	372	308	1984
ARO:3003681	TriC	TriC is a resistance nodulation cell division (RND) transporter that is a part of TriABC-OpmH, a triclosan-specific efflux protein.	resistance-nodulation-cell division (RND) antibiotic efflux pump	triclosan	Triclosan	Antibiotic efflux	370	384	1158	810	5284	332	1192	1090	5068	2624	2336	3072	23720
ARO:3003682	OpmH	OpmH is an outer membrane efflux protein required for triclosan-specific efflux pump function.	resistance-nodulation-cell division (RND) antibiotic efflux pump	triclosan	Triclosan	Antibiotic efflux	1070	2078	1080	912	5928	1202	1304	1690	5398	6368	5868	8934	41832
ARO:3003692	MexJ	mexJ is the membrane fusion protein of the MexJK multidrug efflux protein.	resistance-nodulation-cell division (RND) antibiotic efflux pump	macrolide antibiotic;tetracycline antibiotic;triclosan	Multidrug	Antibiotic efflux	1102	2594	2488	1842	12350	1522	2186	3150	11656	5872	5480	6376	56618
ARO:3003693	MexK	mexK is the inner membrane resistance-nodulation-cell division (RND) transporter in the MexJK multidrug efflux protein.	resistance-nodulation-cell division (RND) antibiotic efflux pump	macrolide antibiotic;tetracycline antibiotic;triclosan	Multidrug	Antibiotic efflux	1460	2616	1594	1402	4430	1616	1840	2032	3984	6640	6206	8738	42558
ARO:3003698	mexP	MexP is the membrane fusion protein of the MexPQ-OpmE multidrug efflux complex	resistance-nodulation-cell division (RND) antibiotic efflux pump	acridine dye;carbapenem;diaminopyrimidine antibiotic;macrolide antibiotic;phenicol antibiotic;tetracycline antibiotic	Multidrug	Antibiotic efflux	36	158	198	92	84	76	70	72	84	90	110	118	1188
ARO:3003699	mexQ	MexQ is the inner membrane transporter of the multidrug efflux pump MexPQ-OpmE.	resistance-nodulation-cell division (RND)	acridine dye;carbapenem;diaminopyrimidine antibiotic;macrol	Multidrug	Antibiotic efflux	44	122	100	96	16	70	112	104	22	1270	828	1272	4056

			antibiotic efflux pump	ide antibiotic;phenicol antibiotic;tetracycline antibiotic															
ARO:3003700	opmE	opmE is an outer membrane factor protein that is part of the multidrug efflux pump MexPQ-OpmE.	resistance-nodulation-cell division (RND) antibiotic efflux pump	acridine dye;carbapenem;diaminopyrimidine antibiotic;macrolide antibiotic;phenicol antibiotic;tetracycline antibiotic	Multidrug	Antibiotic efflux	98	38	110	44	24	50	36	78	22	366	388	442	1696
ARO:3003704	mexM	mexM is the membrane fusion protein of the MexMN-OprM multidrug efflux complex.	resistance-nodulation-cell division (RND) antibiotic efflux pump	phenicol antibiotic	Multidrug	Antibiotic efflux	184	120	112	70	288	144	94	108	408	900	642	1002	4072
ARO:3003705	mexN	MexN is the inner membrane transporter of the MexMN-OprM multidrug efflux complex.	resistance-nodulation-cell division (RND) antibiotic efflux pump	phenicol antibiotic	Multidrug	Antibiotic efflux	352	480	468	328	500	410	384	392	428	3982	3440	5376	16540
ARO:3003710	MexL	MexL is a specific repressor of mexJK transcription and autoregulates its own expression.	resistance-nodulation-cell division (RND) antibiotic efflux pump	macrolide antibiotic;tetracycline antibiotic;triclosan	Multidrug	Antibiotic efflux	712	1566	440	428	874	888	450	480	730	1984	2182	2540	13274
ARO:3003714	PEDO-2	PEDO-2 is a subclass B3 metallo-beta lactamase isolated from Pedobacter borealis exhibiting carbapenem resistance.	subclass B3 PEDO beta-lactamase	carbapenem	Beta-lactam	Antibiotic inactivation	68	14	14	76	30	36	54	106	26	16	20	4	464
ARO:3003715	PEDO-3	PEDO-3 is a class B1 metallo-beta lactamase isolated from Pedobacter kyungheensis exhibiting resistance to carbapenems.	subclass B1 PEDO beta-lactamase	carbapenem	Beta-lactam	Antibiotic inactivation	0	0	0	0	0	0	0	0	0	86	120	122	328
ARO:3003717	ESP-1	ESP-1 is a subclass B3 metallo-beta lactamase isolated from Epilithonimonas tenax conferring resistance to carbapenems.	ESP beta-lactamase	carbapenem	Beta-lactam	Antibiotic inactivation	0	8	4	2	4	4	0	0	18	6	4	6	56

ARO:3003718	MSI-1	MSI-1 is a subclass B3 metallo-beta lactamase isolated from Massilia oculi conferring resistance to carbapenems.	MSI beta-lactamase	carbapenem	Beta-lactam	Antibiotic inactivation	86	96	216	188	68	174	128	118	42	40	60	32	1248
ARO:3003720	SPG-1	SPG-1 is a subclass B3 metallo-beta lactamase isolated from Sphingomonas sp.	SPG beta-lacatamase	carbapenem	Beta-lactam	Antibiotic inactivation	10	48	8	12	8	20	14	18	2	32	16	16	204
ARO:3003723	vanI	VanI is a D-Ala-D-Lac ligase that reduces vancomycin binding affinity, helping to confer vancomycin resistance as part of the VanI resistance gene cluster in Desulfitobacterium spp. and Desulfosporosinus spp.	glycopeptide resistance gene cluster;van ligase	glycopeptide antibiotic	Glycopeptide	Antibiotic target alteration	116	124	138	136	140	66	190	174	114	164	132	152	1646
ARO:3003724	vanWI	VanWI is a vanW variant found in the vanI gene cluster	glycopeptide resistance gene cluster;van W	glycopeptide antibiotic	Glycopeptide	Antibiotic target alteration	586	494	1016	668	322	782	676	664	450	708	586	524	7476
ARO:3003725	vanXI	VanXI is a VanX variant found in the VanI glycopeptide resistance gene cluster. It is a D-Ala-D-Ala dipeptidase.	glycopeptide resistance gene cluster;vanX	glycopeptide antibiotic	Glycopeptide	Antibiotic target alteration	280	328	614	4	154	180	12	18	172	12	26	16	1816
ARO:3003727	vanKI	VanKI is a peptidoglycan bridge formation protein also known as FemAB that is part of the VanI glycopeptide resistance gene cluster.	glycopeptide resistance gene cluster;vanK	glycopeptide antibiotic	Glycopeptide	Antibiotic target alteration	22	344	68	96	36	78	66	80	42	14	8	6	860
ARO:3003728	vanRI	VanRI is the regulatory transcriptional activator in the VanSR regulator within the VanI glycopeptide resistance gene cluster.	glycopeptide resistance gene cluster;vanR	glycopeptide antibiotic	Glycopeptide	Antibiotic target alteration	3782	5120	3182	2368	1752	3284	2696	2500	1702	2412	2176	2422	33396
ARO:3003730	Bifidobacterium ileS conferring resistance to mupirocin	Bifidobacteria have an intrinsically resistant form of ileS (isoleucyl-tRNA synthetase) that confers resistance to mupirocin.	antibiotic-resistant isoleucyl-tRNA synthetase (ileS)	mupirocin	Mupirocin	Antibiotic target alteration	1528	800	906	660	492	1150	698	702	656	810	1310	770	10482
ARO:3003733	fusC	FusC is a fusidic acid resistance gene enabling ribosomal translocase EF-G dissociation from the ribosome that has	fusidic acid inactivation enzyme	fusidic acid	Fusidic acid	Antibiotic inactivation	48	2	0	0	0	14	0	0	0	0	2	0	66

		been detected in Staphylococcus aureus and Staphylococcus intermedius. Its mechanism is believed to be similar to fusB due to its high level of sequence homology.																
ARO:3003741	mphE	mphE is a macrolide phosphotransferase and resistance gene identified on a plasmid, pRSB105	macrolide phosphotransferase (MPH)	macrolide antibiotic	MLS	Antibiotic inactivation	3500	8130	590	1212	3140	4098	1114	1220	3046	6082	4860	8146	45138
ARO:3003742	mphG	The mphG gene encodes a macrolide 2'-phosphotransferase found in Photobacterium damselae sharing sequence similarity to mphA in E. coli.	macrolide phosphotransferase (MPH)	macrolide antibiotic	MLS	Antibiotic inactivation	2	12	24	10	40	0	46	8	26	474	334	492	1468
ARO:3003744	vatF	vatF is a streptogramin A acetyl transferase gene isolated from the chromosome of Yersinia enterocolitica.	streptogramin vat acetyltransferase	streptogramin antibiotic	MLS	Antibiotic inactivation	736	1628	576	442	1260	904	342	834	1148	920	862	1072	10724
ARO:3003745	mefC	mefC is a macrolide efflux gene isolated from a plasmid in Photobacterium damselae.	major facilitator superfamily (MFS) antibiotic efflux pump	macrolide antibiotic	MLS	Antibiotic efflux	280	630	232	80	84	422	114	164	80	826	564	738	4214
ARO:3003746	optrA	OptrA is a member of the ABC-F protein subfamily that confers resistance to oxazolidinones. The gene encoding the protein was originally isolated from a plasmid in Enterococcus faecalis and Enterococcus faecium.	ABC-F ATP-binding cassette ribosomal protection protein	lincosamide antibiotic;macrolide antibiotic;oxazolidinone antibiotic;phenicol antibiotic;pleuromutilin antibiotic;streptogramin antibiotic;tetracycline antibiotic	Multidrug	Antibiotic target protection	9408	10976	11092	7732	9312	10010	8936	9436	10108	12988	12400	15096	127494
ARO:3003748	oleC	oleC is an ABC transporter isolated from Streptomyces antibioticus and is involved in oleandomycin secretion.	ATP-binding cassette (ABC) antibiotic efflux pump	macrolide antibiotic	MLS	Antibiotic efflux	8042	7810	11676	7094	20156	8550	9290	10138	20622	13974	14650	14546	146548
ARO:3003749	salA	salA is an ABC-F subfamily protein gene isolated from the chromosome of Staphylococcus	ABC-F ATP-binding cassette	lincosamide antibiotic;macrolide	Multidrug	Antibiotic target	790	1012	548	450	230	914	280	414	326	828	826	1100	7718

		sciuri conferring resistance to lincosamides and streptogramins.	ribosomal protection protein	antibiotic;oxazolidinone antibiotic;phenicol antibiotic;pleuromutilin antibiotic;streptogramin antibiotic;tetracycline antibiotic		protection													
ARO:3003762	lnuE	lnuE encodes a lincosamide resistance gene isolated from Streptococcus suis that was truncated by an ISEnfa5-cfr-ISEnfa5 segment insertion. It shares the closest sequence similarity to lnuA.	lincosamide nucleotidyltransferase (LNU)	lincosamide antibiotic	MLS	Antibiotic inactivation	268	570	460	306	158	504	230	270	180	120	64	52	3182
ARO:3003767	mphM	mphM is a chromosomally-encoded macrolide phosphotransferases that inactivate 14-, 15- and 16-membered macrolides.	macrolide phosphotransferase (MPH)	macrolide antibiotic	MLS	Antibiotic inactivation	14	38	32	50	6	78	36	64	66	74	70	100	628
ARO:3003770	Listeria monocytogenes mprF	MprF is a integral membrane protein that modifies the negatively-charged phosphatidylglycerol on the membrane surface. This confers resistance to cationic peptides that disrupt the cell membrane, including defensins.	defensin resistant mprF	peptide antibiotic	Peptide	Antibiotic target alteration	72	66	4	0	10	162	12	4	120	28	26	8	512
ARO:3003772	Brucella suis mprF	MprF is a integral membrane protein that modifies the negatively-charged phosphatidylglycerol on the membrane surface. This confers resistance to cationic peptides that disrupt the cell membrane, including defensins.	defensin resistant mprF	peptide antibiotic	Peptide	Antibiotic target alteration	218	14	40	0	12	222	58	8	34	2	8	4	620
ARO:3003773	Clostridium perfringens mprF	MprF is a integral membrane protein that modifies the negatively-charged phosphatidylglycerol on the membrane surface. This confers resistance to cationic peptides that disrupt the cell membrane, including defensins.	defensin resistant mprF	peptide antibiotic	Peptide	Antibiotic target alteration	0	18	4	0	6	26	0	0	4	2	16	2	78

ARO:3003774	Streptococcus agalactiae mprF	MprF is a integral membrane protein that modifies the negatively-charged phosphatidylglycerol on the membrane surface. This confers resistance to cationic peptides that disrupt the cell membrane, including defensins.	defensin resistant mprF	peptide antibiotic	Peptide	Antibiotic target alteration	0	0	6	2	2	0	4	12	4	16	50	12	108
ARO:3003785	Chlamydia trachomatis intrinsic murA conferring resistance to fosfomycin	Chlamydia murA confers intrinsic resistance to fosfomycin. The presence of an aspartic acid residue in place of the critical cysteine at position 119 that enables fosfomycin binding is believed to be responsible for this intrinsic resistance.	antibiotic-resistant murA transferase	fosfomycin	Fosfomycin	Antibiotic target alteration	5530	7940	5304	3764	2732	6718	4240	4020	3186	5282	5090	6628	60434
ARO:3003801	bcr-1	Transmembrane protein which expels bicyclomycin from the cell, leading to bicyclomycin resistance. Identified in Pseudomonas aeruginosa strains responsible for outbreaks in Brazil, often appearing with blaSPM-1, another bicyclomycin resistance gene	major facilitator superfamily (MFS) antibiotic efflux pump	bicyclomycin	Bicyclomycin	Antibiotic efflux	2420	2100	1108	2168	1638	2028	3064	2276	1732	2522	2654	3516	27226
ARO:3003811	adeC	AdeC is the outer membrane factor of the AdeABC multidrug efflux complex. It can be replaced by other outer membrane channels, and is not essential for antibiotic resistance.	resistance-nodulation-cell division (RND) antibiotic efflux pump	glycylcycline;tetracycline antibiotic	Multidrug	Antibiotic efflux	0	0	10	0	2	14	20	0	0	452	282	286	1066
ARO:3003835	cdeA	Clostridioides difficile and Escherichia coli multidrug efflux transporter with antiporter function. Confers resistance to fluoroquinolones in E. coli and acriflavin in Clostridioides difficile.	multidrug and toxic compound extrusion (MATE) transporter	acridine dye;fluoroquinolone antibiotic	Multidrug	Antibiotic efflux	1420	2338	2550	884	998	1722	948	998	1238	1142	776	866	15880
ARO:3003836	qacH	qacH is a subunit of the qac multidrug efflux pump in Vibrio cholerae	small multidrug resistance	fluoroquinolone antibiotic	Multidrug	Antibiotic efflux	112	390	382	614	1152	68	1122	916	1418	4602	4464	6576	21816

			(SMR) antibiotic efflux pump																
ARO:3003 838	gadW	GadW is an AraC-family regulator that promotes mdtEF expression to confer multidrug resistance. GadW inhibits GadX-dependent activation. GadW clearly represses gadX and, in situations where GadX is missing, activates gadA and gadBC.	resistance-nodulation-cell division (RND) antibiotic efflux pump	fluoroquinolone antibiotic;macrolide antibiotic;penam	Multidrug	Antibiotic efflux	860	226	184	1414	650	150	1950	1758	552	232	270	66	8312
ARO:3003 841	kdpE	kdpE is a transcriptional activator that is part of the two-component system KdpD/KdpE that is studied for its regulatory role in potassium transport and has been identified as an adaptive regulator involved in the virulence and intracellular survival of pathogenic bacteria. kdpE regulates a range of virulence loci through direct promoter binding.	kdpDE	aminoglycoside antibiotic	Aminoglycoside	Antibiotic efflux	3560	3854	3178	2758	1738	3256	3464	3158	2052	2548	2278	2606	34450
ARO:3003 842	MUS-2	MUS-2 is a chromosome-encoded beta-lactamase from Myroides odoratimimus.	MUS beta-lactamase	carbapenem	Beta-lactam	Antibiotic inactivation	0	0	0	4	0	0	68	4	0	0	0	0	76
ARO:3003 852	ADC-6	ADC-6 is a beta-lactamase found in Acinetobacter baumannii.	ADC beta-lactamase without carbapenemase activity	cephalosporin	Beta-lactam	Antibiotic inactivation	0	0	0	2	0	0	20	2	0	2	0	0	26
ARO:3003 862	ADC-18	ADC-18 is a beta-lactamase found in Acinetobacter pittii.	ADC beta-lactamase without carbapenemase activity	cephalosporin	Beta-lactam	Antibiotic inactivation	0	0	6	8	10	0	12	12	6	82	88	40	264
ARO:3003 863	ADC-19	ADC-19 is a beta-lactamase found in Acinetobacter pittii.	ADC beta-lactamase without carbapenemase activity	cephalosporin	Beta-lactam	Antibiotic inactivation	0	2	2	2	0	8	2	0	0	2	0	0	18
ARO:3003 867	ADC-23	ADC-23 is a beta-lactamase found in Acinetobacter pittii.	ADC beta-lactamase without	cephalosporin	Beta-lactam	Antibiotic inactivation	0	0	0	0	0	0	0	0	0	4	18	4	26

			carbapenemase activity																
ARO:3003905	ANT(4')-Ib	Kanamycin nucleotidyltransferase sequence from Staphylococcus aureus plasmid. Confers resistance to kanamycin, neomycin and other aminoglycosides	ANT(4')	aminoglycoside antibiotic	Aminoglycoside	Antibiotic inactivation	40	122	52	2	80	132	12	10	58	344	496	448	1796
ARO:3003907	cipA	Cfr-like methyltransferase enzyme conferring resistance to multiple clinically relevant antibiotic classes.	Cfr 23S ribosomal RNA methyltransferase	lincosamide antibiotic;phenicol antibiotic;pleuromutilin antibiotic;streptogramin antibiotic	Multidrug	Antibiotic target alteration	236	312	192	332	224	336	406	292	506	556	1100	594	5086
ARO:3003908	Erm(47)	Chromosome-encoded gene conferring MLSB resistance. Identified from Helcococcus kunzii.	Erm 23S ribosomal RNA methyltransferase	lincosamide antibiotic;macrolide antibiotic;streptogramin antibiotic	MLS	Antibiotic target alteration	570	552	486	64	94	634	92	134	142	138	114	60	3080
ARO:3003918	apmA	Plasmid-borne apramycin-resistant aminocyclitol acetyltransferase gene identified from bovine MRSA.	amp acetyltransferase	aminoglycoside antibiotic	Aminoglycoside	Antibiotic inactivation	306	378	210	168	194	266	138	246	176	296	166	138	2682
ARO:3003920	pgpB	A gene that produces the protein lipid A 4'-phosphatase	lipid A phosphatase	peptide antibiotic	Peptide	Antibiotic target alteration	192	232	204	86	70	244	50	96	64	462	352	666	2718
ARO:3003922	oqxA	RND efflux pump conferring resistance to fluoroquinolone	resistance-nodulation-cell division (RND) antibiotic efflux pump	diaminopyrimidine antibiotic;fluoroquinolone antibiotic;glycylcycline;nitrofuran antibiotic;tetracycline antibiotic	Multidrug	Antibiotic efflux	1246	920	822	1460	918	842	1874	1650	792	2906	2136	3952	19518
ARO:3003923	oqxB	RND efflux pump conferring resistance to fluoroquinolone	resistance-nodulation-cell division (RND) antibiotic efflux pump	diaminopyrimidine antibiotic;fluoroquinolone antibiotic;glycylcycline;nitrofuran antibiotic;tetracycline antibiotic	Multidrug	Antibiotic efflux	1018	796	680	1988	1070	296	3124	3124	932	1594	1612	2028	18262
ARO:3003942	abcA	AbcA is a multidrug resistant ABC transporter that confers resistance to methicillin,	ATP-binding cassette (ABC)	cephalosporin;penam;peptide antibiotic	Multidrug	Antibiotic efflux	234	496	376	138	114	358	110	140	178	1416	1354	1602	6516

		daptomycin, cefotaxime, and moenomycin.	antibiotic efflux pump																
ARO:3003948	efrA	efrA is a part of the EfrAB efflux pump, and both efrA and efrB are necessary to confer drug resistance.	ATP-binding cassette (ABC) antibiotic efflux pump	fluoroquinolone antibiotic;macrolide antibiotic;rifamycin antibiotic	Multidrug	Antibiotic efflux	12022	9976	9344	6834	8696	12718	8478	8498	9484	6038	5760	4940	102788
ARO:3003949	efrB	efrB is a part of the EfrAB efflux pump, and both efrA and efrB are necessary to confer multidrug resistance.	ATP-binding cassette (ABC) antibiotic efflux pump	fluoroquinolone antibiotic;macrolide antibiotic;rifamycin antibiotic	Multidrug	Antibiotic efflux	2582	3130	3714	2460	3400	2742	2944	2806	3788	7214	5942	8682	49404
ARO:3003950	msbA	MsbA is a multidrug resistance transporter homolog from E. coli and belongs to a superfamily of transporters that contain an adenosine triphosphate (ATP) binding cassette (ABC) which is also called a nucleotide-binding domain (NBD). MsbA is a member of the MDR-ABC transporter group by sequence homology. MsbA transports lipid A, a major component of the bacterial outer cell membrane, and is the only bacterial ABC transporter that is essential for cell viability.	ATP-binding cassette (ABC) antibiotic efflux pump	nitroimidazole antibiotic	Multidrug	Antibiotic efflux	23286	19502	14692	17422	21564	21232	23002	21012	21140	22716	22970	26294	254832
ARO:3003952	YojI	YojI mediates resistance to the peptide antibiotic microcin J25 when it is expressed from a multicopy vector. YojI is capable of pumping out microcin molecules. The outer membrane protein TolC in addition to YojI is required for export of microcin J25 out of the cell. Microcin J25 is thus the first known substrate for YojI.	ATP-binding cassette (ABC) antibiotic efflux pump	peptide antibiotic	Peptide	Antibiotic efflux	3418	2798	2408	3494	1920	2918	5014	4292	2424	1416	1524	1072	32698
ARO:3003953	hmrM	hmrM is a multidrug efflux pump belonging to the MATE family and functions as a Na+/drug antiporter.	multidrug and toxic compound extrusion (MATE) transporter	acridine dye;fluoroquinolone antibiotic	Multidrug	Antibiotic efflux	432	424	190	968	436	160	1298	1208	402	1030	938	1228	8714

ARO:3003954	efmA	efmA is an MFS transporter permease in E. faecium.	major facilitator superfamily (MFS) antibiotic efflux pump	fluoroquinolone antibiotic;macrolide antibiotic	Multidrug	Antibiotic efflux	440	348	138	24	174	464	62	34	162	390	350	524	3110
ARO:3003955	efpA	efpA is an MFS transporter found in Mycobacterium tuberculosis.	major facilitator superfamily (MFS) antibiotic efflux pump	isoniazid;rifamycin antibiotic	Multidrug	Antibiotic efflux	706	1746	108	228	200	840	182	228	144	686	684	1010	6762
ARO:3003961	farA	farA is the membrane fusion protein that is part of the farAB efflux pump.	major facilitator superfamily (MFS) antibiotic efflux pump	antibacterial free fatty acids	Multidrug	Antibiotic efflux	1294	1196	1644	2458	1376	832	3456	2588	1234	1406	1366	1652	20502
ARO:3003962	farB	farB is the cytoplasmic transporter protein that is part of the farAB efflux pump. farB corresponds to 3 loci in Pseudomonas aeruginosa PAO1 and 3 loci in Pseudomonas aeruginosa LESB58.	major facilitator superfamily (MFS) antibiotic efflux pump	antibacterial free fatty acids	Multidrug	Antibiotic efflux	28	16	160	42	82	62	126	28	76	242	262	220	1344
ARO:3003964	hp1181	hp1181 is a translocase that is part of the MFS efflux pump family. It is found in H. pylori and has role in the active efflux of antibiotics.	major facilitator superfamily (MFS) antibiotic efflux pump	fluoroquinolone antibiotic;nitroimidazole antibiotic;tetracycline antibiotic	Multidrug	Antibiotic efflux	590	1802	870	694	4656	842	878	1072	4086	1944	1846	2164	21444
ARO:3003967	lfrA	lfrA is involved in the active efflux of quinolones and is found in Mycobacteroides abscessus.	major facilitator superfamily (MFS) antibiotic efflux pump	fluoroquinolone antibiotic	Fluoroquinolone	Antibiotic efflux	92	30	60	94	84	68	288	98	162	852	1504	870	4202
ARO:3003971	Erm(44)v	Variant of Erm(44)v isolated from Staphylococcus saprophyticus, confers resistance to lincosamide and macrolide antibiotics but not streptogramins.	Erm 23S ribosomal RNA methyltransferase	lincosamide antibiotic;macrolide antibiotic;streptogramin antibiotic	MLS	Antibiotic target alteration	128	12	14	14	2	36	90	22	0	10	0	4	332
ARO:3003980	tetA(58)	TetA(58) is a Tetracycline efflux pump described in Paenibacillus sp. LC231, a strain of Paenibacillus isolated from	major facilitator superfamily (MFS)	tetracycline antibiotic	Tetracycline	Antibiotic efflux	18244	19722	19138	13676	16042	17726	18214	16114	17426	20182	21578	21610	219672

		Lechuguilla Cave, NM, USA. Described by Pawlowski et al. 2016.	antibiotic efflux pump																
ARO:3003981	tetB(58)	Tetracycline resistant TetB(58) efflux pump found in Paenibacillus sp. LC231, a strain of Paenibacillus isolated from Lechuguilla Cave, NM, USA. Described by Pawlowski et al. 2016.	major facilitator superfamily (MFS) antibiotic efflux pump	tetracycline antibiotic	Tetracycline	Antibiotic efflux	64	12	10	14	14	48	84	12	68	114	392	132	964
ARO:3003982	LlmA 23S ribosomal RNA methyltransferase	Lincosamide (Clindamycin) resistant (putative) ribosomal methyltransferase related to the RlmK 23S rRNA methyltransferase COG family. Detected in Paenibacillus sp. LC231, an isolated strain of Paenibacillus found in Lechuguilla Cave, NM, USA. Described by Pawlowski et al. 2016.	Llm 23S ribosomal RNA methyltransferase	lincosamide antibiotic	MLS	Antibiotic target alteration	1812	3452	2710	1580	880	2340	1420	1642	1120	856	734	440	18986
ARO:3003983	CatU	Chloramphenicol acetyltransferase conferring resistance to phenicol antibiotics detected in Paenibacillus sp. LC231, an isolated strain of Paenibacillus from Lechuguilla Cave, NM, USA. Described by Pawlowski et al. 2016.	chloramphenicol acetyltransferase (CAT)	phenicol antibiotic	Phenicol	Antibiotic inactivation	4	10	12	2	8	8	16	14	14	10	10	4	112
ARO:3003984	BahA	Bacitracin amidohydrolase found in Paenibacillus sp. LC231, an isolated strain of Paenibacillus from Lechuguilla Cave, NM, USA. Confers resistance by bacitracin inactivation through amidohydrolysis. Described by Pawlowski et al. 2016.	Bah amidohydrolase	peptide antibiotic	Peptide	Antibiotic inactivation	146	14	0	0	0	36	0	0	0	40	74	64	374
ARO:3003986	TaeA	Pleuromutilin (Tiamulin) ABC efflux pump found in Paenibacillus sp. LC231, a strain of Paenibacillus isolated from Lechuguilla Cave, NM, USA. Confers resistance to pleuromutilin antibiotics.	ATP-binding cassette (ABC) antibiotic efflux pump	pleuromutilin antibiotic	Pleuromutilin	Antibiotic efflux	13330	12628	10672	9040	12758	10710	10828	10742	13048	17404	16768	21480	159408

		Described by Pawlowski et al. 2016.																	
ARO:3003987	VatI	Streptogramin A acetyltransferase found in Paenibacillus sp. LC231, isolated from Lechuguilla Cave, NM, USA. Confers resistance to streptogramin A antibiotics. Described by Pawlowski et al. 2016.	streptogramin vat acetyltransferase	streptogramin antibiotic	MLS	Antibiotic inactivation	84	504	374	164	46	364	88	136	100	78	66	30	2034
ARO:3003988	AAC(2')-IIb	AAC(2')-IIb is an intrinsic Kasugamycin 2' acetyltransferase protein found in Paenibacillus sp. LC231, a strain of Paenibacillus isolated from Lechuguilla Cave, NM, USA. Confers resistance to aminoglycoside antibiotics, including kasugamycin. Described by Pawlowski et al. 2016.	AAC(2')	aminoglycoside antibiotic	Aminoglycoside	Antibiotic inactivation	0	18	18	6	10	0	10	4	6	6	0	12	90
ARO:3003989	AAC(6')-34	AAC(6')-34 is an aminoglycoside 6'-phosphotransferase found in Paenibacillus sp. LC231, a strain of Paenibacillus from Lechuguilla Cave, NM, USA. Confers resistance to aminoglycoside antibiotics. Described by Pawlowski et al. 2016.	AAC(6')	aminoglycoside antibiotic	Aminoglycoside	Antibiotic inactivation	108	12	2	0	0	8	0	0	0	0	0	0	130
ARO:3003990	vgbC	VgbC is a streptogramin B-type lyase found in Paenibacillus sp. LC231, a strain of Paenibacillus isolated from Lechuguilla Cave, NM, USA. Confers resistance to streptogramin B-type antibiotics by linearization of the lactone ring on an ester bond, resulting in antibiotic inactivation. Described by Pawlowski et al. 2016.	streptogramin vgb lyase	streptogramin antibiotic	MLS	Antibiotic inactivation	0	0	10	0	6	0	0	0	0	16	18	12	62
ARO:3003992	rphB	rphB is a rifampin phosphotransferase protein found in Paenibacillus sp. LC231, a strain of Paenibacillus isolated from Lechuguilla Cave, NM, USA. Confers resistance to	rifampin phosphotransferase	rifamycin antibiotic	Rifamycin	Antibiotic inactivation	1558	564	1376	1154	1280	970	1914	1672	1004	3118	2788	4122	21520

		rifamycin antibiotics, specifically rifampin, through rifampin inactivation. Described by Pawlowski et al. 2016.																
ARO:3003994	cpaA	cpaA is a capreomycin acetyltransferase protein found in Paenibacillus sp. LC231, an isolated strain of Paenibacillus in Lechuguilla Cave, NM, USA. Confers resistance to capreomycin, an aminoglycoside antibiotic. Described by Pawlowski et al. 2016.	cpa acetyltransferase	aminoglycoside antibiotic	Aminoglycoside	Antibiotic inactivation	30	4	0	2	0	16	0	0	0	0	0	0	52
ARO:3004032	tetA(46)	tetA(46) is a subunit of tetAB(46), a heterodimeric ABC transporter, that is required for conferring tetracycline resistance in Streptococcus australis isolated from the oral cavity.	ATP-binding cassette (ABC) antibiotic efflux pump	tetracycline antibiotic	Tetracycline	Antibiotic efflux	3206	2642	2496	1104	1026	4728	1284	1414	1190	3920	3624	5246	31880
ARO:3004033	tetB(46)	tetB(46) is a subunit of tetAB(46), a heterodimeric ABC transporter, that is required for conferring tetracycline resistance in Streptococcus australis isolated from the oral cavity.	ATP-binding cassette (ABC) antibiotic efflux pump	tetracycline antibiotic	Tetracycline	Antibiotic efflux	4040	3592	2846	1966	1524	4434	2720	2340	1510	2042	1910	2142	31066
ARO:3004035	tetA(60)	tetA(60) is a subunit of tetAB(60), an ABC transporter that confers resistance to tetracycline and tigercycline identified by screening a human saliva metagenomic library in Escherichia coli, which is required for resistance.	ATP-binding cassette (ABC) antibiotic efflux pump	tetracycline antibiotic	Tetracycline	Antibiotic efflux	4364	3380	1794	3628	2738	4042	4334	3958	2472	4142	3422	4932	43206
ARO:3004036	tetB(60)	tetB(60) is a subunit of tetAB(60), an ABC transporter that confers resistance to tetracycline and tigercycline identified by screening a human saliva metagenomic library in Escherichia coli, which is required for resistance.	ATP-binding cassette (ABC) antibiotic efflux pump	tetracycline antibiotic	Tetracycline	Antibiotic efflux	1696	1970	1326	1248	546	2484	1254	1436	790	3416	2830	4724	23720
ARO:3004038	Pseudomonas aerugin	EmrE is a small multidrug transporter that functions as a homodimer and that couples the efflux of small polyaromatic	small multidrug resistance (SMR)	aminoglycoside antibiotic	Multidrug	Antibiotic efflux	64	10	2	0	0	80	14	0	6	114	144	98	532

	osa emrE	cations from the cell with the import of protons down an electrochemical gradient. Confers resistance to tetraphenylphosphonium, methyl viologen, gentamicin, kanamycin, and neomycin.	antibiotic efflux pump																
ARO:3004039	Escherichia coli emrE	Member of the small MDR (multidrug resistance) family of transporters; in Escherichia coli this protein provides resistance against a number of positively charged compounds including ethidium bromide and erythromycin; proton-dependent secondary transporter which exchanges protons for compound translocation	small multidrug resistance (SMR) antibiotic efflux pump	macrolide antibiotic	MLS	Antibiotic efflux	608	1200	64	232	238	656	272	226	162	286	216	352	4512
ARO:3004041	Klebsiella pneumoniae acrA	AcrA is a subunit of the AcrAB multidrug efflux system that in K. pneumoniae, which is encoded by the acrRAB operon.	resistance-nodulation-cell division (RND) antibiotic efflux pump	cephalosporin;fluoroquinolone antibiotic;glycylcycline;penam;phenicol antibiotic;rifamycin antibiotic;tetracycline antibiotic;triclosan	Multidrug	Antibiotic efflux	6	0	4	4	2	0	12	0	18	90	132	162	430
ARO:3004042	Enterobacter cloacae acrA	AcrA is a subunit of the AcrAB-TolC multidrug efflux system that in E. cloacae.	resistance-nodulation-cell division (RND) antibiotic efflux pump	cephalosporin;fluoroquinolone antibiotic;glycylcycline;penam;phenicol antibiotic;rifamycin antibiotic;tetracycline antibiotic;triclosan	Multidrug	Antibiotic efflux	246	272	354	184	122	168	156	190	82	1786	1166	2616	7342
ARO:3004043	Escherichia coli acrA	AcrA is a subunit of the AcrAB-TolC multidrug efflux system that in E. coli.	resistance-nodulation-cell division (RND) antibiotic efflux pump	cephalosporin;fluoroquinolone antibiotic;glycylcycline;penam;phenicol antibiotic;rifamy	Multidrug	Antibiotic efflux	530	318	192	1094	428	112	1518	1312	394	350	604	466	7318

				cin antibiotic;tetracycline antibiotic;triclosan														
ARO:3004054	Pseudomonas aeruginosa CpxR	CpxR is directly involved in activation of expression of RND efflux pump MexAB-OprM in P. aeruginosa. CpxR is required to enhance mexAB-oprM expression and drug resistance, in the absence of repressor MexR.	resistance-nodulation-cell division (RND) antibiotic efflux pump	aminocoumarin antibiotic;aminoglycoside antibiotic;carbapenem;cephalosporin;cephamycin;diaminopyrimidine antibiotic;fluoroquinolone antibiotic;macrolide antibiotic;monobactam;penam;penem;peptide antibiotic;phenicol antibiotic;sulfonamide antibiotic;tetracycline antibiotic	Multidrug	Antibiotic efflux	2526	1176	2418	3336	5544	1628	4350	4504	5194	9872	9088	13550	63186
ARO:3004063	EdeQ	EdeQ is an N-acetyltransferase enzyme that confers high-level self-resistance to edeine in Brevibacillus brevis, a natural edeine producer. EdeQ converts active edeine to N-acetyledeine, which is ineffective in vivo.	Edeine acetyltransferase	peptide antibiotic;polyamine antibiotic	Multidrug	Antibiotic inactivation	4	14	10	0	16	22	0	2	14	4	14	8	108
ARO:3004072	OpmB	OpmB is an outer membrane efflux protein in Pseudomonas aeruginosa that shows functional cooperation with MuxABC, to form the efflux pump system MuxABC-OpmB.	resistance-nodulation-cell division (RND) antibiotic efflux pump	aminocoumarin antibiotic;macrolide antibiotic;monobactam;tetracycline antibiotic	Multidrug	Antibiotic efflux	384	218	1530	792	5122	348	948	1434	4680	3086	3478	4244	26264
ARO:3004073	MuxA	MuxA is a membrane fusion protein component of the efflux pump system MuxABC-OpmB in Pseudomonas aeruginosa.	resistance-nodulation-cell division (RND) antibiotic efflux pump	aminocoumarin antibiotic;macrolide antibiotic;monobactam;tetracycline antibiotic	Multidrug	Antibiotic efflux	964	1442	2668	1710	4938	1256	1898	1896	5158	2488	2452	2654	29524
ARO:3004074	MuxB	MuxB is one of the two necessary RND components in	resistance-nodulation-	aminocoumarin antibiotic;macrol	Multidrug	Antibiotic efflux	2096	2768	1836	1484	5594	1942	2292	2366	5300	4258	4650	5112	39698

		the Pseudomonas aeruginosa efflux pump system MuxABC-OpmB.	cell division (RND) antibiotic efflux pump	ide antibiotic;monobactam;tetracycline antibiotic															
ARO:3004075	MuxC	MuxC is one of the two necessary RND components of the MuxABC-OpmB efflux pumps system in Pseudomonas aeruginosa.	resistance-nodulation-cell division (RND) antibiotic efflux pump	aminocoumarin antibiotic;macrolide antibiotic;monobactam;tetracycline antibiotic	Multidrug	Antibiotic efflux	1080	1136	1718	446	668	912	656	492	816	1772	1946	2368	14010
ARO:3004077	PmpM	PmpM is a multidrug efflux pump belonging to the MATE family of Pseudomonas aeruginosa. PmpM is an H+ drug antiporter and is the first reported case of an H+ coupled efflux pump in the MATE family. PmpM confers resistance to fluoroquinolones, fradiomycin, benzalkonium chloride, chlorhexidine gluconate, ethidium bromide, tetraphenylphosphonium chloride (TPPCl), and rhodamine 6G.	multidrug and toxic compound extrusion (MATE) transporter	aminoglycoside antibiotic;benzalkonium chloride;fluoroquinolone antibiotic	Multidrug	Antibiotic efflux	340	284	674	350	1332	290	466	526	1436	3398	3094	4990	17180
ARO:3004085	lnuG	lnuG is a transposon-mediated lincosamide nucleotidyltransferase found in Enterococcus faecalis on Tn6260.	lincosamide nucleotidyltransferase (LNU)	lincosamide antibiotic	MLS	Antibiotic inactivation	176	266	118	64	118	162	140	104	94	1266	896	1300	4704
ARO:3004089	ANT(3'')-IIa	ANT(3'')-IIa is a aminoglycoside nucleotidyltransferase identified in Acinetobacter spp. via horizontal gene transfer mechanisms.	ANT(3'')	aminoglycoside antibiotic	Aminoglycoside	Antibiotic inactivation	430	1242	772	1630	4442	346	2220	2214	4246	8080	6810	11412	43844
ARO:3004090	ANT(3'')-IIb	ANT(3'')-IIb is a aminoglycoside nucleotidyltransferase identified in Acinetobacter spp. via horizontal gene transfer mechanisms.	ANT(3'')	aminoglycoside antibiotic	Aminoglycoside	Antibiotic inactivation	12	30	20	90	24	12	52	62	38	44	44	32	460
ARO:3004091	ANT(3'')-IIc	ANT(3'')-IIc is a aminoglycoside nucleotidyltransferase identified in Acinetobacter spp. via horizontal gene transfer mechanisms.	ANT(3'')	aminoglycoside antibiotic	Aminoglycoside	Antibiotic inactivation	710	744	20	24	24	566	28	32	18	36	50	60	2312
ARO:3004094	Erm(48)	Erm(48) is a macrolide-lincosamide-streptogramin	Erm 23S ribosomal	lincosamide antibiotic;macrol	MLS	Antibiotic target	156	174	70	12	104	134	36	22	122	0	12	6	848

		resistance gene identified on resistance plasmid pJW2311.	RNA methyltransferase	ide antibiotic;streptogramin antibiotic		alteration													
ARO:3004099	LpeA	LpeA is a subunit of the LpeAB efflux pump in Legionella pneumophila, which is homologous to AcrA in E. coli.	resistance-nodulation-cell division (RND) antibiotic efflux pump	macrolide antibiotic	MLS	Antibiotic efflux	348	536	560	360	342	318	216	398	418	1782	1056	2464	8798
ARO:3004100	LpeB	LpeB is a subunit of the LpeAB efflux pump in Legionella pneumophila, which is homologous to AcrB in E. coli.	resistance-nodulation-cell division (RND) antibiotic efflux pump	macrolide antibiotic	MLS	Antibiotic efflux	636	792	776	262	290	568	306	370	462	1090	826	924	7302
ARO:3004103	QepA2	QepA2 is a plasmid-mediated quinolone resistance pump found in an Escherichia coli isolate from France	major facilitator superfamily (MFS) antibiotic efflux pump	fluoroquinolone antibiotic	Fluoroquinolone	Antibiotic efflux	64	48	16	8	54	188	120	20	44	142	208	134	1046
ARO:3004107	Pseudomonas aeruginosa soxR	SoxR is a redox-sensitive transcriptional activator that induces expression of a small regulon that includes the RND efflux pump-encoding operon mexGHI-opmD. SoxR was shown to be activated by pyocyanin.	ATP-binding cassette (ABC) antibiotic efflux pump;major facilitator superfamily (MFS) antibiotic efflux pump;resistance-nodulation-cell division (RND) antibiotic efflux pump	acridine dye;cephalosporin;fluoroquinolone antibiotic;glycylcycline;penam;phenicol antibiotic;rifamycin antibiotic;tetracycline antibiotic;triclosan	Multidrug	Antibiotic efflux;antibiotic target alteration	342	200	166	634	354	110	920	950	396	472	532	514	5590
ARO:3004113	FosA7	fosA7 is an enzyme that confers resistance to fosfomycin in Escherichia coli by breaking the epoxide ring of the molecule.	fosfomycin thiol transferase	fosfomycin	Fosfomycin	Antibiotic inactivation	0	0	0	2	0	0	4	0	0	0	0	0	6
ARO:3004122	Klebsiella pneumo	Klebsiella pneumoniae outer membrane porin protein. Is preferentially detected in porin-deficient strains. Functional	General Bacterial Porin with reduced	carbapenem;cephalosporin;cephamycin;monobact	Beta-lactam	Reduced permeability to	2060	972	554	4038	1830	242	6176	4754	1488	692	680	312	23798

	niae OmpK37	characterization of this new porin revealed a narrower pore than those of porins OmpK35 and OmpK36, which did not allow penetration by certain beta-lactams. Also, when a resistant strain expresses porin OmpK37 is less susceptible to cefotaxime and cefoxitin than when it is expressing either OmpK36 or OmpK35.	permeability to beta-lactams	am;penam;penem		antibiotic													
ARO:3004123	Burkholderia pseudomallei Omp38	Heterologous expression of Burkholderia pseudomallei Omp38 (BpsOmp38) in Omp-deficient E. coli host cells lowers their permeability and in consequence, their antimicrobial susceptibility to penicillin G, cefoxitin, ceftazidime and imipenem.	General Bacterial Porin with reduced permeability to beta-lactams	carbapenem;cephalosporin;cephamycin;monobactam;penam;penem	Beta-lactam	Reduced permeability to antibiotic	0	0	460	174	230	2	294	182	104	96	134	234	1910
ARO:3004142	OprZ	OprZ is the outer membrane component of the AxyXY-OprZ efflux pump system in Achromobacter spp.	resistance-nodulation-cell division (RND) antibiotic efflux pump	aminoglycoside antibiotic;cephalosporin;fluoroquinolone antibiotic;macrolide antibiotic	Multidrug	Antibiotic efflux	14	20	132	120	62	14	276	142	158	224	296	300	1758
ARO:3004143	AxyX	AxyX is the inner membrane transporter of the AxyXY-OprZ efflux pump system in Achromobacter spp.	resistance-nodulation-cell division (RND) antibiotic efflux pump	aminoglycoside antibiotic;cephalosporin;fluoroquinolone antibiotic;macrolide antibiotic	Multidrug	Antibiotic efflux	16	6	56	64	40	6	192	62	26	354	354	356	1532
ARO:3004144	AxyY	AxyY is the periplasmic adaptor protein of the AxyXY-OprZ efflux pump system in Achromobacter spp.	resistance-nodulation-cell division (RND) antibiotic efflux pump	aminoglycoside antibiotic;cephalosporin;fluoroquinolone antibiotic;macrolide antibiotic	Multidrug	Antibiotic efflux	44	28	186	134	74	100	298	154	118	380	296	252	2064
ARO:3004146	cfrC	A cfr-like 23S rRNA methyltransferase shown to confer resistance to linezolid and phenicol antibiotics, including florfenicol and chloramphenicol, in Clostridium.	Cfr 23S ribosomal RNA methyltransferase	phenicol antibiotic	Phenicol	Antibiotic target alteration	3026	3084	8298	4922	2408	2398	4146	5458	3192	3344	2898	3164	46338
ARO:3004185	mecD	mecD is a PBP2a variant identified on a genomic resistance island in Macrococcus	methicillin resistant PBP2	carbapenem;cephalosporin;cepha	Beta-lactam	Antibiotic target	1062	1804	836	962	3610	1182	908	1280	3164	1404	1376	1500	19088

		caseolyticus. MecD confers resistance to methicillin and other beta-lactam antibiotics through the production of an alternative low-affinity PBP. First described by Schwendener et al. 2017 and identified from canine and bovine sources.		mycin;monobactam;penam		replacement													
ARO:3004191	APH(2'')-If	Aminoglycoside 2''-phosphotransferase identified from the gram-negative pathogen Campylobacter jejuni. APH(2'')-If was shown to confer resistance to 4,6-disubstituted antibiotics kanamycin, tobramycin, dibekacin, gentamicin and sisomicin through antibiotic phosphorylation. Described by Toth et al. 2013.	APH(2'')	aminoglycoside antibiotic	Aminoglycoside	Antibiotic inactivation	22	6	68	28	4	26	36	34	10	38	14	22	308
ARO:3004213	GOB-18	GOB-18 is a member of subclass B3 GOB family of beta-lactamases isolated from Elizabethkingia meningoseptica. GOB-18 is unlike other subclass B3 beta-lactamases as it is fully active against a broad range of beta-lactam substrates using a single Zn(II) ion in its active site.	GOB beta-lactamase	carbapenem;cephalosporin;penam	Beta-lactam	Antibiotic inactivation	14	96	44	22	14	38	16	34	16	170	182	190	836
ARO:3004239	NPS-1	NPS-1 is a plasmid-encoded class D beta-lactamases identified from two Pseudomonas aeruginosa isolates in 1986.	NPS beta-lactamase	cephalosporin;penam	Beta-lactam	Antibiotic inactivation	0	0	10	14	0	0	48	8	0	28	22	60	190
ARO:3004253	vanUG	vanUG is a vanG variant found in the vanG gene cluster.	glycopeptide resistance gene cluster;vanU	glycopeptide antibiotic	Glycopeptide	Antibiotic target alteration	386	468	446	236	202	454	230	394	224	154	126	86	3406
ARO:3004254	vanVB	vanVB is a vanV variant found in the vanB gene cluster. It is found in some but not all vanB operons in E. faecalis, suggesting the existence of varied gene compositions in vanB operons.	glycopeptide resistance gene cluster;vanV	glycopeptide antibiotic	Glycopeptide	Antibiotic target alteration	0	8	36	26	6	6	38	40	14	8	2	4	188

ARO:3004289	Vibrio cholerae varG	varG is an Ambler class B metallo-beta-lactamase found on the antibiotic resistance var regulon in Vibrio cholerae, along with an antibiotic efflux pump varABCDEF. These genes are organized as a regulon under the control of VarR transcriptional activator. VarG was shown to have beta-lactamase activity against penicillins, carbapenems and cephalosporins in-vitro. Described by Lin HV et al. 2017.	subclass B1 Vibrio cholerae varG beta-lactamase	carbapenem	Beta-lactam	Antibiotic inactivation	0	0	0	0	0	0	0	0	0	2	14	0	16
ARO:3004290	Escherichia coli ampC beta-lactamase	A class C ampC beta-lactamase (cephalosporinase) enzyme described in Escherichia coli shown clinically to confer resistance to penicillin-like and cephalosporin-class antibiotics.	ampC-type beta-lactamase	cephalosporin;penam	Beta-lactam	Antibiotic inactivation	330	158	112	816	418	44	1272	1042	304	124	102	62	4784
ARO:3004291	Rhodobacter sphaeroides ampC beta-lactamase	A periplasmic cephalosporinase described in Rhodobacter sphaeroides shown to contribute to resistance of beta-lactam antibiotics.	ampC-type beta-lactamase	cephalosporin;penam	Beta-lactam	Antibiotic inactivation	78	16	16	2	2	106	2	8	0	54	50	62	396
ARO:3004292	Laribacter hongkongensis ampC beta-lactamase	A chromosome-encoded class C beta-lactamase described in the pathogen Laribacter hongkongensis, often associated with gastroenteritis. Laribacter ampC has been shown to confer resistance to non-carbapenem beta-lactamases, as described by Lau et al. 2005.	ampC-type beta-lactamase	cephalosporin;penam	Beta-lactam	Antibiotic inactivation	0	0	0	0	0	0	4	0	0	4	16	4	28
ARO:3004294	BUT-1	A chromosome-encoded class C cephalosporinase and penicillinase from Buttiauxella spp. shown clinically to confer resistance to beta-lactam antibiotics. Described by Fihman et al. 2002.	BUT beta-lactamase	cephalosporin	Beta-lactam	Antibiotic inactivation	0	4	20	14	0	0	6	16	0	70	114	96	340
ARO:3004325	MCR-4.1	MCR-4 is a plasmid-borne phosphoethanolamine transferase variant of MCR-1,	MCR phosphoeth	peptide antibiotic	Peptide	Antibiotic target	22	14	0	4	0	0	6	10	0	2	6	28	92

		isolated from Salmonella enterica serovar Typhimurium of porcine origin in Italy, Spain and Belgium through 2013 and 2015-2016. MCR-4 confers resistance to collistin via addition of a phosphoethanolamine group to lipid A, reducing the negative charge of the cell membrane. Described by Carattoli et al. 2017.	anolamine transferase			alteration												
ARO:3004332	MCR-5.1	MCR-5 is a transposon-associated phosphoethanolamine transferase gene, identified in Salmonella Paratyphi B dTa+ (d-tartrate fermenting Salmonella enterica subsp. enterica serovar Paratyphi B) isolates from food-producing animals. The isolates were collected between 2011 and 2013, and retrieved from the German National Reference Laboratory for Salmonella. MCR-5 confers resistance to collistin through the addition of a phosphoethanolamine group to lipid A, causing a reduction in negative charge of the cell membrane. Described by Borowiak et al, 2017.	MCR phosphoethanolamine transferase	peptide antibiotic	Peptide	Antibiotic target alteration	74	50	168	118	44	94	230	142	56	118	88	54	1236
ARO:3004336	PDC-73	An AmpC-like beta-lactamase found in Pseudomonas aeruginosa.	PDC beta-lactamase	carbapenem;cephalosporin;monobactam	Beta-lactam	Antibiotic inactivation	0	0	0	0	6	0	0	0	14	0	6	0	26
ARO:3004337	PDC-74	An AmpC-like beta-lactamase found in Pseudomonas aeruginosa	PDC beta-lactamase	carbapenem;cephalosporin;monobactam	Beta-lactam	Antibiotic inactivation	0	2	0	0	0	0	6	0	0	0	0	0	8
ARO:3004338	PDC-75	An AmpC-like beta-lactamase found in Pseudomonas aeruginosa	PDC beta-lactamase	carbapenem;cephalosporin;monobactam	Beta-lactam	Antibiotic inactivation	0	0	12	0	0	0	0	0	0	50	78	98	238
ARO:3004353	PDC-90	An AmpC-like beta-lactamase found in Pseudomonas aeruginosa	PDC beta-lactamase	carbapenem;cephalosporin;monobactam	Beta-lactam	Antibiotic	0	0	2	0	0	2	2	0	0	0	0	0	6

						inactivation													
ARO:3004354	PDC-91	An AmpC-like beta-lactamase found in Pseudomonas aeruginosa	PDC beta-lactamase	carbapenem;cephalosporin;monobactam	Beta-lactam	Antibiotic inactivation	28	6	0	2	2	10	2	0	2	2	0	0	54
ARO:3004357	catV	A chloramphenicol acetyltransferase identified in Brevibacillus brevis and shown to confer resistance to chloramphenicol antibiotics. Described by Pawlowski et al. 2017.	chloramphenicol acetyltransferase (CAT)	phenicol antibiotic	Phenicol	Antibiotic inactivation	8	24	28	24	14	38	6	22	8	4	6	6	188
ARO:3004359	ACI-1	A class A beta-lactamase described in Acidaminococcus fermentans. ACI-1 was the first description of a beta-lactamase enzyme in a gram-negative anaerobic cocci. Described by Galan et al 2000.	ACI beta-lactamase	cephalosporin;penam;penem	Beta-lactam	Antibiotic inactivation	160	216	312	146	288	184	206	154	332	48	24	14	2084
ARO:3004361	sul4	sul4 is a dihydropteroate synthase gene and mobile sulfonamide resistance gene shown to confer resistance when expressed in E. coli.	sulfonamide resistant sul	sulfonamide antibiotic	Sulfonamide	Antibiotic target replacement	3648	4156	3276	2952	4620	3356	3688	3308	4556	5894	6028	7688	53170
ARO:3004379	QepA4	A plasmid-mediated quinolone efflux pump variant described in Escherichia coli. QepA4 confers resistance to quinolone and fluoroquinolone antibiotics by expulsion from the cell. Described by Manageiro et al. 2017.	major facilitator superfamily (MFS) antibiotic efflux pump	fluoroquinolone antibiotic	Fluoroquinolone	Antibiotic efflux	12	32	14	36	6	26	94	42	10	1836	1098	2578	5784
ARO:3004441	tet(59)	Tet(59) is a chromosome-encoded tetracycline efflux pump described from a Chinese pig manure sample.	major facilitator superfamily (MFS) antibiotic efflux pump	tetracycline antibiotic	Tetracycline	Antibiotic efflux	0	34	510	260	2292	8	494	742	1910	1658	1562	2008	11478
ARO:3004442	tet(W/N/W)	tet(W/N/W) is a mosaic tetracycline resistance gene and ribosomal protection protein.	tetracycline-resistant ribosomal protection protein	tetracycline antibiotic	Tetracycline	Antibiotic target protection	1664	2478	2220	926	952	1976	946	1064	1176	628	414	258	14702
ARO:3004451	Agrobacterium fabrum	A chloramphenicol resistance determinant described in the	chloramphenicol	phenicol antibiotic	Phenicol	Antibiotic	1862	710	1020	1944	966	922	2856	2302	1042	658	670	660	15612

	chloramphenicol acetyltransferase	Gram-negative bacterium Agrobacterium fabrum.	acetyltransferase (CAT)			inactivation													
ARO:3004454	Campylobacter coli chloramphenicol acetyltransferase	A chloramphenicol resistance determinant described in Campylobacter coli.	chloramphenicol acetyltransferase (CAT)	phenicol antibiotic	Phenicol	Antibiotic inactivation	326	156	566	206	152	216	114	212	150	52	8	46	2204
ARO:3004455	Streptococcus suis chloramphenicol acetyltransferase	A chloramphenicol resistance determinant described in Streptococcus suis.	chloramphenicol acetyltransferase (CAT)	phenicol antibiotic	Phenicol	Antibiotic inactivation	0	2	2	0	0	2	0	0	0	12	6	6	30
ARO:3004457	Staphylococcus intermedius chloramphenicol acetyltransferase	A chloramphenicol resistance determinant described in Staphylococcus intermedius	chloramphenicol acetyltransferase (CAT)	phenicol antibiotic	Phenicol	Antibiotic inactivation	0	2	38	0	60	0	0	0	52	70	78	92	392
ARO:3004460	Vibrio anguillarum chloramphenicol acetyltransferase	A chloramphenicol resistance determinant described in a Vibrio anguillarum plasmid sequence	chloramphenicol acetyltransferase (CAT)	phenicol antibiotic	Phenicol	Antibiotic inactivation	4	6	6	4	42	20	6	10	36	0	0	0	134
ARO:3004466	ICR-Mc	A chromosomally-encoded colistin resistance phosphoethanolamine (PEtN) transferase of Moraxella catarrhalis. ICR-Mc is the protein that represents the closest known ortholog to the colistin resistance MCR-1 and MCR-2 PEtN transferases.	intrinsic colistin resistant phosphoethanolamine transferase	peptide antibiotic	Peptide	Antibiotic target alteration	0	0	4	12	0	0	10	2	0	2	2	0	32

ARO:3004470	poxtA	PoxtA is an ABC-F subfamily ATP-binding cassette protein that confers resistance to tetracycline, -phenicol, and oxazolidinone via modification of the bacterial ribosome. The encoding gene was isolated from a methicillin-resistant Staphylococcus aureus strain.	ABC-F ATP-binding cassette ribosomal protection protein	lincosamide antibiotic;macrolide antibiotic;oxazolidinone antibiotic;phenicol antibiotic;pleuromutilin antibiotic;streptogramin antibiotic;tetracycline antibiotic	Multidrug	Antibiotic target protection	1892	1250	1296	598	390	2432	696	622	604	954	950	682	12366
ARO:3004476	vmlR	vmlR is an ABC-F ATPase ribosomal protection protein identified in Bacillus subtilus. Shown to confer resistance to lincomycin and streptogramin A virginiamycin. Described by Crowe-McAuliffe et al. 2018.	ABC-F ATP-binding cassette ribosomal protection protein	lincosamide antibiotic;macrolide antibiotic;oxazolidinone antibiotic;phenicol antibiotic;pleuromutilin antibiotic;streptogramin antibiotic;tetracycline antibiotic	Multidrug	Antibiotic target protection	3178	2660	1924	2678	4020	2624	3308	3448	4042	2080	2196	2336	34494
ARO:3004478	OXA-665	Beta-lactamase found in Acinetobacter rudis efficiently inactivating carbapenems and amoxicillin conferring resistance to cephalosporins	OXA beta-lactamase	cephalosporin;penam	Beta-lactam	Antibiotic inactivation	0	0	12	0	18	0	0	0	0	6	0	14	50
ARO:3004480	Bifidobacterium adolescentis rpoB mutants conferring resistance to rifampicin	Bifidobacterium are antibiotic resistant probiotics are prescribed to upkeep the population beneficial bacteria in the gut microbiome. However, horizontal gene transfer among gut microbes could create harmful antibiotic-resistant pathogenic bacteria, such as Mycobacterium tuberculosis. Lokesh et al. analyzed Bifidobacterium antitubercular drug resistance and mutations in rpoB. They found that B. animalis, B. longum and B. adolescentis showed	rifamycin-resistant beta-subunit of RNA polymerase (rpoB)	peptide antibiotic;rifamycin antibiotic	Multidrug	Antibiotic target alteration;antibiotic target replacement	2214	2520	3516	1706	6384	2320	2270	3082	7512	3308	3310	2990	41132

		considerable resistance to pyrazinamide, isoniazid, and streptomycin, while B. adolescentis had mutations both in the rifampicin (RIF) pocket and in regions outside the pockets, and also showed considerable resistance to RIF.																
ARO:3004500	MCR-3.11	An MCR-3 phosphoethanolamine transferase and polymyxin resistance gene variant differing by 2 amino acid substitutions, identified from an Escherichia coli isolate.	MCR phosphoethanolamine transferase	peptide antibiotic	Peptide	Antibiotic target alteration	0	0	34	12	6	0	10	16	6	8	0	0	92
ARO:3004501	MCR-6.1	An MCR-2 phosphoethanolamine transferase and polymyxin resistance gene variant identified in Moraxella isolated from pigs in the United Kingdom.	MCR phosphoethanolamine transferase	peptide antibiotic	Peptide	Antibiotic target alteration	2	22	2	0	0	44	0	0	6	0	0	0	76
ARO:3004502	MCR-2.2	An MCR-2 phosphoethanolamine transferase and polymyxin resistance gene variant identified in Moraxella isolated from pigs in Great Britain	MCR phosphoethanolamine transferase	peptide antibiotic	Peptide	Antibiotic target alteration	0	0	0	2	0	0	2	0	0	0	0	0	4
ARO:3004506	MCR-1.10	An MCR-1 phosphoethanolamine transferase and polymyxin (colistin) resistance gene variant identified from an Escherichia porcine isolate in Great Britain.	MCR phosphoethanolamine transferase	peptide antibiotic	Peptide	Antibiotic target alteration	0	0	0	2	0	0	0	2	0	0	0	0	4
ARO:3004508	MCR-3.9	An MCR-3 polymyxin (incl. colistin) resistance gene variant isolated from an Aeromonas isolate	MCR phosphoethanolamine transferase	peptide antibiotic	Peptide	Antibiotic target alteration	0	8	0	6	0	10	12	0	0	0	0	0	36
ARO:3004510	MCR-3.7	An MCR polymyxin (colistin) resistance gene variant identified in Aeromonas	MCR phosphoethanolamine transferase	peptide antibiotic	Peptide	Antibiotic target alteration	4	20	4	14	0	6	2	2	0	0	0	0	52
ARO:3004514	MCR-1.3	An MCR-1 polymyxin resistance gene variant identified from a poultry Raoultella planticola isolate	MCR phosphoethanolamine transferase	peptide antibiotic	Peptide	Antibiotic target alteration	0	0	0	0	0	0	0	0	0	4	20	4	28

ARO:3004516	MCR-8.1	A novel phosphoethanolamine transferase and mobile colistin resistance gene identified from carbapenem-resistant NDM-1-producing Klebsiella pneumoniae.	MCR phosphoethanolamine transferase	peptide antibiotic	Peptide	Antibiotic target alteration	146	110	74	26	8	94	94	58	18	18	42	70	758
ARO:3004517	MCR-7.1	A novel plasmid-mediated colistin-resistant phosphoethanolamine transferase identified from a poultry isolate of Klebsiella pneumoniae	MCR phosphoethanolamine transferase	peptide antibiotic	Peptide	Antibiotic target alteration	26	64	8	68	0	74	104	90	14	30	46	62	586
ARO:3004539	mphH	A chromosomal macrolide 2'-phosphotransferase and resistance gene identified from a Brachybacterium faecium cave isolate	macrolide phosphotransferase (MPH)	macrolide antibiotic	MLS	Antibiotic inactivation	0	0	0	0	26	0	0	0	30	16	52	100	224
ARO:3004543	mphO	A chromosomal macrolide phosphostransferase identified in Brachybacterium paraconglomeratum	macrolide phosphotransferase (MPH)	macrolide antibiotic	MLS	Antibiotic inactivation	0	0	0	0	6	0	0	0	0	0	4	8	18
ARO:3004548	dfrA9	A dihydrofolate reductase and trimethoprim resistance gene identified from porcine isolates of Esherichia coli	trimethoprim resistant dihydrofolate reductase dfr	diaminopyrimidine antibiotic	Diaminopyrimidine	Antibiotic target replacement	2	8	6	30	8	12	58	102	8	50	26	124	434
ARO:3004551	dfrA28	A dihydrofolate reductase and trimethoprim resistance gene from Aeromonas hydrophilia	trimethoprim resistant dihydrofolate reductase dfr	diaminopyrimidine antibiotic	Diaminopyrimidine	Antibiotic target replacement	0	4	2	0	2	0	6	2	4	10	14	36	80
ARO:3004568	dfrA18	A trimethoprim-resistant dihydrofolate reductase identified from Vibrio cholerae	trimethoprim resistant dihydrofolate reductase dfr	diaminopyrimidine antibiotic	Diaminopyrimidine	Antibiotic target replacement	28	16	48	4	2	48	6	2	12	2	10	24	202
ARO:3004569	ICR-Mo	A chromosomally-encoded colistin resistance phosphoethanolamine (PEtN) transferase of Moraxella osloensis. ICR-Mo represents the closest known ortholog to the colistin resistance MCR-1 and MCR-2 PEtN transferases	intrinsic colistin resistant phosphoethanolamine transferase	peptide antibiotic	Peptide	Antibiotic target alteration	36	0	0	2	8	10	0	6	4	0	0	0	66
ARO:3004572	Staphylococcus	MFS transporters are secondary active transporters with single-polypeptide chains containing	major facilitator superfamily	aminoglycoside antibiotic;diaminopyrimidine	Multidrug	Antibiotic efflux	600	242	78	8	98	1010	16	18	180	72	118	116	2556

	aureus LmrS	400-600 amino acids that transport small solutes across the membrane by using electrochemical gradients. LmrS has 14 transmembrane helices and, when expressed in E. coli, is capable of extruding a variety of antibiotics inclinding linezolid, trimethoprim, florfenicol, chlorampheniocol, erythromycin, streptomycin, kanamycin, and fusidic acid	(MFS) antibiotic efflux pump	antibiotic;macrolide antibiotic;oxazolidinone antibiotic;phenicol antibiotic															
ARO:3004573	Acinetobacter baumannii AbaF	Expression of abaF in E. coli resulted in increased resistance to fosfomycin.	major facilitator superfamily (MFS) antibiotic efflux pump	fosfomycin	Fosfomycin	Antibiotic efflux	2722	868	606	2954	1438	1718	4742	3266	1634	1740	2362	1690	25740
ARO:3004574	Acinetobacter baumannii AbaQ	AbaQ is an MFS transporter mainly involved in the extrusion of quinolone-type drugs in A. baumannii	major facilitator superfamily (MFS) antibiotic efflux pump	fluoroquinolone antibiotic	Fluoroquinolone	Antibiotic efflux	1166	610	204	842	346	686	1100	814	348	156	140	106	6518
ARO:3004577	Acinetobacter baumannii AmvA	AmvA has 14 alpha-helical transmembrane segments, qualifying it as a member of the DHA2 transporter family of the major facilitator superfamily (MFS). When AmvA was expressed in E. coli, the cells had at least four-fold decreased susceptibility to erythromycin, acridine orange, acriflavine, deoxycholate and methyl viologen. AmvA from the AC0037 strain of A. baumannii was tested.	major facilitator superfamily (MFS) antibiotic efflux pump	acridine dye;macrolide antibiotic	Multidrug	Antibiotic efflux	528	22	168	116	114	686	66	152	126	80	112	120	2290
ARO:3004580	Klebsiella pneumoniae KpnE	KpnE subunit of KpnEF resembles EbrAB from E. coli. Mutation in KpnEF resulted in increased susceptibility to cefepime, ceftriaxon, colistin, erythromycin, rifampin, tetracycline, and streptomycin as well as enhanced sensitivity toward sodium dodecyl sulfate,	major facilitator superfamily (MFS) antibiotic efflux pump	aminoglycoside antibiotic;cephalosporin;macrolide antibiotic;peptide antibiotic;rifamycin	Multidrug	Antibiotic efflux	238	58	56	320	128	10	556	374	86	440	458	666	3390

		deoxycholate, dyes, benzalkonium chloride, chlorhexidine, and triclosan		antibiotic;tetracycline antibiotic														
ARO:3004583	Klebsiella pneumoniae KpnF	KpnF subunit of KpnEF resembles EbrAB from E. coli. Mutation in KpnEF resulted in increased susceptibility to cefepime, ceftriaxon, colistin, erythromycin, rifampin, tetracycline, and streptomycin as well as enhanced sensitivity toward sodium dodecyl sulfate, deoxycholate, dyes, benzalkonium chloride, chlorhexidine, and triclosan.	major facilitator superfamily (MFS) antibiotic efflux pump	aminoglycoside antibiotic;cephalosporin;macrolide antibiotic;peptide antibiotic;rifamycin antibiotic;tetracycline antibiotic	Multidrug	Antibiotic efflux	134	36	48	324	106	8	460	336	84	58	92	40	1726
ARO:3004586	tet(51)	A tetracycline inactivating enzyme. A flavoenzyme capable of degrading tetracycline antibiotics.	tetracycline inactivation enzyme	tetracycline antibiotic	Tetracycline	Antibiotic inactivation	0	6	48	0	104	0	50	0	30	172	176	320	906
ARO:3004587	tet(52)	A tetracycline inactivating enzyme. A flavoenzyme capable of degrading tetracycline antibiotics.	tetracycline inactivation enzyme	tetracycline antibiotic	Tetracycline	Antibiotic inactivation	0	0	0	0	0	0	12	0	0	0	2	0	14
ARO:3004588	Klebsiella pneumoniae KpnG	KpnG consists of ~390 residues and resembles EmrA of E. coli. Disruption of the pump components KpnG-KpnH signficantly decrease resistance to azithromycin, ceftazidime, ciprofloxacin, ertapenem, erythromycin, gentamicin, imipenem, ticarcillin, norfloxacin, polymyxin-B, piperacillin, spectinomycin, tobramycin, and streptomycin	major facilitator superfamily (MFS) antibiotic efflux pump	aminoglycoside antibiotic;carbapenem;cephalosporin;fluoroquinolone antibiotic;macrolide antibiotic;penam;penem;peptide antibiotic	Multidrug	Antibiotic efflux	80	16	68	30	46	78	60	32	40	0	12	18	480
ARO:3004589	tet(53)	A tetracycline inactivating enzyme. A flavoenzyme capable of degrading tetracycline antibiotics.	tetracycline inactivation enzyme	tetracycline antibiotic	Tetracycline	Antibiotic inactivation	0	2	14	74	4	0	28	60	2	0	0	0	184
ARO:3004590	tet(54)	A tetracycline inactivating enzyme. A flavoenzyme capable of degrading tetracycline antibiotics.	tetracycline inactivation enzyme	tetracycline antibiotic	Tetracycline	Antibiotic inactivation	16	0	0	0	0	0	54	6	0	0	0	0	76
ARO:3004591	tet(55)	A tetracycline inactivating enzyme. A flavoenzyme capable of degrading tetracycline antibiotics.	tetracycline inactivation enzyme	tetracycline antibiotic	Tetracycline	Antibiotic inactivation	14	6	20	10	34	12	50	42	60	242	488	358	1336

ARO:3004592	erm(32)	erm(32) is a rRNA methyltransferase. Encodes methyltransferases that modify 23S rRNA.	Erm 23S ribosomal RNA methyltransferase	lincosamide antibiotic;macrolide antibiotic;streptogramin antibiotic	MLS	Antibiotic target alteration	34	38	14	4	28	46	18	4	16	24	32	32	290
ARO:3004595	erm(45)	Erm45 is an rRNA methylase that confers resistances to macrolide, lincosamide, and streptogramin B.	Erm 23S ribosomal RNA methyltransferase	lincosamide antibiotic;macrolide antibiotic;streptogramin antibiotic	MLS	Antibiotic target alteration	0	0	0	0	0	0	0	0	0	6	0	0	6
ARO:3004596	erm(46)	erm(46) is transferable determinant that confers resistance to macrolides.	Erm 23S ribosomal RNA methyltransferase	lincosamide antibiotic;macrolide antibiotic;streptogramin antibiotic	MLS	Antibiotic target alteration	0	70	28	18	20	22	34	32	26	36	6	30	322
ARO:3004597	Klebsiella pneumoniae KpnH	KpnH consists of ~511 residues, resembles EmrB of E. coli, and is probably a translocase in the KpnGH-TolC efflux protein in K. pneumoniae. Disruption of the pump components KpnG-KpnH signficantly decrease resistance to azithromycin, ceftazidime, ciprofloxacin, ertapenem, erythromycin, gentamicin, imipenem, ticarcillin, norfloxacin, polymyxin-B, piperacillin, spectinomycin, tobramycin, and streptomycin.	major facilitator superfamily (MFS) antibiotic efflux pump	aminoglycoside antibiotic;carbapenem;cephalosporin;fluoroquinolone antibiotic;macrolide antibiotic;penam;penem;peptide antibiotic	Multidrug	Antibiotic efflux	0	4	0	16	0	0	58	10	0	6	18	24	136
ARO:3004599	clcD	clcD gene can provide resistance to representatives of five of the six antibiotic groups previously shown to be affected by Cfr. clcD was originally found in Clostridioides difficile and is cfr-like.	Cfr 23S ribosomal RNA methyltransferase	lincosamide antibiotic;oxazolidinone antibiotic;phenicol antibiotic;pleuromutilin antibiotic;streptogramin antibiotic	Multidrug	Antibiotic target alteration	66	32	52	10	12	30	28	24	14	76	102	30	476
ARO:3004601	LnuP	LnuP is a lincosamide nucleotidyltransferase major efflux facilitator	lincosamide nucleotidyltransferase (LNU)	lincosamide antibiotic	MLS	Antibiotic inactivation	2	16	0	4	4	6	0	0	18	72	84	10	216
ARO:3004603	tet(56)	A tetracycline inactivating enzyme. A flavoenzyme capable of degrading tetracycline antibiotics.	tetracycline inactivation enzyme	tetracycline antibiotic	Tetracycline	Antibiotic inactivation	0	0	0	0	0	20	0	2	0	0	0	0	22

ARO:3004606	erm(40)	erm(40) is an adenine RNA methylase and confers intrinsic resistance in Mycobacteria.	Erm 23S ribosomal RNA methyltransferase	lincosamide antibiotic;macrolide antibiotic;streptogramin antibiotic	MLS	Antibiotic target alteration	0	0	0	6	0	0	10	6	0	32	58	14	126
ARO:3004608	EreD	erythromycin esterase protein	macrolide esterase	macrolide antibiotic	MLS	Antibiotic inactivation	2	6	134	84	100	10	98	226	84	1098	750	1274	3866
ARO:3004611	Escherichia coli ampC1 beta-lactamase	An ampC-like beta-lactamase identified from Escherichia coli.	ampC-type beta-lactamase	cephalosporin;penam	Beta-lactam	Antibiotic inactivation	1184	864	1218	956	642	650	1244	1156	670	590	520	490	10184
ARO:3004612	Escherichia coli ampH beta-lactamase	AmpH is a class C ampC-like beta-lactamase and penicillin-binding protein identified in Escherichia coli.	ampC-type beta-lactamase	cephalosporin;penam	Beta-lactam	Antibiotic inactivation	660	454	376	1192	466	360	1614	1364	462	362	338	264	7912
ARO:3004623	AAC(3)-IId	AAC(3)-IId is a plasmid-encoded aminoglycoside acetyltransferase in E. coli	AAC(3)	aminoglycoside antibiotic	Aminoglycoside	Antibiotic inactivation	234	1126	46	456	430	358	534	492	332	264	224	344	4840
ARO:3004624	QnrS11	QnrS11 is a plasmid-mediated quinolone resistance protein.	quinolone resistance protein (qnr)	fluoroquinolone antibiotic	Fluoroquinolone	Antibiotic target protection	206	2	0	4	0	44	2	6	0	0	0	0	264
ARO:3004626	Erm(49)	Erm(49) is an rRNA methylase gene	Erm 23S ribosomal RNA methyltransferase	lincosamide antibiotic;macrolide antibiotic;streptogramin antibiotic	MLS	Antibiotic target alteration	278	362	350	260	98	408	212	188	108	178	110	122	2674
ARO:3004629	AAC(6')-Im	AAC(6')-Im is an aminoglycoside acetyltransferase encoded by plasmids in E. coli, and E. faecium	AAC(6')	aminoglycoside antibiotic	Aminoglycoside	Antibiotic inactivation	22	36	96	94	56	114	72	90	76	6	4	8	674
ARO:3004636	qnrE1	qnrE1 is a member of the qnrE family of plasmid-borne (fluoro)quinolone-resistance genes, with chromosomal origins from Enterobacter spp.	quinolone resistance protein (qnr)	fluoroquinolone antibiotic	Fluoroquinolone	Antibiotic target protection	0	0	0	2	2	0	2	14	0	22	18	18	78
ARO:3004639	Corynebacterium	tetAB genes of the Corynebacterium striatum R-plasmid which encode an ABC transporter and confer	major facilitator superfamily (MFS)	penam;tetracycline antibiotic	Multidrug	Antibiotic efflux	2074	1838	1490	1790	1016	2048	2446	2060	1310	2154	2860	2150	23236

	striatum tetA	tetracycline, oxytetracycline, and oxalic resistance	antibiotic efflux pump																
ARO:3004642	dfrA3b	A dihydrofolate reductase that confers resistance to trimethoprim	trimethoprim resistant dihydrofolate reductase dfr	diaminopyrimidine antibiotic	Diaminopyrimidine	Antibiotic target replacement	0	4	0	0	0	6	0	0	0	2	6	0	18
ARO:3004643	Erm(K)	23S rRNA (adenine(2058)-N(6))-methyltransferase Erm(K) [Bacillus halodurans]	Erm 23S ribosomal RNA methyltransferase	lincosamide antibiotic;macrolide antibiotic;streptogramin antibiotic	MLS	Antibiotic target alteration	408	1028	458	374	250	570	414	394	296	106	136	70	4504
ARO:3004644	dfrA6 from Proteus mirabilis	dfrA6 is a dihydrofolate reductase that confers resistance to trimethoprim.	trimethoprim resistant dihydrofolate reductase dfr	diaminopyrimidine antibiotic	Diaminopyrimidine	Antibiotic target replacement	2	0	10	2	60	2	2	0	30	152	144	300	704
ARO:3004645	dfrI	dfrI is a dihydrofolate reductase that is resistant to trimethoprim.	trimethoprim resistant dihydrofolate reductase dfr	diaminopyrimidine antibiotic	Diaminopyrimidine	Antibiotic target replacement	28	202	522	122	98	100	64	104	124	88	122	138	1712
ARO:3004648	AQU-3	AQU-3 is a chromosomally-encoded AQU class C beta-lactamase and cephalosporinase from	AQU beta-lactamase	cephalosporin	Beta-lactam	Antibiotic inactivation	0	0	0	26	0	0	66	18	0	14	2	2	128
ARO:3004649	cfr(B)	cfr(B) has been observed in mobile genetic elements in E. faecium and Clostridioides difficile and confers resistance to linezolid, clindamycin, erythromycin, chloramphenicol, and retapamulin.	Cfr 23S ribosomal RNA methyltransferase	lincosamide antibiotic;oxazolidinone antibiotic;phenicol antibiotic	Multidrug	Antibiotic target alteration	666	1006	854	416	404	816	410	444	398	432	358	310	6514
ARO:3004650	tetU	Tetracycline-resistant determinant encoded on the plasmid pKQ10 in Enterococcus faecium	major facilitator superfamily (MFS) antibiotic efflux pump	tetracycline antibiotic	Tetracycline	Antibiotic efflux	26	4	8	0	102	56	0	0	96	2	0	0	294
ARO:3004651	lin	Listeria monocytogenes EGD-e lin gene for lincomycin resistance ABC-F type ribosomal protection protein, complete CDS.	lincosamide nucleotidyltransferase (LNU)	lincosamide antibiotic	MLS	Antibiotic inactivation	66	236	164	128	104	186	114	168	190	274	262	294	2186
ARO:3004652	Erm(O)-lrm	ErmO (gene lrm) lincomycin resistance methylase [Streptomyces lividans].	Erm 23S ribosomal RNA	lincosamide antibiotic;macrolide	MLS	Antibiotic target	0	0	0	0	0	0	0	0	0	0	2	24	26

			methyltransferase	antibiotic;streptogramin antibiotic		alteration													
ARO:3004656	catII from Escherichia coli K-12	catII is a chloramphenicol acetyltransferase. This particular catII is found in E.coli K-12. Confers resistance to chloramphenicol.	chloramphenicol acetyltransferase (CAT)	phenicol antibiotic	Phenicol	Antibiotic inactivation	0	82	16	0	2	16	4	4	2	0	0	0	126
ARO:3004657	catA4	catA4 is a chloramphenicol acetyltransferase that confers resistance to chloramphenicol.	chloramphenicol acetyltransferase (CAT)	phenicol antibiotic	Phenicol	Antibiotic inactivation	6	4	10	32	14	8	12	12	2	2	4	4	110
ARO:3004658	catA8	catA8 is a chloramphenicol acetyltransferase that confers resistance to chloramphenicol.	chloramphenicol acetyltransferase (CAT)	phenicol antibiotic	Phenicol	Antibiotic inactivation	0	0	0	2	0	0	4	2	0	22	10	16	56
ARO:3004659	Mef(En2)	NBU2-encoded resistance gene. An MefE homolog in Bacteroides species. Macrolide efflux MFS transporter	major facilitator superfamily (MFS) antibiotic efflux pump	macrolide antibiotic	MLS	Antibiotic efflux	100	60	232	30	20	76	40	54	44	36	20	12	724
ARO:3004660	catB11	catB11 is a chloramphenicol acetyltransferase that confers resistance to chloramphenicol.	chloramphenicol acetyltransferase (CAT)	phenicol antibiotic	Phenicol	Antibiotic inactivation	6	102	556	404	3120	28	594	1156	2976	1736	1940	2120	14738
ARO:3004662	MCR-3.3	MCR-3.3 is a colistin resistance gene variant in the chromosome of an Aeromonas veronii. Conferred polymyxin resistance in both E. Coli and Aeromonas salmonicida	MCR phosphoethanolamine transferase	peptide antibiotic	Peptide	Antibiotic target alteration	0	2	0	2	0	10	6	0	0	2	12	0	34
ARO:3004663	FusF	A fusidic acid resistance determinant in Staphylococcus cohnii. FusD-like	fusidic acid inactivation enzyme	fusidic acid	Fusidic acid	Antibiotic inactivation	2	2	0	2	0	28	0	2	0	0	0	0	36
ARO:3004665	cmlA8	cmlA8 is a plasmid that confers resistance to chloramphenicol.	major facilitator superfamily (MFS) antibiotic efflux pump	phenicol antibiotic	Phenicol	Antibiotic efflux	0	0	0	4	0	0	18	20	0	14	16	56	128
ARO:3004666	pexA	pexA is a florfenicol and chloramphenicol resistance gene discovered in Alaskan soil.	major facilitator superfamily (MFS) antibiotic efflux pump	phenicol antibiotic	Phenicol	Antibiotic efflux	0	0	6	0	0	0	0	0	0	26	34	8	74

ARO:3004667	Staphylococcus aureus norA	NorA gene cloned from Staphylococcus aureus conferred relatively high resistance to hydrophilic quinolones such as norfloxacin, enoxacin, ofloxacin, and ciprofloxacin in S. aureus and Escherichia coli. Had low or no resistance at all to hydrophobic ones such as nalidixic acid, oxolinic acid, and sparfloxacin in S. aureus and Escherichia coli.	major facilitator superfamily (MFS) antibiotic efflux pump	fluoroquinolone antibiotic	Fluoroquinolone	Antibiotic efflux	182	16	34	2	196	162	8	2	160	296	268	474	1800
ARO:3004675	aphA15	Expression of cloned aphA15 gene in Escherichia coli reduced the susceptibility to kanamycin and neomycin, as well as to amikacin, netilmicin, and streptomycin	APH(3')	aminoglycoside antibiotic	Aminoglycoside	Antibiotic inactivation	0	0	0	0	0	0	2	4	6	70	94	88	264
ARO:3004680	APH(3')-VIIIa	APH(3')-VIIIa is an aminoglycoside phosphoryltransferase that acts on the 3-OH target of aminoglycosides found in Streptomyces rimosus.	APH(3')	aminoglycoside antibiotic	Aminoglycoside	Antibiotic inactivation	0	0	0	0	0	0	0	0	0	4	18	0	22
ARO:3004682	aadA27	Small mobilizable plasmid pALWED1.8 containing a novel variant of the streptomycin/spectinomycin resistance gene	ANT(3'')	aminoglycoside antibiotic	Aminoglycoside	Antibiotic inactivation	162	1832	4	34	26	410	22	54	38	62	58	84	2786
ARO:3004683	aadS	a novel Tn4551 streptomycin-resistance gene that was phenotypically silent in wild-type Bacteroides; expression could be activated by a trans-acting chromosomal mutation	ANT(6)	aminoglycoside antibiotic	Aminoglycoside	Antibiotic inactivation	22	50	244	32	120	70	176	52	106	1230	1178	1348	4628
ARO:3004684	MCR-9.1	A mobilized and plasmid-mediated colistin resistance gene and phosphoethanolamine transferase identified from a Salmonella enterica isolate	MCR phosphoethanolamine transferase	peptide antibiotic	Peptide	Antibiotic target alteration	128	98	32	86	18	84	80	66	48	318	150	228	1336
ARO:3004691	MCR-3.4	A plasmid-mediated MCR-3.1 variant	MCR phosphoethanolamine transferase	peptide antibiotic	Peptide	Antibiotic target alteration	26	160	50	0	12	70	2	0	28	0	0	0	348
ARO:3004692	aadA10	aadA10 is a aminoglycoside nucleotidyltransferase gene	ANT(3'')	aminoglycoside antibiotic	Aminoglycoside	Antibiotic c	0	0	22	36	18	0	6	42	20	132	218	224	718

		encoded by plasmids in P. aeruginosa.				inactivation													
ARO:3004693	MCR-3.12	A plasmid-mediated phosphoethanolamine transferase and MCR-3 variant conferring resistance to colistin antibiotics.	MCR phosphoethanolamine transferase	peptide antibiotic	Peptide	Antibiotic target alteration	0	42	0	4	0	2	2	10	0	0	0	0	60
ARO:3004694	MCR-4.2	A plasmid-mediated MCR-4 variant which confers resistance to colistin antibiotics.	MCR phosphoethanolamine transferase	peptide antibiotic	Peptide	Antibiotic target alteration	230	18	44	2	12	182	4	4	40	2	6	0	544
ARO:3004695	MCR-4.3	A plasmid-mediated MCR-4 variant and colistin resistance gene from clinical Enterobacteriaceae	MCR phosphoethanolamine transferase	peptide antibiotic	Peptide	Antibiotic target alteration	2	2	0	0	2	10	6	4	4	2	0	0	32
ARO:3004696	MCR-4.4	A plasmid-mediated MCR-4 variant and colistin resistance gene	MCR phosphoethanolamine transferase	peptide antibiotic	Peptide	Antibiotic target alteration	434	202	114	1168	558	58	1552	1234	418	118	170	38	6064
ARO:3004698	MCR-5.2	A plasmid-mediated MCR-5 variant from Escheichia coli which confers resistance to colistin antibiotics.	MCR phosphoethanolamine transferase	peptide antibiotic	Peptide	Antibiotic target alteration	448	456	202	1164	532	178	1516	1280	480	1474	974	2058	10762
ARO:3004699	sta	Streptothricin acetyltransferase gene (STAT gene) that confers streptothricin resistance on Escherichia coli and Bacillus subtilis.	streptothricin acetyltransferase (SAT)	nucleoside antibiotic	Nucleoside	Antibiotic inactivation	0	0	4	36	0	4	14	20	0	48	128	62	316
ARO:3004715	vga(E) Staphylococcus cohnii	vga(E) gene variant that confers resistance to pleuromutilins, lincosamides and streptogramin A antibiotics in staphylococci	ABC-F ATP-binding cassette ribosomal protection protein	lincosamide antibiotic;macrolide antibiotic;oxazolidinone antibiotic;phenicol antibiotic;pleuromutilin antibiotic;streptogramin antibiotic;tetracycline antibiotic	Multidrug	Antibiotic target protection	84	158	128	80	32	144	78	76	36	132	158	90	1196
ARO:3004719	Tet(X3)	A tetracyline resistance gene located on an approximately 300-kb plasmid, designated p47AB. It inactivates all tetracyclines, including tigecycline, eravacycline, and	tetracycline inactivation enzyme	glycylcycline;tetracycline antibiotic	Tetracycline	Antibiotic inactivation	474	1026	970	1126	6586	622	1056	1868	5994	2600	2400	3166	27888

		omadacycline.Adjacent to insertion sequence ISVsa3 on the conjugative plasmid.																	
ARO:3004720	Tet(X4)	A tetracyline resistance gene located on an approximately 180-kb plasmid, designated p47EC. It inactivates all tetracyclines, including tigecycline, eravacycline, and omadacycline.Adjacent to insertion sequence ISVsa3 on the conjugative plasmid.	tetracycline inactivation enzyme	glycylcycline;tetracycline antibiotic	Tetracycline	Antibiotic inactivation	92	100	276	224	1012	106	204	616	1088	902	806	1012	6438
ARO:3004730	tva(A)	A pleuromutilin resistance gene encoding a predicted ABC-F transporter. The presence of inhibitory or sub-inhibitory concentrations of tiamulin showed that tva(A) confers reduced pleuromutilin susceptibility that does not lead to clinical resistance but facilitates the development of higher-level resistance via mutations in genes encoding ribosome-associated functions.	ABC-F ATP-binding cassette ribosomal protection protein	lincosamide antibiotic;macrolide antibiotic;oxazolidinone antibiotic;phenicol antibiotic;pleuromutilin antibiotic;streptogramin antibiotic;tetracycline antibiotic	Multidrug	Antibiotic target protection	984	1814	1170	696	482	1154	664	724	690	1100	886	994	11358
ARO:3004747	BAT-1	BAT-1 is a class D beta-lactamase found in Bacillus atrophaeus.	BAT Beta-lactamase	cephalosporin	Beta-lactam	Antibiotic inactivation	2	0	90	14	124	2	10	38	98	0	0	0	378
ARO:3004749	BCL-1	BCL-1 is a class A beta-lactamase found in Bacillus clausii.	BCL Beta-lactamase	penam	Beta-lactam	Antibiotic inactivation	20	0	0	0	0	30	0	0	0	0	0	0	50
ARO:3004755	BIL-1	BIL-1 is a plasmid-borne beta-lactamase that is also a class C beta-lactamase.	BIL Beta-lactamase	cephalosporin	Beta-lactam	Antibiotic inactivation	0	0	0	0	0	0	0	0	2	248	136	344	730
ARO:3004757	BKC-1	BKC-1 is a beta-lactamase conferring resistance to carbapenem and is found in Brazilian Klebsiella.	BKC Beta-lactamase	carbapenem	Beta-lactam	Antibiotic inactivation	0	0	14	12	12	6	56	24	30	30	16	4	204
ARO:3004761	BRO-1	BRO-1 is a class A beta-lactamase found in M.catarrhalis.	BRO Beta-lactamase	penam	Beta-lactam	Antibiotic inactivation	2	2	82	12	16	22	14	8	10	16	22	2	208

ARO:3004764	CBP-1	CBP-1 is a class A beta-lactamase gene found in C. botulinum.	CBP beta-lactamase	penam	Beta-lactam	Antibiotic inactivation	8	26	24	0	2	6	6	2	2	2	0	0	78
ARO:3004780	DES-1	DES-1 is a class A beta-lactamase gene found in Desulfovibrio desulfuricans.	DES beta-lactamase	penam	Beta-lactam	Antibiotic inactivation	0	0	0	0	0	0	6	0	0	0	0	0	6
ARO:3004802	GOB-10	GOB-10 is a class B beta-lactamase gene found in Chryseobacterium meningosepticum.	GOB beta-lactamase	carbapenem;cephalosporin;penam	Beta-lactam	Antibiotic inactivation	0	16	16	64	6	8	14	44	4	4	6	0	182
ARO:3004803	GOB-11	GOB-11 is a class B beta-lactamase gene found in Chryseobacterium meningosepticum.	GOB beta-lactamase	carbapenem;cephalosporin;penam	Beta-lactam	Antibiotic inactivation	2	4	2	0	8	2	2	0	2	26	58	48	154
ARO:3004804	GOB-12	GOB-12 is a class B beta-lactamase gene found in Elizabethkingia meningoseptica.	GOB beta-lactamase	carbapenem;cephalosporin;penam	Beta-lactam	Antibiotic inactivation	0	0	2	18	0	4	2	10	2	0	0	0	38
ARO:3004805	GOB-13	GOB-13 is a class B beta-lactamase gene found in Elizabethkingia meningoseptica.	GOB beta-lactamase	carbapenem;cephalosporin;penam	Beta-lactam	Antibiotic inactivation	332	144	66	666	304	32	1092	786	222	106	90	46	3886
ARO:3004807	GOB-15	GOB-15 is a class B beta-lactamase gene found in Elizabethkingia meningoseptica.	GOB beta-lactamase	carbapenem;cephalosporin;penam	Beta-lactam	Antibiotic inactivation	2	0	0	0	2	0	0	0	2	16	22	18	62
ARO:3004808	GOB-16	GOB-16 is a class B beta-lactamase gene found in Elizabethkingia meningoseptica.	GOB beta-lactamase	carbapenem;cephalosporin;penam	Beta-lactam	Antibiotic inactivation	18	50	30	44	32	48	56	86	44	66	94	62	630
ARO:3004810	GOB-3	GOB-3 is a class B beta-lactamase gene found in Elizabethkingia meningoseptica.	GOB beta-lactamase	carbapenem;cephalosporin;penam	Beta-lactam	Antibiotic inactivation	20	16	40	2	0	24	8	12	4	0	4	0	130
ARO:3004812	GOB-5	GOB-5 is a class B beta-lactamase gene found in Elizabethkingia meningoseptica.	GOB beta-lactamase	carbapenem;cephalosporin;penam	Beta-lactam	Antibiotic inactivation	0	32	0	0	0	4	2	6	0	0	0	0	44
ARO:3004813	GOB-6	GOB-6 is a class B beta-lactamase gene found in Elizabethkingia meningoseptica.	GOB beta-lactamase	carbapenem;cephalosporin;penam	Beta-lactam	Antibiotic inactivation	54	4	2	50	0	44	24	30	0	680	692	1210	2790
ARO:3004816	GOB-9	GOB-9 is a class B beta-lactamase gene found in Elizabethkingia meningoseptica.	GOB beta-lactamase	carbapenem;cephalosporin;penam	Beta-lactam	Antibiotic	6	42	20	0	4	0	8	4	4	112	102	134	436

						inactivation													
ARO:3004826	LAP-2	LAP-2 is an Ambler Class A beta-lactamase gene conferring resistance to quinolones.	LAP beta-lactamase	aminoglycoside antibiotic;fluoroquinolone antibiotic;rifamycin antibiotic;tetracycline antibiotic	Multidrug	Antibiotic inactivation	18	52	0	12	0	10	36	12	0	2	14	4	160
ARO:3004856	SCO-1	Narrow-spectrum beta-lactamase isolated from several Acinetobacter spp. isolates from Argentina, as well as E. Coli. Hydrolyzes penicillins at a high level and cephalosporins and carbapenems at a very low level	SCO beta-lactamase	cephalosporin;penam;penem	Beta-lactam	Antibiotic inactivation	0	0	6	0	0	0	0	0	0	6	10	14	36
ARO:3004870	NDM-27	A class B New Delhi metallo-beta-lactamase and NDM-1 variant	NDM beta-lactamase	carbapenem;cephalosporin;cephamycin;penam	Beta-lactam	Antibiotic inactivation	0	0	34	0	36	0	0	0	24	68	64	148	374
